# Schistosome W-Linked Genes Inform Temporal Dynamics of Sex Chromosome Evolution and Suggest Candidate for Sex Determination

**DOI:** 10.1093/molbev/msab178

**Published:** 2021-06-19

**Authors:** Marwan Elkrewi, Mikhail A Moldovan, Marion A L Picard, Beatriz Vicoso

**Affiliations:** 1 Institute of Science and Technology Austria, Klosterneuburg, Austria; 2 Skolkovo Institute of Science and Technology, Moscow, Russia; 3 CNRS, Biologie Intégrative des Organismes Marins (BIOM), Observatoire Océanologique, Sorbonne Université, Banyuls-sur-Mer, France

## Abstract

Schistosomes, the human parasites responsible for snail fever, are female-heterogametic. Different parts of their ZW sex chromosomes have stopped recombining in distinct lineages, creating “evolutionary strata” of various ages. Although the Z-chromosome is well characterized at the genomic and molecular level, the W-chromosome has remained largely unstudied from an evolutionary perspective, as only a few W-linked genes have been detected outside of the model species *Schistosoma mansoni*. Here, we characterize the gene content and evolution of the W-chromosomes of *S. mansoni* and of the divergent species *S. japonicum*. We use a combined RNA/DNA k-mer based pipeline to assemble around 100 candidate W-specific transcripts in each of the species. About half of them map to known protein coding genes, the majority homologous to *S. mansoni* Z-linked genes. We perform an extended analysis of the evolutionary strata present in the two species (including characterizing a previously undetected young stratum in *S. japonicum*) to infer patterns of sequence and expression evolution of W-linked genes at different time points after recombination was lost. W-linked genes show evidence of degeneration, including high rates of protein evolution and reduced expression. Most are found in young lineage-specific strata, with only a few high expression ancestral W-genes remaining, consistent with the progressive erosion of nonrecombining regions. Among these, the splicing factor *u2af2* stands out as a promising candidate for primary sex determination, opening new avenues for understanding the molecular basis of the reproductive biology of this group.

*Keywords:* sex chromosomes, evolutionary strata, W-linked gene, sex determining gene, schistosome parasites.

## Introduction

Separate sexes are frequently determined by a specialized pair of sex chromosomes (Charlesworth et al. 2005) called X and Y in species where males are heterogametic (e.g., in mammals), and Z and W when females comprise the heterogametic sex (e.g., in birds) (Bull 1983). Sex chromosomes arise from chromosomes containing sex-determining genes when parts of the sex-specific chromosome lose the ability to recombine (Ohno 1967; Nei 1969). Inefficient selection on this newly nonrecombining Y or W chromosomal region results in the accumulation of repetitive sequences and deleterious mutations, eventually leading to extensive gene loss (Bachtrog 2013). Loss of recombination can progressively spread to larger sections of the chromosome, yielding “evolutionary strata” that have started degenerating at different time points. Ancient Y/W chromosomes, such as those of mammals and birds, are typically gene-poor and heterochromatic. Some genes, however, such as those responsible for sex determination or sexual differentiation, as well as dosage-sensitive genes, can be preserved for large periods of time (Lahn et al. 2001; Charlesworth et al. 2005). Thus, identifying genes on these chromosomes can be an important step toward understanding the mechanisms underlying differences between the sexes (Lahn et al. 2001; Bellott et al. 2014).

Worms of the trematode genus *Schistosoma* cause schistosomiasis—one of the deadliest neglected tropical diseases, affecting hundreds of thousands of people in tropical regions. Many of the clinical symptoms of schistosomiasis, as well as the spreading of the parasite itself, are due to the massive egg production during the life-long mating between male and female worms (Kunz 2001; LoVerde et al. 2004), fueling a long-standing interest in their reproductive biology. Schistosomes are the only trematodes with separate sexes, which are determined by a pair of ZW sex chromosomes (Grossman et al. 1981; Lawton et al. 2011). Although homologous chromosomes correspond to the ZW pair in different schistosome lineages, there are substantial differences in the gene content of the Z-specific region of Asian (*Schistosoma japonicum*) and African (*S. mansoni* and *S. haematobium*) lineages, suggesting that recombination was lost between much of the sex chromosomes independently in the two clades (Picard et al. 2018).

Much less is known about the evolution and current gene content of the W-chromosome, and it is still unclear whether this chromosome plays a role in sex determination or differentiation. The latest version of the *S. mansoni* genome contains several large W-scaffolds which harbor 32 W-linked genes (Howe et al. 2016, 2017; https://parasite.wormbase.org/index.html, last accessed November 9, 2020), a much smaller number than the many hundreds annotated on the Z chromosome (Criscione et al. 2009; Protasio et al. 2012; Picard et al. 2018), consistent with widespread genetic degeneration. However, assembling W-derived sequences is difficult due to their heterochromatic and repetitive nature, and some W-genes may remain uncharacterized. Furthermore, only three W-linked genes have been identified in the Asian *S. japonicum* (Liu et al. 2020b) and none in other species for which draft genomes are available. This is an important gap, as any gene involved in sex determination is likely to be shared between different lineages, providing an important strategy for pinpointing promising candidates. Finally, the evolutionary history of the ZW pair in this clade, in which loss of recombination has occurred at different times in the two major lineages, offers a window into the temporal dynamics of degeneration of these nonrecombining W-chromosomes. In particular, a large section of the ZW pair, the “S0” stratum, is thought to have stopped recombining in the ancestor of all schistosomes (Picard et al. 2018). Two younger strata were formed independently in *S. mansoni* (S1man) and in *S. japonicum* (S1jap). In which of these strata W-linked genes are located, and whether they differ in their rates of evolution or patterns of expression depending on how long they have been nonrecombining, is still unknown.

Several studies have demonstrated that W-derived transcripts can be efficiently recovered by combining male and female DNA and RNA sequencing data (Moghadam et al. 2012; Cortez et al. 2014; Mahajan and Bachtrog 2017; Li et al. 2018). We perform the first such systematic characterization and comparison of W-derived transcripts in the divergent species *S. mansoni* and *S. japonicum*. We combine genomic and RNA-seq data spanning much of the parasite life cycle to detect dozens of candidate W-derived transcripts in each species, and characterize both their evolutionary history and patterns of expression throughout development. We discuss the relevance of these results to schistosome sexual differentiation, and to the evolution of ZW chromosomes in this group.

## Results

### Newly Identified W-Linked Genes in *S. mansoni* and *S. japonicum* Map Primarily to the Z

We applied a k-mer based pipeline to assemble female-specific transcripts (see Materials and Methods and supplementary fig. 1, Supplementary Material online). Similar approaches, in which male and female genomic reads are broken into shorter segments (k-mers), and k-mers found in only one sex are used to identify Y/W sequences, have been successfully applied to various organisms (Palmer et al. 2019). Our pipeline extends these by calling female-specific k-mers only if they are found in both DNA (one library per sex in each species) and RNA data (for each sex: one RNA-seq sample obtained by merging reads derived from three developmental stages for *S. japonicum*, and two samples merged from four stages for *S. mansoni*, see Materials and Methods and supplementary table 1, Supplementary Material online) and using them to output putative W-derived RNA-seq reads directly. Briefly, for each species, we selected k-mers that were found in all female DNA and RNA samples but in none of the male samples. RNA-seq reads containing these female-specific k-mers were extracted and assembled into putative W-transcripts. Male and female DNA reads were further mapped to putative W-transcripts longer than 200 base pairs (bp), and only transcripts with a high number of reads mapping perfectly in females but not in males were kept in our set of candidates (supplementary figs. 2 and 3, Supplementary Material online; specific steps to improve the assembly in *S. japonicum* are described in the Materials and Methods section).

We used BLAT (Kent 2002) to map our candidates to the gene models (CDS) of the *S. mansoni* genome (v7, supplementary table 2, Supplementary Material online) in order to assess the efficacy of our pipeline (as the *S. japonicum* genome is not assembled at the chromosome level): of the 86 *S. mansoni* candidate W transcripts (supplementary data 1, Supplementary Material online), 37 mapped to known protein coding genes (*S. mansoni* original W-candidates in table 1). The majority of these (24) mapped primarily to annotated W-linked genes in the current assembly (v7, obtained from female and male DNA), confirming the validity of our approach for detecting female-specific protein-coding sequences. Another nine mapped to Z-linked genes, and likely represent true W-genes which are missing from the current assembly. Finally, four mapped to genes in other chromosomal locations; these may represent W-linked genes that do not have a Z-homolog, or false positives. For the rest of our analyses, we combined our *S. mansoni* W-candidates with the annotated W-genes in this species (when a gene was found in both sets, only the longest transcript was kept) (*S. mansoni* combined W-candidates, including annotated genes in table 1 and supplementary data 2, Supplementary Material online), yielding a “combined” set of candidates of 90 transcripts, 42 of them protein coding. A similar number of *S. japonicum* W-candidates mapped to *S. mansoni* coding sequences (48 out of 94), all of which did so to known Z-linked genes, again confirming the efficacy of our pipeline for detecting W-linked protein coding genes (*S. japonicum* W-candidates in table 1 and supplementary data 3, Supplementary Material online).

**Table 1.** Number of Candidate W-Derived Transcripts, and the Genomic Location of Their Closest *Schistosoma mansoni* Homologs.

	Number of Candidates	Map to *S. mansoni* CDS (unique genes)	W Genes	ZW Genes	Other
*S. mansoni* original W-candidates	86	37 (28)	24 (15)	9 (9)	4 (4)
*S. mansoni* combined W-candidates, including annotated genes	90	42 (42)	30 (30)	8 (8)	4 (4)
*S. mansoni* combined set mapped to the [CDS without annotated W-linked genes]	90	40 (39)	NA	*34 (33)*	6(6)
*S. japonicum* W-candidates	94	48 (37)	0 (0)	*48 (37)*	0 (0)

Note.—“Unique genes,” in brackets, refers to the number of *S. mansoni* annotated genes to which a given set of candidates is mapping (several candidates can map to the same annotated gene). “ZW genes” refers to the ZW linkage group, and can correspond to either Z-specific genes or pseudoautosomal genes. The sets of ZW homologs that were used in downstream analyses are in italics.

In order to investigate the evolutionary history of these W-derived coding sequences, we further extracted their closest homologs in the genome. For *S. mansoni*, we remapped the candidate W-transcripts to the CDS set, after excluding annotated W-genes. We retrieved a close homolog for 40 out of the 42 protein-coding transcripts; 34 of them mapped to a homolog on the Z-chromosome, as expected if W- and Z-linked genes share a close ancestry (*S. mansoni* combined set mapped to the [CDS without annotated W-linked genes] in table 1). In the case of *S. japonicum*, we wanted to avoid possible ZW hybrid assemblies that may be present in the published genome. We therefore extracted the BLAT best hit of each protein-coding W-transcript from a male-derived transcriptome. The final list of protein coding W-candidates in the two species, as well as their respective homologs, is provided in supplementary data 4, Supplementary Material online. For the rest of the analysis, we focused on ZW homolog pairs (the 34 *S. mansoni* W-candidates with a Z-linked homolog, and the 48 *S. japonicum* W-candidates that mapped to Z-linked genes in *S. mansoni*, along with their homologs retrieved from the male transcriptome).

### Most W Genes Are Found in Younger Evolutionary Strata

**Fig. 1.**
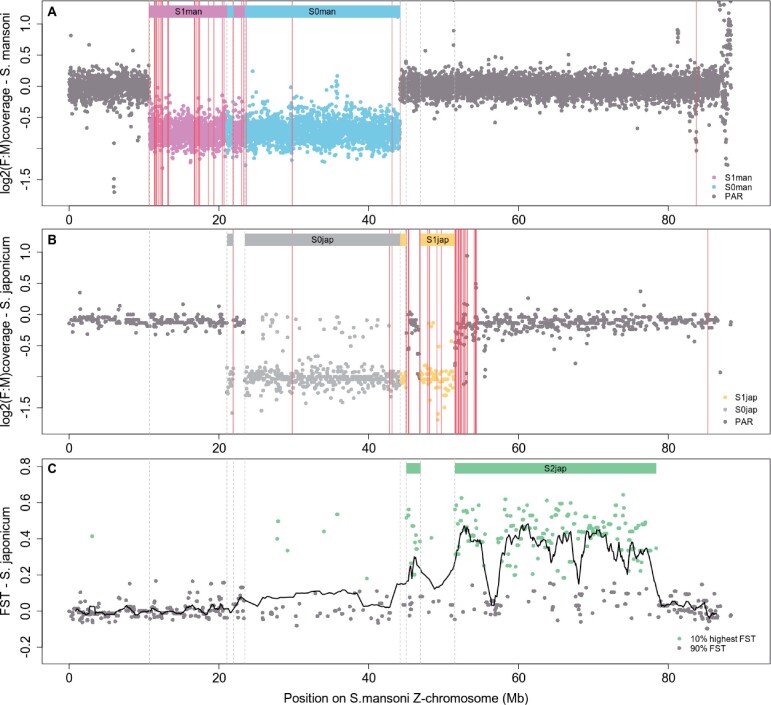
Updated evolutionary strata of schistosome sex chromosomes in *Schistosoma mansoni* and *S. japonicum*, and location of pairs of ZW homologous genes. Panels (*A*) and (*B*) show the log2 of the female-to-male ratio of coverage along the *S. mansoni* Z-chromosome (each dot represents either a 10 kb window of the *S. mansoni* genome, or a full scaffold of *S. japonicum*). Colored rectangles and dots show the various differentiated strata, and the data points included in each; the boundaries of the strata are further shown with light dotted gray lines. Parts of the Z that were differentiated in both species were assigned to the ancestral stratum (S0man in blue, and S0jap in gray), whereas lineage-specific regions of low female coverage were assigned to the younger S1man (in purple) and S1jap (in yellow). The locations of ZW pairs are shown in dark pink continuous lines. Panel (*C*) shows average scaffold *F*_ST_ values between males and females of *S. japonicum*: dots represent individual scaffolds, and the black line is the mean *F*_ST_ per sliding window of 20 genes. Values above the 90% percentile (*F*_ST_ > 0.178) of the genome are colored in green, and the corresponding putative coordinates of the very young S2jap stratum are denoted by the green rectangles.

Figure 1*A* and *B* shows the location of Z-linked genes for which at least one homologous W-transcript was detected in *S. mansoni* and *S. japonicum*, along with the location of the ancestral (S0) and younger lineage-specific strata of the sex chromosomes (S1man for *S. mansoni* and S1jap for *S. japonicum*, updated from (Picard et al. 2018) with the latest *S. mansoni* assembly v7) (supplementary data 5, Supplementary Material online). Very few S0 genes have a W-homolog (five in *S. mansoni* and four in *S. japonicum*, or 0.8% and 0.6% of the 635 annotated Z-specific genes identified in this stratum), as expected from an ancient degenerated nonrecombining region. Three of these are found in both species, consistent with the stratum being ancestral, and much of the gene loss occurring before the split of the two lineages. The proportion of W-genes that are preserved is higher for S1man (27 W- vs. 299 Z-linked genes, 9.03%, *P* value <0.0001 with a Fisher exact probability test) and S1jap (5 vs. 143, 4.2%, *P* = 0.013), suggesting that degeneration is ongoing in these strata.

Interestingly, many putative W-transcripts of *S. japonicum* mapped to a region of the Z that was previously classified as pseudoautosomal (PAR) (Picard et al. 2018 and fig. 1), suggesting that this region may have very recently stopped recombining in this species. Such regions are best detected using population genomic data, as the presence of genetic variants fixed on the W will lead to detectable levels of genetic differentiation between males and females (measured as the fixation index *F*_ST_). Although no such data is available for male and female *S. japonicum*, we used published genomic data from individual miracidia (Nikolakis et al. 2021), which we sexed based on their ratio of Z-to-autosome genomic coverage, as well as on the number of reads that mapped perfectly to W-transcripts (see Materials and Methods and supplementary fig. 4, Supplementary Material online). Single nucleotide polymorphisms were then called on the resulting 11 males and eight females, and mean *F*_ST_ between males and females was inferred for each scaffold (fig. 1*C* and supplementary fig. 5, Supplementary Material online; scaffold *F*_ST_ values are in supplementary data 6, Supplementary Material online). Most of the Z-chromosome to the distal end of S1jap showed consistently increased female–male *F*_ST_, confirming that this region corresponds to yet another young stratum of the ZW pair (now referred to as S2jap, a 28.8 Mb region that includes 720 genes). All W-transcripts that mapped to Z-linked scaffolds located in the *S. japonicum* PAR were consequently assigned to S2jap. Consistent with the comparable genomic coverage of the male and female samples in this region, a window-based Copy Number Variation (CNV) analysis inferred less than 1% of gene loss in the S2jap stratum, a number similar to that observed on the autosomes (supplementary table 3, supplementary data 7–9, Supplementary Material online).

### Patterns of Divergence of Old and Young W-Genes

The amount of synonymous divergence (estimated as d*S*, the number of synonymous substitutions per synonymous site) between Z- and W-homologs is proportional to how long they have been nonrecombining (Kimura 1987; Lahn and Page 1999; Nicolas et al. 2005; Bergero et al. 2007; Nam and Ellegren 2008; White et al. 2015). Consistent with the putative strata inferred from the coverage and *F*_ST_ analyses, S0 pairs have the highest d*S* in both species (median d*S* of 0.877 and 0.607 in *S. mansoni* and *S. japonicum*, respectively, supplementary data 4, Supplementary Material online). The median d*S* of the *S. mansoni* S1 stratum is lower than that of the *S. japonicum* S1 (0.176 vs. 0.465), suggesting that at least parts of it may have stopped recombining more recently. Finally, S2jap ZW homologs have the lowest median d*S* (0.085), in agreement with the most recent loss of recombination.

**Fig. 2.**
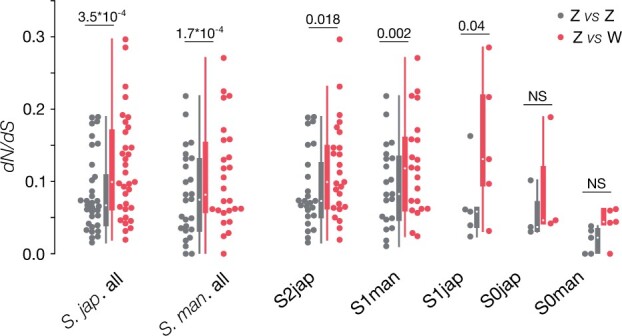
Distribution of d*N*/d*S* values between W-genes (Z vs. W, in red) or the corresponding Z-genes (Z vs. Z, in gray), and their Z-homologs in the other species. “*S. jap*.” refers to W or Z genes in *Schistosoma japonicum*, and “*S. man.*” refers to *S. mansoni* genes. Boxplots are shown for all ZW homologs (“*S. jap.* all” and “*S. man.* all”), and for ZW homologs located in individual strata. The significance of the difference between Z versus Z and W versus Z is shown above each boxplot (Wilcoxon tests).

W-linked genes typically show an increased ratio of nonsynonymous relative to synonymous divergence (d*N*/d*S*) compared with other genomic regions, consistent with the excessive accumulation of deleterious mutations (Bachtrog et al. 2008; Hough et al. 2014; Sigeman et al. 2019). To test for increased nonsynonymous divergence on the W, we estimated d*N* and d*S* between W-genes and their Z-homologs in the outgroup species (W vs. Z comparisons, e.g., *S. mansoni* W vs. *S. japonicum* Z), as well as between the corresponding Z-genes and their Z-homologs in the outgroup species (Z vs. Z) (fig. 2 and supplementary data 10, Supplementary Material online). As expected under degeneration of the W-chromosome, d*N*/d*S* values for W versus Z comparisons are higher than for Z versus Z comparisons (*P* < 0.001 in both species, paired Wilcoxon test). We also tested whether divergence patterns differ between W-genes (and their Z-homologs) located in older and younger strata. d*N*/d*S* values are lower for S0 genes than for genes in the other strata, for both Z versus Z comparisons (S0 median of 0.02 in *S. mansoni*, compared with 0.08 for S1man, *P* = 0.002 with a Wilcoxon test; in *S. japonicum*, the medium d*N*/d*S* is 0.04 for S0 vs. 0.06 for S1 and 0.07 for S2 genes; the difference is not significant, but there are only three genes in the S0 in this species) and for Z versus W comparisons (*S. mansoni*: S0 median of 0.04, S1man median of 0.12, *P* value = 0.003, Wilcoxon test; *S. japonicum*: S0 median of 0.05 vs. S1jap median of 0.13 and S2jap of 0.10, difference not significant). This is consistent with genes with important functions being maintained over long periods of time, as has been observed in mammals and birds (Bellott et al. 2014; Sigeman et al. 2019; Xu et al. 2019; Bellott and Page 2021).

### Patterns of Expression of W-Candidates

Patterns of expression can be used as an additional measure of functional constraint, as essential genes tend to have high and broad expression (Liao et al. 2006; Wang et al. 2015; Kabir et al. 2017; Fraïsse et al. 2019). Furthermore, the first evidence of genetic degeneration of Y/W-linked genes is often a decrease in expression relative to that of the X/Z (Zhou and Bachtrog 2012; Gammerdinger et al. 2014; Hough et al. 2014; Pucholt et al. 2017). We therefore estimated gene expression levels (in transcripts per million, TPM) using published *S. mansoni* and *S. japonicum* male and female expressions at different developmental time points, to investigate differences in the expression patterns of ZW gene pairs between strata, and to compare the expression of W-linked genes with their Z-linked counterpart (supplementary data 11 and 12, Supplementary Material online). Reads were mapped to curated transcriptomes that included our W-candidates (see Materials and Methods) using Kallisto, an RNA quantification program capable of inferring paralog/allele-specific expression (Bray et al. 2016). An overview of gene expression of all protein-coding W-candidates is provided in supplementary figures 6 and 7, Supplementary Material online, and shows that the majority of them are expressed in at least some female stages (but not or much less expressed in males, confirming that we are for the most part correctly discriminating between Z- and W-derived expression of ZW gene pairs).

**Fig. 3.**
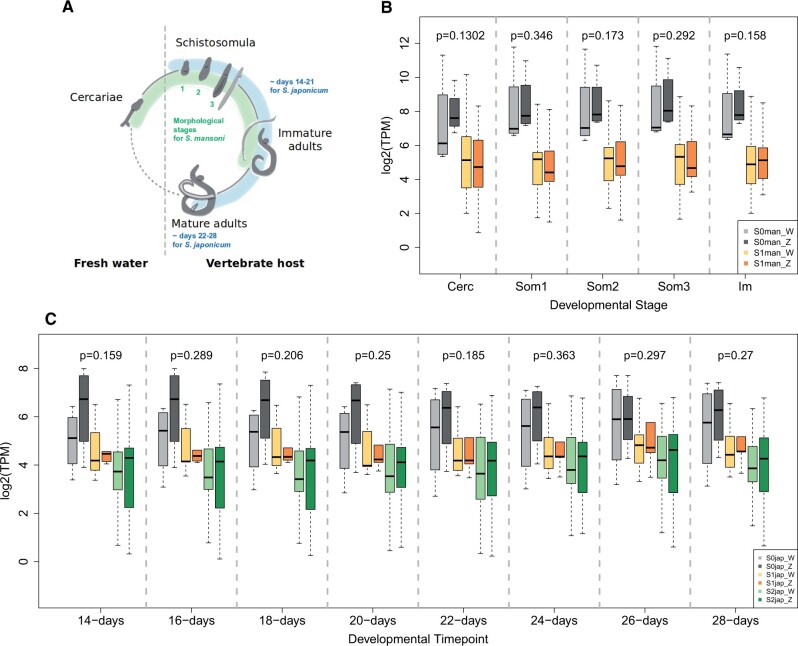
Distribution of S0, S1, and S2 W- and Z-gene expression throughout female development. Panel (*A*) shows a simplified schematic of the life cycle of schistosomes, depicting the larval stage in fresh water and the process of sexual maturation in the vertebrate host. The part of development represented in the expression data sets is shown in green for *Schistosoma mansoni*, and blue for *S. japonicum*. An additional data set with *S. mansoni* mature adults is plotted in supplementary figure 8, Supplementary Material online. Panel (*B*) shows gene expression values (TPM) for the *S. mansoni* W-candidates and their Z-homologs according to their respective strata (S0 in gray, S1 in orange) across five developmental stages: cercariae “Cerc,” three subsequent schistosomulum stages “Som1-3” and immature adult schistosomes “Im.” Panel (*C*) shows gene expression values (TPM) for the *S. japonicum* W-candidates and their Z-homologs according to their respective strata (S0 in gray, S1 in orange, S2 in green) across eight different developmental time points (in days postinfection). *P* values above each boxplot denote the significance of the difference in expression between W- and Z-derived transcripts, considering all strata together (Wilcoxon test).

Figure 3 shows the distribution of female expression levels of W-candidates from the different strata and those of their Z-homologs (when more than one W-candidate mapped to the same Z-linked gene, only the longest was kept; see supplementary fig. 8, Supplementary Material online, for an additional *S. mansoni* RNA-seq data set; only transcripts with TPM > 1 were considered). The median W:Z expression ratio is below 1 for all sampled time points in both species (supplementary figs. 9–11, Supplementary Material online), but these differences are not significant (*P* values in fig. 3*B* and *C* for all ZW pairs, and supplementary figs. 9–11, Supplementary Material online, for individual strata). Although we may simply lack power, as the number of genes for which we can perform comparisons is small (especially for the S0), this may also suggest that there is significant selective pressure against loss of expression of genes maintained on the W. The median expression of S2jap W transcripts is also below the median Z expression at every available time point (although this is again not significant for individual comparisons, supplementary fig. 9, Supplementary Material online), consistent with some loss of expression occurring early in sex chromosome evolution. Finally, although direct comparisons of the gene expression of W-candidates (or Z-homologs) between strata are only significant for some developmental stages (Kruskal–Wallis test, supplementary tables 4–6, Supplementary Material online), S0 W-linked genes and their Z-linked homologs have the highest median expression level at all time points available for both species (fig. 3), providing further support for their functional importance (and/or potentially contributing to the differences in d*N*/d*S* observed between strata).

### Shared Ancestry Suggests Candidate for Sex Determination

**Fig. 4.**
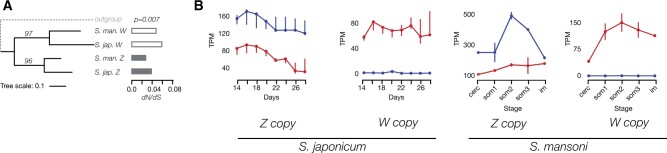
Evolution and expression of the shared S0 gene *u2af2*. Panel (*A*) shows the gene tree with bootstrap values. Terminal-branch specific d*N*/d*S* values along with the χ^2^*P* values of the deviations of observed values from the uniform assumption are shown as histograms. White bars portray d*N*/d*S* of W-specific genes, gray bars show d*N*/d*S* values of the Z-copies. Panel (*B*) shows gene expression values (TPM) of Z- and W-copies of *u2af2* on different developmental stages of *Schistosoma japonicum* and *S. mansoni*. The spread between the lowest and the highest value among the replicates is shown with error-bars, medians are shown with dots. Stages in *S. mansoni*: “cerc” means cercariae, “som1-3” are three subsequent schistosomula stages and “im” stands for immature adults. Red and blue lines show TPM values of females and males, respectively.

Although the master switch of sex determination may have changed since the split of the Asian and African schistosome lineages (see Discussion), W-linked genes that are shared between the two are promising candidates. If the two lineages still share the ancestral sex-determining gene, this gene should: 1) show a clear phylogenetic clustering of the W-copies from the two species, consistent with ancestral W-linkage; 2) have a low d*N*/d*S* value, supporting functional conservation; and 3) show expression in females of the two species during sex determination. Figure 4 and supplementary figure 12, Supplementary Material online, show the phylogeny, branch-specific d*N*/d*S* and patterns of expression throughout development for the three W-linked genes (and their Z-homologs) that were found in both species: a U2 snRNP auxiliary factor large subunit (*u2af2*, OrthoGroup OG0000710 in supplementary data 4, Supplementary Material online), a ubiquitin conjugating enzyme variant (*uev*, OrthoGroup OG0000869), and an ankyrin repeat and KH domain-containing protein 1 (*ankhd1*, OrthoGroup OG0000874). Of the three, *u2af2* stands out as fitting all three predictions (fig. 4), as it has high expression throughout female development and d*N*/d*S* below 0.1 in both species. It is also the only W-candidate in either species with the term “female sex differentiation” in its functional annotation (supplementary data 13 and 14, Supplementary Material online), further strengthening the case for its role in determining sex.

The *S. mansoni* W-copy of *ankhd1* is much shorter than the Z-copy, or than the *S. japonicum* Z- and W-homologs, and it has a higher branch-specific d*N*/d*S* than any of its homologs, consistent with loss of function. It also shows no expression at any female stage of *S. mansoni*, making it an unlikely candidate for sex determination. Phylogenetic clustering of Uev homologs occurs by species rather than by chromosome, suggesting that it was not on the W-chromosome before the split of the two clades, again arguing against an ancestral sex-determining function.

## Discussion

### An Efficient k-mer Pipeline for Detecting W-Transcripts at All Levels of Divergence

Y and W chromosomes are notoriously difficult to study, and were largely excluded from early genome projects. Many bioinformatics approaches have since been developed to identify Y/W-derived sequences from next-generation sequencing data, typically either based on differences in male and female DNA/RNA-seq coverage of Y/W-derived transcripts (Cortez et al. 2014; Zhou et al. 2014; Smeds et al. 2015), or on differences in k-mer frequencies between male and female samples (Carvalho and Clark 2013; Tomaszkiewicz et al. 2016; Li et al. 2018; Rangavittal et al. 2018, 2019). Because they require that Y/W-reads do not map to X/Z-derived sequences, coverage-based approaches are more suitable to identify highly differentiated sex chromosomes (our own attempt at implementing such an approach in *S. japonicum* yielded only a few candidate W-transcripts, data not shown). Multiple k-mer based approaches have been used to identify and/or assemble W- and Y-specific genomic contigs and transcripts. Early approaches required a genome or transcriptome assembly obtained from the heterogametic sex (Carvalho and Clark 2013; Tomaszkiewicz et al. 2016; Rangavittal et al. 2018, 2019). These were also best suited to identify differentiated sex-linked sequences, as Y/W sequences do not necessarily assemble into separate scaffolds when they are very similar to X/Z regions. More recently, k-mer based approaches have been used to first extract DNA reads that contain a large fraction of sex-specific k-mers, which are then assembled separately from reads derived from the rest of the genome, directly yielding candidate Y/W sequences (Tomaszkiewicz et al. 2016; Li et al. 2018). Such read-selecting approaches have been successfully applied to the differentiated sex chromosomes of Gorilla and Human (Rangavittal et al. 2019), *Bombyx mori* (ZW) (Katsuma et al. 2018), *Drosophila melanogaster* (XY), and *Anopheles gambiae* (XY) (Li et al. 2018) but also to the very young Y chromosome of two guppy species (Morris et al. 2018). This encouraged us to use a similar strategy to tackle the unique evolutionary history of the W chromosome in schistosomes. Since we were primarily interested in finding W-linked genes, only k-mers that were found in both female DNA-seq and RNA-seq data (but not in male DNA or RNA data) were classified as female-specific, and used to select and assemble RNA-seq reads directly into a set of W-specific transcripts. This has several advantages: 1) it reduces the need for extensive genomic data when RNA samples are available; 2) it reduces the number of sex-specific k-mers that must be dealt with, making the pipeline more efficient; 3) only putative Y/W-derived RNA-seq reads are assembled into transcripts, avoiding issues of repetitive sequences and hybrid assemblies between homologous genes when the sex chromosomes are poorly differentiated. Our final set of candidates included ancestral W-linked genes that were highly differentiated from their Z-homologs, but also uncovered a new evolutionary stratum of the *S. japonicum* ZW pair that could not be detected with coverage approaches (Picard et al. 2018), demonstrating the power of this method for studying sex-specific sequences in species that have varying levels of sex chromosome differentiation, or for which such information is missing. Finally, our pipeline is based on the published and efficient k-mer manipulation package BBMAP (Bushnell 2014), making it easy to implement for any organism for which male and female data are available, even in the absence of a reference genome.

### Temporal Dynamics of W Degeneration

Schistosome sex chromosomes have various evolutionary strata that differ between the closely related *S. mansoni* and *S. japonicum*, allowing us to probe the evolution of W-genes at different timepoints after the loss of recombination. In particular, with the inclusion of the very young S2jap, a very broad timeline of sex chromosome evolution is represented in this group. Similar analyses have been performed in species (or species groups) that have XY systems/strata of varying ages (Bachtrog 2013; Hough et al. 2014; Schultheiß et al. 2015; Crowson et al. 2017), but are mostly lacking in species with ZW chromosomes, for which information on early and late sex chromosome evolution typically come from different lineages (but see Sigeman et al. [2019] for an investigation of the multiple neo-sex-chromosomes of lark birds). Our results illustrate in one clade the insights on sex chromosome evolution gained from these various organisms (Kaiser et al. 2011; Bachtrog 2013; Hough et al. 2014; Bellott et al. 2017; Crowson et al. 2017). Soon after recombination is lost on the W, genes that are under weak purifying selection start accumulating nonsynonymous mutations, and their expression decreases relative to that of their Z-homologs (Kaiser et al. 2011; Hough et al. 2014). Over time, these genes are lost, and only increasingly important (and highly expressed) genes are maintained on the W (Bellott et al. 2014; Crowson et al. 2017). Finally, the few genes that remain on very ancient strata become stably maintained over long periods of time (Bachtrog 2013).

The presence of old and young strata makes schistosomes a promising model for studying how such a stepwise loss of recombination can occur. Local loss of recombination between sex chromosomes was originally thought to be driven by inversions, as these prevent homologous pairing and crossing over during meiosis (Charlesworth et al. 2005). Although it is supported by the order of XY gene pairs on mammalian sex chromosomes (Lemaitre et al. 2009), and inversions have been detected on young sex chromosomes (Wang et al. 2012; Roesti et al. 2013), this model has been brought into question by the discovery of unstable boundaries between the recombining and nonrecombining regions of sex chromosomes (Cotter et al. 2016; Campos et al. 2017; Wright et al. 2017). Instead, changes in epigenetic state, potentially driven by the accumulation of transposable elements, may first repress recombination (Lepesant et al. 2012), with inversions accumulating later (Charlesworth 2017; Furman et al. 2020). A chromosome-level assembly of the *S. japonicum* and *S. mansoni* W and Z, as well as a thorough investigation of their chromatin state (Picard et al. 2019), will make it possible to compare ancestral and derived gene order and chromatin landscape for S1 and S2 strata, and potentially provide answers as to how ZW recombination was repressed in this clade.

### Sex Determination in African and Asian Schistosomes

Our comparative analysis of the content of the W-chromosome of the two main schistosome lineages yielded very few genes shared between them, one of them an interesting candidate for sex determination: *u2af2* , a conserved house-keeping gene involved in pre-mRNA splicing from yeast to * Drosophila* (Kanaar et al. 1993; Potashkin et al. 1993). Although* u2af2* does not play a direct role in sex determination in other clades, homologs are known to be involved in meiosis-mitosis fate decision in *C. elegans* (*uaf-1*) (Kerins et al. 2010), and in sex-specific splicing in insects (*U2af50*) (Verhulst et al. 2010). A W-linked copy of *u2af2* was previously identified in *S. japonicum* (Liu et al. 2020b), and is highly expressed throughout the female life cycle in the two schistosome species (fig. 3; Liu et al. 2020b). We hypothesize that the W copy of *u2af2* may have been co-opted for sex determination, whereas its homolog on the Z retained the ancestral general pre-mRNA splicing function. The identification of this candidate is based on the assumption that the sex determination pathway is conserved in schistosomes. Since the African and Asian groups share part of the ZW pair, they must have ancestrally had the same sex-determining master switch, but whether this is still true is at this point unclear, as no gene has been functionally linked to primary sex determination in either species (Wang et al. 2019). Although estimates of the age of the clade vary widely (Lawton et al. 2011), the fact that new strata have become differentiated in each lineage, and that the median rate of synonymous divergence between them is substantial (65%, Picard et al. 2018) suggests that they have been separated for long enough that turnover of the sex determination switch could have occurred. On the other hand, the sexual development of males and females is similar in the two species, and narrowing the search to the few shared W-linked genes seems like a reasonable first step. Interestingly, the two other shared candidates, *ankhd1* and* uev* , both show some similarity to genes that are part of—or interact with—the sex determination pathway of nematodes. The *Caenorhabditis elegans* sex determination gene *fem-1* contains ankyrin repeats, similar to our W-candidate *ankhd1* (Spence et al. 1990). *fem-1* interacts with a ubiquitin ligase to regulate *tra* , the terminal effector of sex determination (Starostina et al. 2007); our candidate *uev* encodes an E2 ubiquitin-conjugating enzyme, which is required by ubiquitin ligases to mark target proteins for degradation. Aside from providing other possible candidates for sex determination, the fact that the three shared W-candidates show similarity to genes with sex-related functions supports the idea that genes that are maintained on W-chromosomes for long periods of time may be likely to perform female functions. Finally, we only focused here on genes with homology to known protein-coding genes. Noncoding transcripts and microRNAs present on the W-chromosome could also play a role in sex determination and differentiation (Marco et al. 2013; Kiuchi et al. 2014; Zhu et al. 2016; Maciel et al. 2020), and need further investigation.

## Materials and Methods

### Data

All analyses were performed on publicly available data. The *S. mansoni* genomic libraries were downloaded from Bioproject PRJEB2320 (Wellcome Sanger Institute, Protasio et al. 2012), and the *S. japonicum* genomic libraries from the three following BioProjects: PRJNA432803 (IST Austria, Picard et al. 2018), PRJNA354903 (Wuhan University), PRJNA650045 (University of Texas at Arlington, Nikolakis et al. 2021). The RNA-seq libraries for *S. mansoni* were downloaded from Bioprojects PRJNA312093 (University of Perpignan *Via Domitia,*Picard et al. 2016) and PRJEB1237 (Wellcome Sanger Institute, Lu et al. 2016; Lu et al. 2017), and the *S. japonicum* RNA-seq libraries from PRJNA343582 (National Institute of Parasitic Diseases, Wang et al. 2017), PRJNA252904 (Chinese Academy of Medical Sciences and Peking Union Medical College, Piao et al. 2014), and PRJNA504625 (Wuhan University, Liu et al. 2020a). A detailed list of individual accession numbers, and of the steps for which they were used, is provided as supplementary table 1, Supplementary Material online.

### k-mer Based Assembly of W-Linked Transcripts

#### k-mer Analysis

For each species, we used one female and one male genomic library, as well as one (*S. japonicum*) or two (*S. mansoni*) replicates of pooled female RNA-seq libraries, and several individual male RNA-seq libraries. In the case of *S. mansoni*, the two female RNA-seq replicates were made by pooling the first replicates (PRJNA312093) of female cercariae, schistosomula, immature, and mature adult libraries together, and the second replicates in the same way (such that transcripts with stage-specific expression are represented in each of the samples). In the case of *S. japonicum*, a single RNA-seq pool was made by merging two female schistosomula libraries (PRJNA343582) and one mature female adult library (PRJNA252904). In both species, the equivalent RNA-seq libraries were available for males (*S. mansoni*: two replicates each of the three developmental stages, PRJNA312093, and of mature adults, PRJEB1237; *S. japonicum*: one replicate each of two schistosomula stages, PRJNA343582, and mature adults, PRJNA252904), and used individually.

The read libraries for both species were trimmed with the Trimmomatic package (Bolger et al. 2014). Our k-mer based pipeline utilizes the tools included in the BBMap package (Bushnell 2014), and was run separately for each species. kmercountexact.sh was first used to output the unique 31 base pair k-mers in each of the female libraries separately, yielding one set of unique k-mers per female RNA/DNA library. We then used the same function to extract the k-mers that are shared between all the female DNA and RNA k-mer sets, by setting the mincount parameter to the total number of libraries. The resulting set of shared female k-mers was then filtered by removing any 31-mers found in any of the male DNA and RNA libraries using bbduk.sh. This set of female-specific 31-mers was then used as input for bbduk.sh to recover female RNA reads with at least 40% female-specific k-mers (“minkmerfraction” parameter set to 0.4; this threshold is not very stringent in order to allow for the transcripts to assemble properly, but requires downstream filtering of assembled transcripts). Those reads were then assembled using SOAPdenovo-trans (Xie et al. 2014), and fafilter (UCSC source code collection) was used to remove all transcripts shorter than 200 bp in both species.

#### Filtering the *S. mansoni* Candidates

Bowtie2 (Langmead and Salzberg 2012) (options –no-unal –no-hd –no-sq) was used to map the female (ERR562990) and male (ERR562989) genomic reads to the output of the k-mer pipeline (175 transcripts). The number of perfectly matching male and female reads (reads with 0 edit distance “NM:i:0”) to each transcript were counted. All transcripts that had fewer than 20 perfectly matching female reads and/or a ratio of (male:(male + female)) perfect matches of more than 0.1 were removed. In order to have a more comprehensive set of W-transcripts for downstream analyses, the coding sequences of the 32 annotated W genes were added to our set of candidates. In order to collapse transcripts that were both in the k-mer and annotated sets, we then used a perl script (SpliceFinder.pl) to produce clusters of transcripts with >100 bp of alignment and less than 1% divergence (putative isoforms), and kept only the longest isoform per cluster. The final set had 97 W-candidates.

#### Filtering and Improvement of Assembly of the *S. japonicum* Candidates

Bowtie2 (options –no-unal –no-hd –no-sq) was used to map a new set of four male only genomic libraries (SRR5054524, SRR5054649, SRR5054671, SRR5054674) and three mixed (males + females) libraries (SRR5054672, SRR5054673, SRR5054701) to the output of the k-mer pipeline (1,041 transcripts). All transcripts with a sum of perfect matches from the mixed genomic libraries of less than 15 reads and/or a ratio of (male:(male + mixed)) of less than 0.1 were removed. In order to obtain longer sequences for downstream analyses of the resulting 157 *S. japonicum* candidates, we originally mapped them to annotated *S. japonicum* coding sequences (https://parasite.wormbase.org/index.html, last accessed November 9, 2020; Howe et al. 2016, 2017): these appeared to often contain hybrids of W-linked genes and of their Z-homologs (as parts of it were completely identical to our candidates whereas other parts were clearly diverged, data not shown). We therefore mapped our candidates to a long k-mer female transcriptome assembly (SOAPdenovo-trans assembly of all reads in Bioproject PRJNA343582, with *K* = 65, supplementary data 15, Supplementary Material online), which should be largely devoid of ZW hybrid assemblies. W-candidates were mapped to the transcriptome with BLAT, and scaffolds with a minimum match score of 50 and less than 1% divergence were retrieved. Cap3 (Huang and Madan 1999) was used to further assemble them. We merged the output of Cap3 with our 157 transcripts and used Splicefinder.pl to keep the longest transcript per gene. We once again mapped the four male-only samples and the three mixed samples to the resulting set and followed the same filtering approach described above, yielding a final set of 96 W-specific candidates.

To identify the Z-homologs for the *S. japonicum* W-candidates, we assembled a male transcriptome from male reads (SRR8175618, supplementary data 15, Supplementary Material online). The reads were trimmed with the Trimmomatic package (Bolger et al. 2014) and then assembled using Trinity (Grabherr et al. 2011) followed by Cap3 (Huang and Madan 1999). We mapped our candidates to the male assembly using BLAT (with a translated query and database and a minimum match score of 50), and selected only the transcript with the highest match score for each W-candidate, which was used as its homolog. The completeness of both the female and male transcriptomes was assessed using BUSCO (Simão et al. 2015; Waterhouse et al. 2018) in the transcriptome mode with the metazoa-specific set (metazoa_odb10) and compared with the BUSCO scores for the published *S. japonicum* and *S. mansoni* transcriptomes (supplementary fig. 13, Supplementary Material online).

### Mapping of W-Candidates to *S. mansoni* Coding Sequences

We mapped the filtered sets of candidates of both species to the version 7 *S. mansoni* coding sequences (https://parasite.wormbase.org/index.html; Howe et al. 2016, 2017) using BLAT with a translated query and data set, and a minimum match score of 100 (table 1). Only the CDS with the highest matching score was kept for each candidate. In the case of *S. mansoni*, we performed three different mappings: 1) the filtered original candidates against the full set of *S. mansoni* CDS; 2) the final combined set against the full *S. mansoni* CDS; 3) the final combined set against the *S. mansoni* CDS, but with all the different transcript isoforms of the 32 annotated W genes removed using a perl script, in order to detect close homologs of W-linked genes in the genome (table 1).

### Definition of Shared and Lineage-Specific Strata Based on Coverage Analysis

#### *De Novo* Determination of Z-Specific Regions in *S. mansoni*

Z-specific regions were determined for the latest version of the S. *mansoni* genome based on female and male genomic coverage, following the bioinformatic pipeline described in Picard et al. (2018). The reference genome, coding sequences (CDS), and their respective chromosomal locations (BioProject PRJEA36577, version WBPS14) were obtained from the WormBase Parasite database (https://parasite.wormbase.org/index.html, last accessed November 9, 2020; Howe et al. 2016, 2017), on October 30, 2020. We estimated the genomic coverage for females (ERR562990) and males (ERR562989) by mapping the DNA reads to the genome with Bowtie2 (v2.3.4.1) and by using the uniquely mapped reads as SOAPcoverage input (SOAPcoverage v2.7.7). The coverage analysis was performed for windows of 10 kb. We classified as pseudoautosomal (PAR) any window on the ZW linkage group that had a F:M coverage ratio higher than the 2.5 percentile of the autosomes. The remaining windows were considered to be putatively Z-specific. The 1% of Z-specific windows with the highest F:M coverage ratio were further marked as “ambiguous” and not considered for strata assignments (supplementary fig. 14, Supplementary Material online). The classification of Z-specific and PAR regions, and of the CDS that they contain, is reported in supplementary data 5, Supplementary Material online.

#### Inference of the Location of *S. japonicum* Scaffolds along the *S. mansoni* Z-Chromosome

*Schistosoma japonicum* reference genome were obtained from the WormBase Parasite database (https://parasite.wormbase.org/index.html, last accessed November 9, 2020; Howe et al. 2016, 2017), on October 30, 2020 (BioProject PRJEA34885, version WBPS14). We mapped *S. mansoni* CDS to *S. japonicum* scaffolds using BLAT (Kent 2002) with a translated query and database. The BLAT alignment was then filtered to keep only the mapping hit with the highest score for each *S. mansoni* CDS. In a second filtering step, when several *S. mansoni* genes overlapped on the *S. japonicum* genome by more than 20 bp, we kept only the highest mapping score. The position of each *S. japonicum* scaffold on the *S. mansoni* Z-chromosome was inferred from the position of the *S. mansoni* genes that mapped to it.

#### Strata Definition

The coverage-based assignment of *S. japonicum* scaffolds to Z-specific (Z) or pseudoautosomal (PAR) regions was obtained from Picard et al. (2018), and is reported in supplementary data 5, Supplementary Material online. The de novo strata assignment shown in figure 1 was based on the comparison of the classification of the genomic location of each *S. mansoni* gene as Z-specific or PAR, and the classification of the scaffold that their CDS mapped to in *S. japonicum* (see above). Genes were assigned to the stratum S1man if they were located in a Z-specific window in *S. mansoni* but their CDS mapped to a scaffold classified as PAR in *S. japonicum*, and vice versa for S1jap. Genes that mapped to Z-specific genomic regions in both species were assigned to the stratum S0. Finally, the analysis of *F*_ST_ between males and females (see below) revealed a new stratum specific to the *S. japonicum* lineage (S2jap). Genes were assigned to this stratum if they mapped to windows/scaffolds classified as PAR in both species, but the mean *F*_ST_ value of the *S. japonicum* scaffold was above the 90th percentile of the distribution of the entire genome. Final coordinates of the strata were defined by the first base of the first *S. mansoni* CDS assigned to a specific stratum and the last base before the start of the first gene assigned to a different stratum (supplementary data 5, supplementary table 7, Supplementary Material online). More than five consecutive assignments to a given region were needed to change strata. If in those five first CDS one was assigned as “ambiguous,” the original assignment of the windows was considered.

### *F*_ST_ Analysis

We downloaded whole-genome sequencing data of 22 *S. japonicum* individual miracidia samples (PRJNA650045). The sex of the samples was not identified, so we used Bowtie2 (v2.3.4.1, –end-to-end –sensitive mode) to map the reads to the *S. japonicum* genome. The resulting SAM files were filtered to keep only uniquely mapped reads, and the genomic coverage for each scaffold was estimated from the filtered SAM files with SOAPcoverage (v2.7.7). We used the output to calculate the median Z-to-Autosomal genomic coverage for each of the samples based on the scaffold assignments from Picard et al. (2018). Furthermore, we used bowtie2 (–no-unal –no-hd –no-sq) to map the forward reads of all the samples to our W-candidates and three randomly chosen autosomal controls; we filtered for 0 edit distance “NM:i:0” to find the number of perfectly matched reads. We removed three of the samples due to low coverage, and the remaining 19 were identified as 11 males (Log2(Z:A) > 0.9) and eight females (Log2(Z:A) < 0.75 and ratio of reads mapping to W vs. autosomal controls >100, supplementary fig. 4, Supplementary Material online).

We used Bowtie2 to index the *S. japonicum* genome and map the genomic reads from the 19 libraries to it (–end-to-end –sensitive mode). The SAM files were converted to sorted BAM files using SAMtools (Li et al. 2009). We detected the genetic variants (SNPs) in the samples using the BCFtools mpileup function (Li et al. 2009), filtered the output for minimum and maximum read coverage and mapping quality using VCFtools (Danecek et al. 2011), and removed multiallelic sites using BCFtools. The calculation of the per-site *F*_ST_ between males and females was performed using VCFtools, and the output was used to calculate the mean *F*_ST_ values between the male and female samples for each scaffold.

### CNV Analysis in *S. japonicum* Female

After read mapping with Bowtie2 against *S. japonicum* genome, Copy Number Variation (CNV) analysis was performed using the control-FREEC prediction tool (Boeva et al. 2012), for genomic windows of 1, 5, and 10 kb, testing the female genomic library (SRR6841388) against the male genomic library as reference (SRR6841389). Only deletions with statistical support (Wilcoxon *P* value <0.05) were further analyzed. *S. japonicum* genes that overlapped with a deleted window were classified as putatively lost (supplementary data 7–9, Supplementary Material online).

### Estimating the Rates of Evolution of ZW Homologs

The coding sequences of W-candidates of both species (and of their closest homologs in *S. japonicum*) were obtained with the command-line version of Genewise (Wise2 package v2.4.1, Birney et al. 2004), using the protein sequence of the closest *S. mansoni* homologs (see Mapping of W-Candidates to S. mansoni Coding Sequences) as input. The coding sequences of W-candidates and their closest homologs (within the same species) were aligned with the TranslatorX package (Abascal et al. 2010) with the “gblocks” option to filter out unreliable sections of the alignment. The d*N* and d*S* values were obtained with KaKs_calculator2.0 (Wang et al. 2010) using the Yang Nielsen algorithm (YN). For the d*N*/d*S* and d*S* between ZW pairs of *S. mansoni* and *S. japonicum* (fig. 2*A*), we only considered the KaKs_calculator2.0’s estimates for pairs with 300 sites or more, and d*S* above 0 (supplementary data 4, Supplementary Material online).

To estimate d*N* and d*S* between W-genes and their Z-homologs in the outgroup species (W vs. Z comparisons), as well as between the corresponding Z-genes and their Z-homologs in the outgroup species (Z vs. Z), we considered only the instances in which the ZW pair and at least one sequence from the outgroup species clustered in the same Orthogroup (see Identifying the Shared Candidates Using OrthoFinder). When multiple orthologs were present in the outgroup species, the sequence with the highest BLAT match score to the W-candidate was used. Sequence alignments (of the three sequences) and pairwise d*N* and d*S* estimation were then performed as before (supplementary data 10, Supplementary Material online).

To obtain branch-specific estimates of d*N*/d*S* for W-candidates shared between the two schistosome lineages (fig. 4), we aligned W- and Z-homologs from both species, as well the closest *Clonorchis sinensis* sequence (an outgroup of schistosomes) placed in the respective Orthogroup (see Identifying the Shared Candidates Using OrthoFinder). Trees were constructed with the maximum likelihood method (Felsenstein 1973) implemented in the PhyML package with default parameters (Guindon and Gascuel 2003) and 100 bootstraps, and visualized on the IToL web server (Letunic and Bork 2016). Branch-specific divergence rates were estimated with PAML 4.9 (Yang 2007), and compared with a null model presuming the same d*N*/d*S* on all branches (likelihood-ratio test, Jeffares et al. 2015).

### Transcriptome Curation and Gene Expression Analysis

#### Curation

To avoid multimapping reads resulting from having multiple transcript isoforms of the same gene in the expression analysis, we used the in-house script (Splicefinder.pl) to remove all the isoforms from the published transcriptomes of both species. In addition to that, we mapped our set of W-candidates and their homologs to their respective transcriptomes and removed any transcripts with matches >100 bp and less than 5% divergence. This is important to remove all the existing isoforms of our transcripts from the published transcriptomes, some of which are possibly hybrid assemblies between the Z and the W. Following that, we added our candidate set to the transcriptomes in order to perform gene expression analysis to untangle the differences in expression patterns between males and females.

#### Expression Analysis

For *S. japonicum*, the analysis of expression was carried out using 48 *S. japonicum* RNA-seq read libraries corresponding to three replicates of RNA-seq read data collected at eight time points of male and female *S. japonicum* development in the definitive host (Wang et al. 2017, bioproject PRJNA343582). For *S. mansoni*, the expression analysis was carried out using 28 *S. mansoni* RNA-seq libraries corresponding to two replicates of RNA-seq read data from two different studies (20 libraries from PRJNA312093; Picard et al. 2019; and 8 libraries from PRJEB1237; Lu et al. 2016; Lu et al. 2017) for different developmental stages: cercariae, three schistosomula stages, immature (unpaired), and mature (paired) adults. The transcriptomes curated specifically for the expression analysis are provided as a supplementary data 16, Supplementary Material online. The raw reads were mapped onto the transcriptome using Kallisto (v0.44.0, Bray et al. 2016), and TPM values were obtained from the Kallisto output and quantile-normalized with NormalyzerDE (Willforss et al. 2019).

### Identifying the Shared Candidates Using OrthoFinder

The identification of the orthologous genes between the two species was performed using OrthoFinder (Emms and Kelly 2019). We followed the same steps detailed in the “Transcriptome Curation and Gene Expression Analysis” section to curate the transcriptomes of the two species; however, we only included coding sequences of W-candidates and their Z-homologs in *S. japonicum* obtained in the “Estimating the Rates of Evolution of ZW Homologs” section (the two curation pipelines are outlined in supplementary fig. 15, Supplementary Material online). We downloaded the coding sequences of *Clonorchis sinensis* from the WormBase Parasite database and used it as an outgroup. As OrthoFinder takes only protein sequences as input, we used an in-house perl script “GetLongestAA_v1_July2020.pl” to perform 6-frame translation of all the transcripts and retain only the longest isoforms. Orthofinder was then run using the three sets of protein sequences to assign proteins to clusters of homologs (orthogroups). The output files “orthogroups.tsv” and “Orthogroups_UnassignedGenes.tsv” are provided in supplementary data 17, Supplementary Material online, and the transcriptomes curated specifically for OrthoFinder can be found in supplementary data 18, Supplementary Material online.

### Functional Annotation of W-Candidates

Protein sequences were extracted from the longest open reading frame of each W-candidate with “GetLongestAA_v1_July2020.pl,” and their functional annotation was performed using the web-based version of PANNZER2 (Protein ANNotation with Z-scoRE) (Törönen et al. 2018). The annotations and gene ontology predictions are provided in supplementary data 13 and 14, Supplementary Material online.

## Supplementary Material

Supplementary data are available at *Molecular Biology and Evolution* online.

## Supplementary Material

msab178_Supplementary_Data
