# Supplementary material for: Schistosome W-Linked Genes Inform Temporal Dynamics of Sex Chromosome Evolution and Suggest Candidate for Sex Determination
Source: Mol Biol Evol. 2021 Jun 19;38(12):5345–58. doi: 10.1093/molbev/msab178 (PMC8662593; doi:10.1093/molbev/msab178)
Supplement: msab178_Supplementary_Data [file msab178_supplementary_data.pdf]

SUPPLEMENTARY MATERIALS

1. Supplementary Datasets

All supplementary datasets are available at
https://seafile.ist.ac.at/d/2d9ce33a4e0c45aeadd1/

Dataset 1: Assembly of candidate W transcripts - *S. mansoni* ("original")
Dataset 2: Assembly of candidate W transcripts - *S. mansoni* ("combined", includes annotated W genes)
Dataset 3: Assembly of candidate W transcripts - *S. japonicum*
Dataset 4: ZW pairs dN/dS table
Dataset 5: Strata assignment table
Dataset 6: *S. japonicum* genome F_{ST}
Dataset 7: CNV analysis: control-FREEC outputs
Dataset 8: CNV analysis: *S. japonicum - S. mansoni* one-to-one orthologs
Dataset 9: CNV analysis: final table with gene loss
Dataset 10: ZW/ZZ dN/dS
Dataset 11: Gene expression - *S. mansoni*
Dataset 12: Gene expression - *S. japonicum*
Dataset 13: Annotation files - *S. mansoni*
Dataset 14: Annotation files - *S. japonicum*
Dataset 15: *S. japonicum* male and female transcriptomes
Dataset 16: Curated Kallisto transcriptomes
Dataset 17: Orthofinder tables
Dataset 18: Curated Orthofinder transcriptomes

2. Supplementary Figures

Supplementary Figure 1. Flow chart of the k-mer pipeline we implemented using the BBMap package.

Supplementary Figure 2. Flow chart of the steps we followed to filter the *S. mansoni* W-candidates after assembling the output of the k-mer pipeline.

Supplementary Figure 3. Flow chart of the steps we followed to filter the *S. japonicum* W-candidates after assembling the output of the k-mer pipeline.

Supplementary Figure 4. Identifying the sex of the miracidium samples used as population data to estimate FST between the male and female *S. japonicum*.

Supplementary Figure 5. Calculated FST for the different chromosomes and chromosomal regions of the sex-chromosomes in *S. japonicum*.

Supplementary Figure 6. Heatmap of the expression (TPM) of the final set of protein-coding *S. mansoni* W-candidates in males and females of different developmental stages.

Supplementary Figure 7. Heatmap of the expression (TPM) of the final set of the *S. japonicum* W-candidates in males and females.

Supplementary Figure 8. Boxplots comparing the expression (TPM) of *S. mansoni* W-candidates and their Z-homolog using an alternative RNA dataset

Supplementary Figure 9. Boxplots of the log2-transformed W-to-Z ratio of expression for *S. japonicum* for all the candidates across 8 developmental timepoints.

Supplementary Figure 10. Boxplots of the log2-transformed paired W/Z expression for *S. mansoni* for all the candidates across 5 developmental stages (study PRJNA343582)

Supplementary Figure 11. Boxplots of the log2-transformed paired W/Z expression for *S. mansoni* for all the candidates for unpaired immature adults and paired mature adults using an alternative RNA dataset (study PRJEB1237).

Supplementary Figure 12. Evolution and expression of the shared S0 gene ANKHD1 and Uev.

Supplementary Figure 13. Quality assessment of *S. japonicum* male and female transcriptome assemblies used in the process of improving the assembly of *S. japonicum* candidate W transcripts.

Supplementary Figure 14. Determination of *S. mansoni* Z-specific regions.

Supplementary Figure 15. Bioinformatic steps followed to produce the reference transcriptomes used for the Kallisto and OrthoFinder analyses.

3. Supplementary Tables

Supplementary Table 1. Publicly available DNA/RNA-seq libraries used in the different sections.

Supplementary Table 2. Publicly available assemblies used in the study.

Supplementary Table 3. Number of CNVs (loss) in female *S. japonicum*, depending on the genomic location.

Supplementary Table 4. Expression P-values (Kruskal-Wallis rank sum test) for comparisons of the strata specific expression of *S. mansoni* (using the z-homologs) (study PRJNA343582).

Supplementary Table 5. Expression P-values (Kruskal-Wallis rank sum test) for comparisons of the strata specific expression *S. japonicum* (using the z-homologs) (study PRJNA312093).

Supplementary Table 6. Expression P-values (Kruskal-Wallis rank sum test) for comparisons of the strata specific expression of *S. mansoni* (using the z-homologs) (study PRJEB1237).

Supplementary Table 7. *De novo* evolutionary strata coordinates.

4. Supplementary Code

Supplementary Code 1: Generic k-mer pipeline to detect and assemble W-transcripts from male and female DNA and RNA sequencing data

Supplementary Code 2: *S. mansoni* k-mer based identification and assembly of candidate W-derived transcripts

Supplementary Code 3: *S. japonicum* k-mer based identification and assembly of candidate W-derived transcripts

Supplementary Code 4: Strata Identification

Supplementary Code 5: A description of the process we followed to perform the FST analysis, from identifying the sex of the Miracidia samples to calculating FST

Supplementary Code 6: CNV analysis with control-FREEC

Supplementary Code 7: Estimating the Rates of Evolution of ZW homologs in *S. mansoni*

Supplementary Code 8: Estimating the Rates of Evolution of ZW homologs in *S. japonicum*

Supplementary Code 9: *S. mansoni* Transcriptome Curation for OrthoFinder

Supplementary Code 10: *S. japonicum* Transcriptome Curation for OrthoFinder

Supplementary Code 11: Running OrthoFinder

Supplementary Code 12: *S. mansoni* transcriptome curation for Kallisto

Supplementary Code 13: *S. japonicum* transcriptome curation for Kallisto

Supplementary Code 14: Expression analysis with Kallisto

2. Supplementary Figures

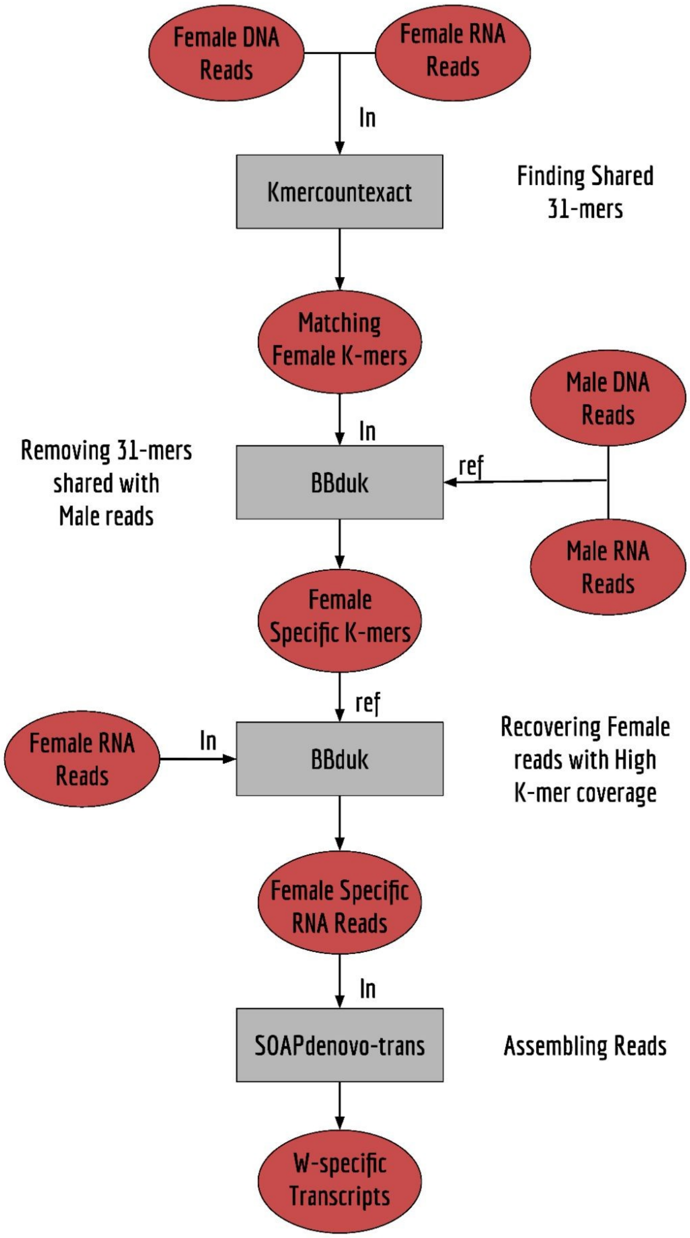

Supplementary Figure 1. Flow chart of the k-mer pipeline we implemented using the BBMap package. It has been similarly applied in both species.

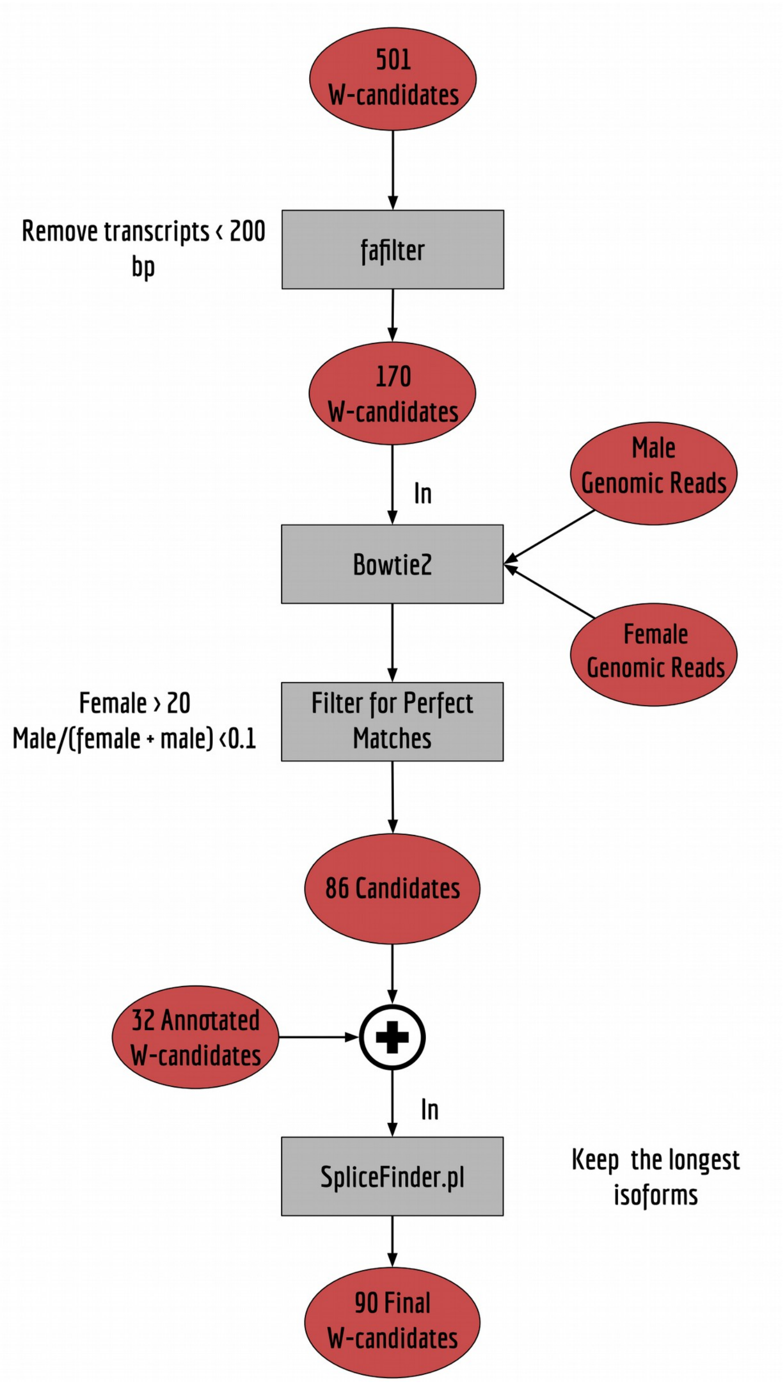

Supplementary Figure 2. Flow chart of the steps we followed to filter the *S. mansoni* W-candidates after assembling the output of the k-mer pipeline.

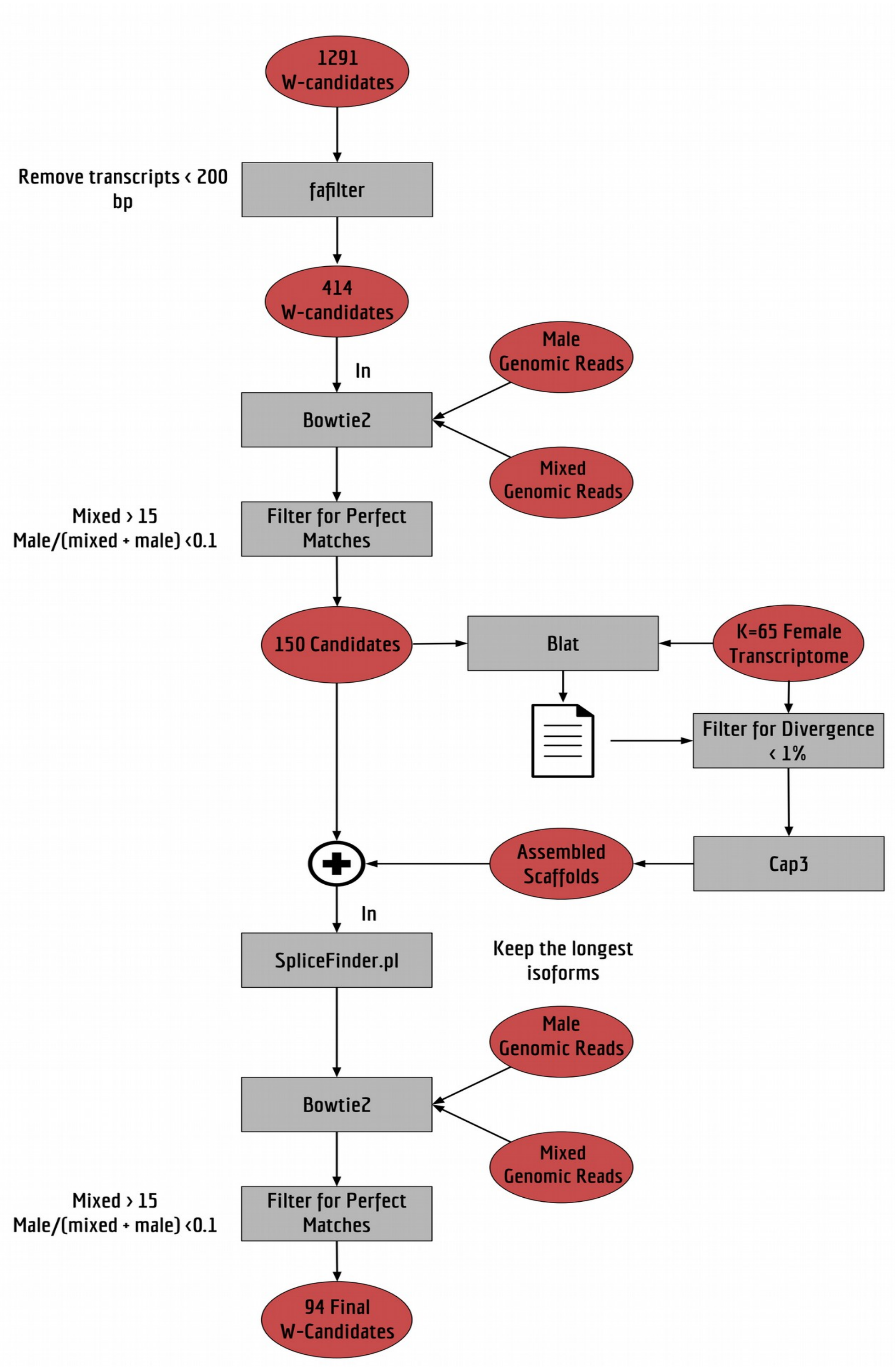

Supplementary Figure 3. Flow chart of the steps we followed to filter the *S. japonicum* W-candidates after assembling the output of the k-mer pipeline.

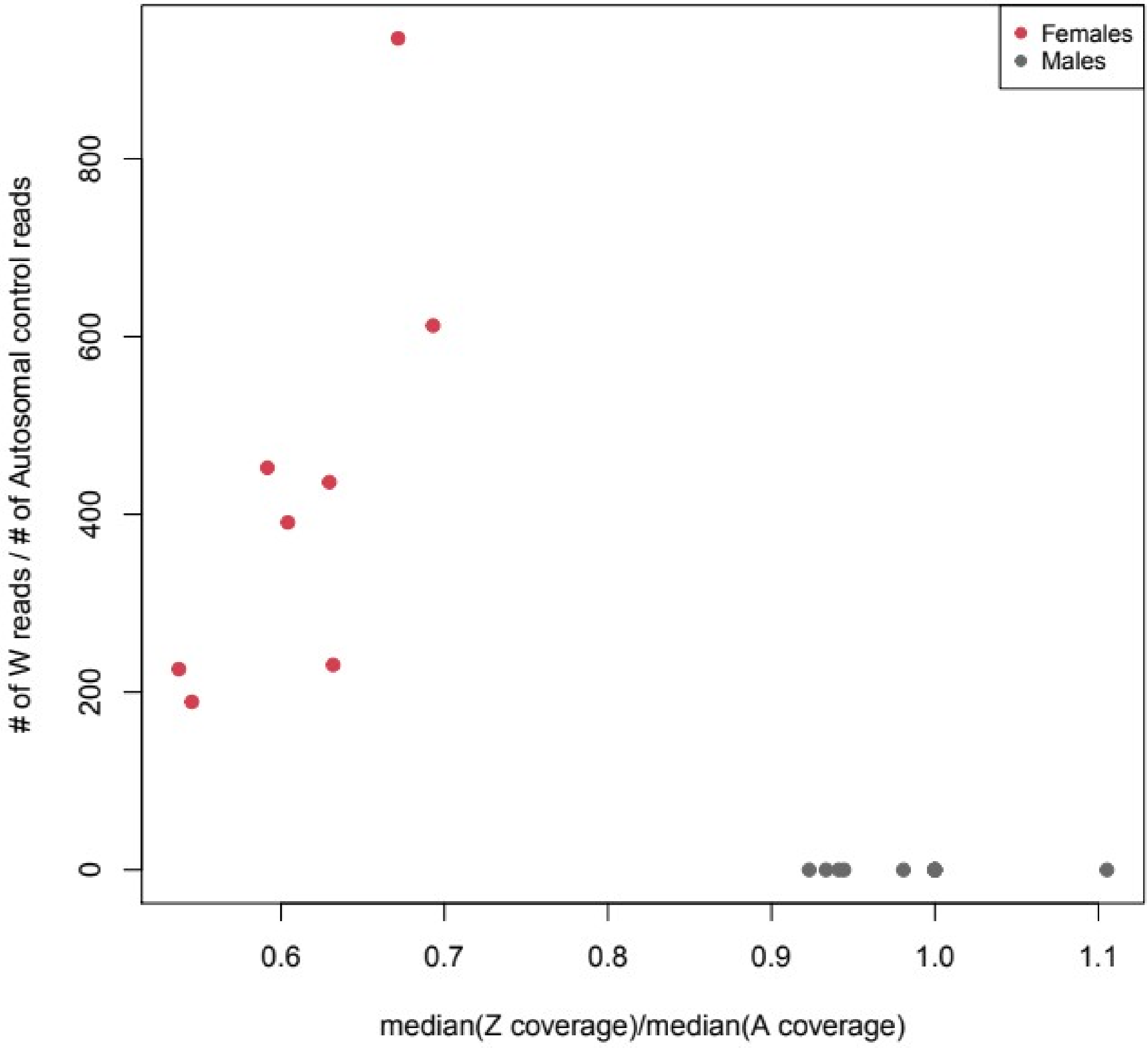

Supplementary Figure 4. Identifying the sex of the miracidium samples used as population data to estimate F_{ST} between the male and female *S. japonicum*. The x-axis shows the ratio of the median Z genomic coverage to the median autosomal "A" coverage (expected to be distributed around 1 for males and 0.5 for females). The y-axis represents the ratio of the number of reads that mapped perfectly to W-candidates (expecting many reads in females, but no reads in males) to the number that mapped to 3 autosomal controls (expecting the same number of reads in both sexes).

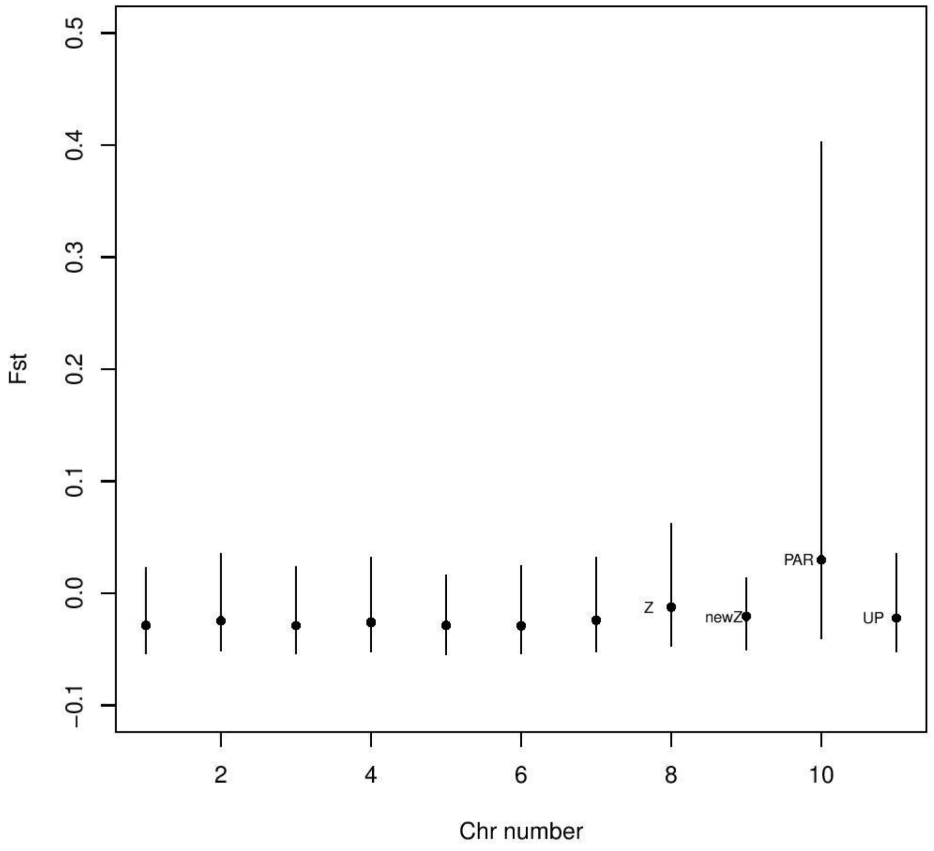

Supplementary Figure 5. Calculated F_{ST} between males and females, for the different chromosomes and chromosomal regions of the sex-chromosomes in *S. japonicum*. The plot shows high F_{ST} in the pseudoautosomal region (PAR), which is consistent with a young stratum. The different regions of the sex chromosomes correspond to assignment previsously published in Picard *et al.* 2018: Z and newZ stand respectively for previously known and newly identified Z-specific regions, UP corresponds to unplaced scaffolds.

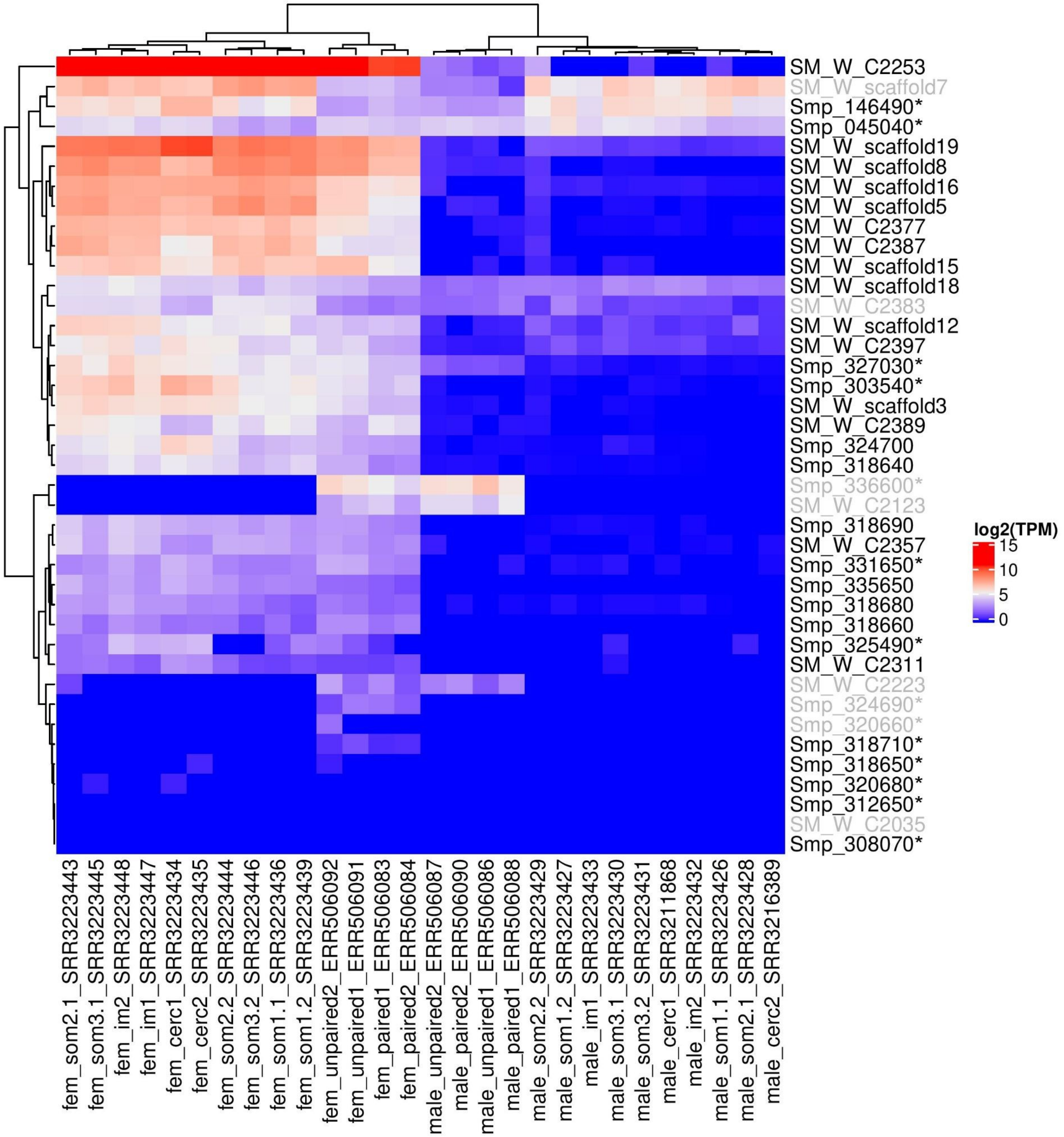

Supplementary Figure 6. Heatmap of the expression (TPM) of the final set of protein-coding *S. mansoni* W-candidates in males and females of different developmental stages. The candidates in grey did not have values in the dN/dS table (suggesting that they were short and/or extremely similar to their Z-homolog, making inferences of W-specific expression difficult), and the candidates with an asterisk are the annotated candidates which were not identified by the k-mer pipeline. Only transcripts longer than 200 bp are included in this figure.

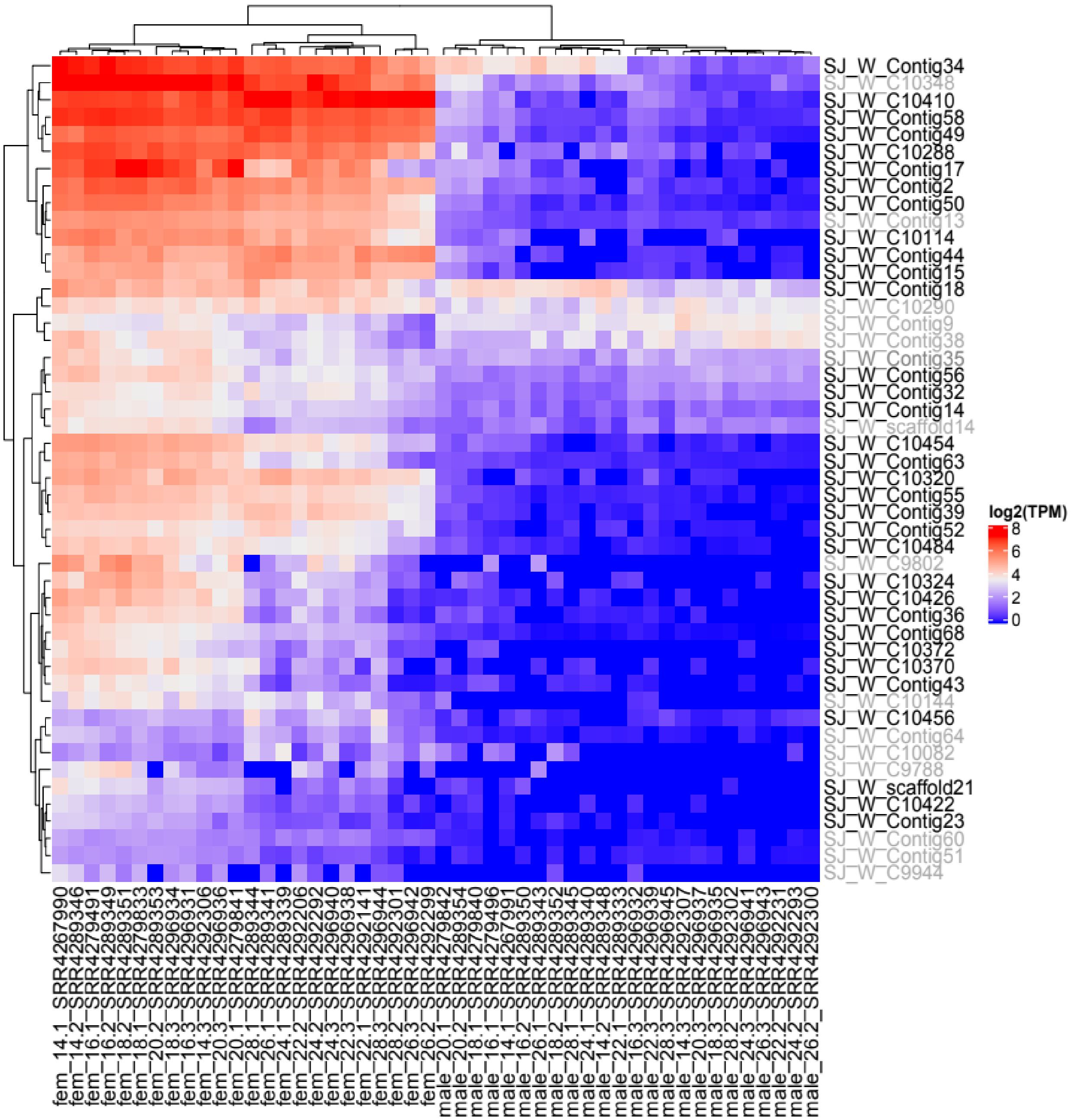

Supplementary Figure 7. Heatmap of the expression (TPM) of the final set of the *S. japonicum* W-candidates in males and females. The candidates in grey did not have values in the dN/dS table (suggesting that they were short and/or extremely similar to their Z-homolog, making inferences of W-specific expression difficult). Only transcripts longer than 200 bp are included in this figure.

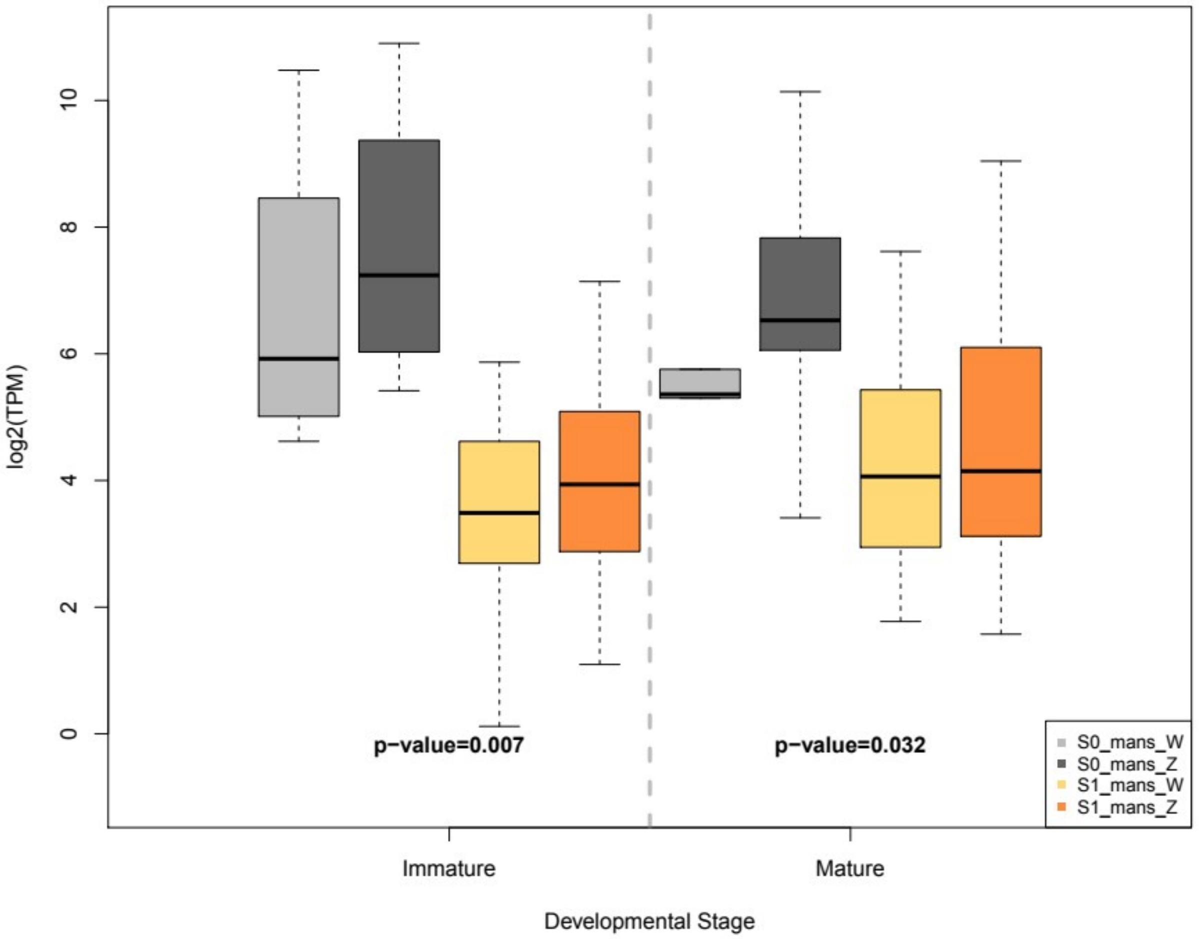

Supplementary Figure 8. Boxplots comparing the expression (TPM) of *S. mansoni* W-candidates and their Z-homolog using an alternative RNA dataset TPM values were estimated from RNA-seq data for unpaired immature adults and paired mature adults (study PRJEB1237). Stratum S0 is represented in grey and S1man in orange. P-values denote the significance of the difference in expression between W- and Z-derived transcripts, considering both strata together (Wilcoxon test).

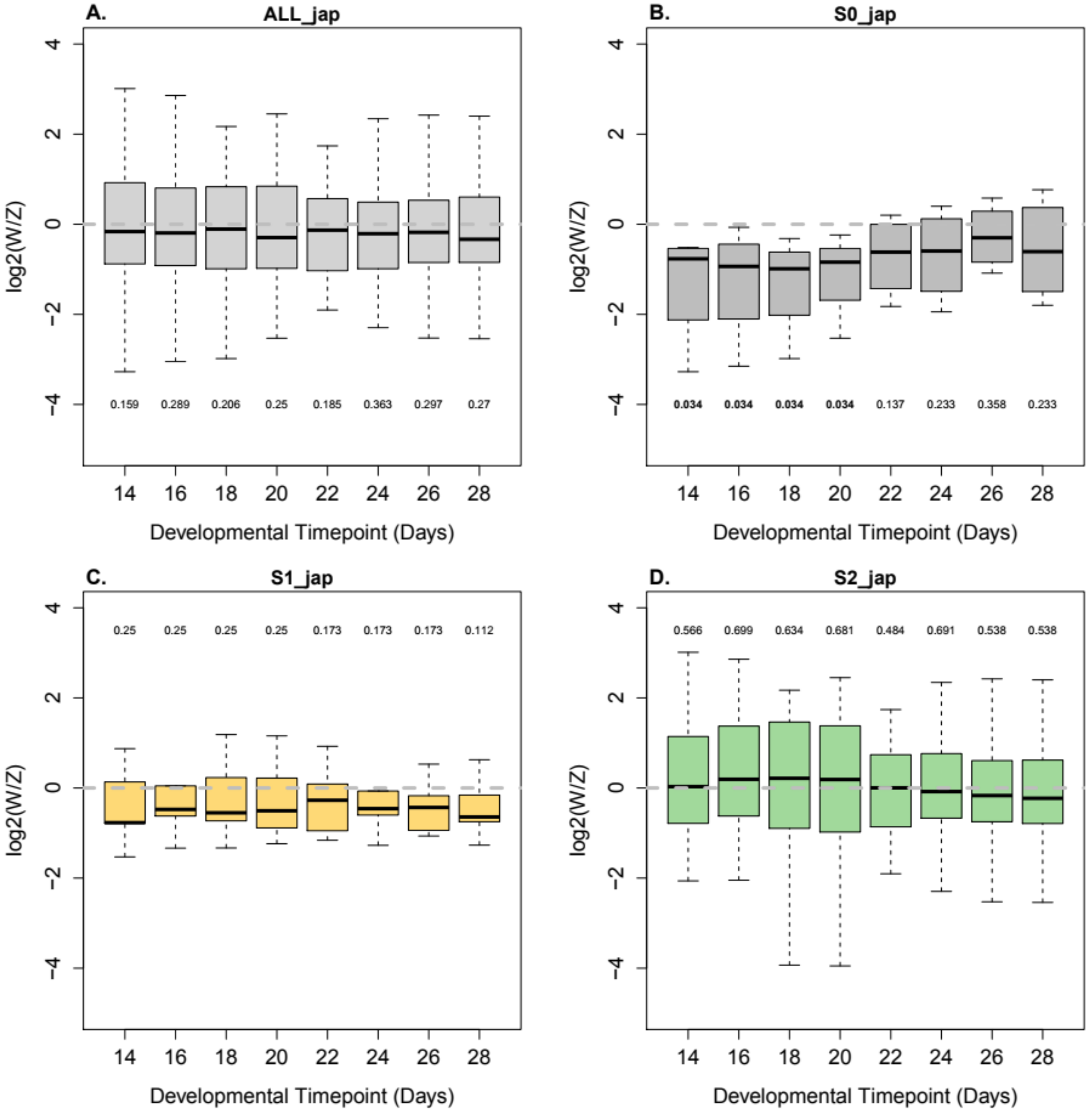

Supplementary Figure 9. Boxplots of the log2-transformed W-to-Z ratio of expression for *S. japonicum* for all the candidates across 8 developmental timepoints (study PRJNA312093): all strata together (A, light grey), stratum S0jap (B, dark grey), stratum S1jap (C, yellow), stratum S2jap (D, green). P-values on the plot show the significance of differences between W and Z (Wilcoxon test).

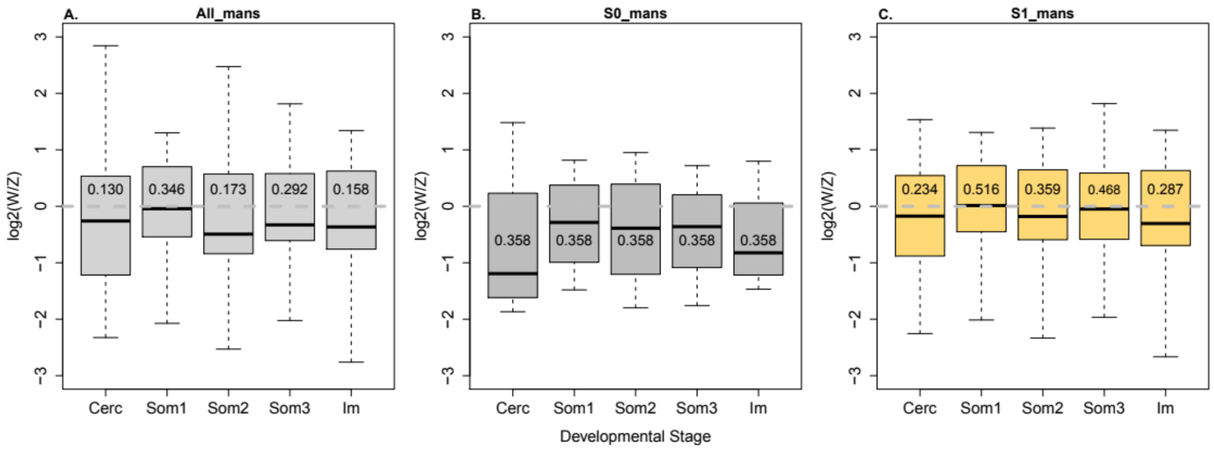

Supplementary Figure 10. Boxplots of the log2-transformed paired W/Z expression for *S. mansoni* for all the candidates across 5 developmental stages (study PRJNA343582): all strata together (A, light grey), stratum S0man (B, dark grey), stratum S1man (C, yellow). P-values on the plot show the significance of differences between W and Z (Wilcoxon test).

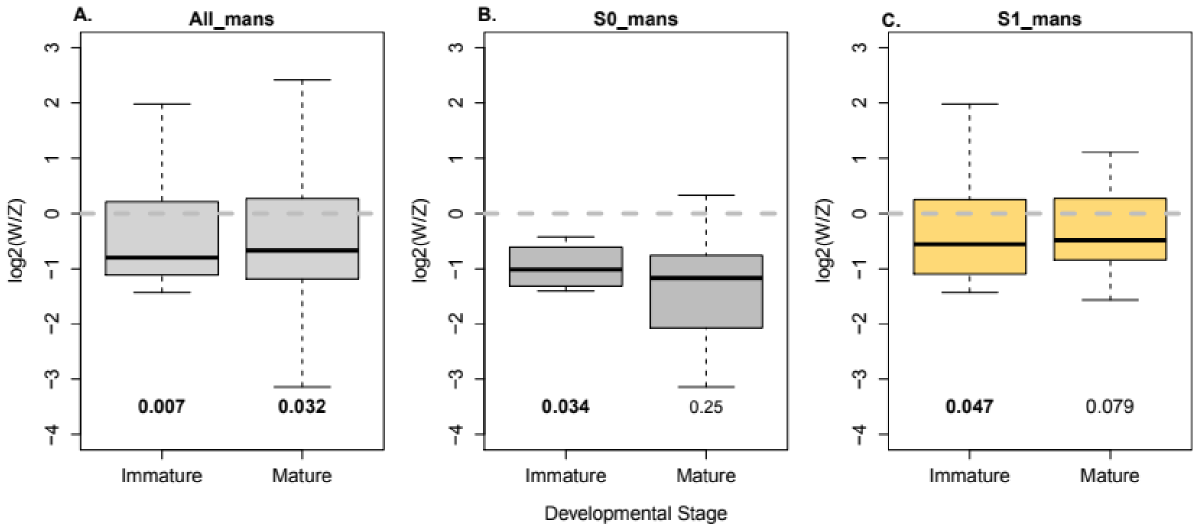

Supplementary Figure 11. Boxplots of the log2-transformed paired W/Z expression for *S. mansoni* using an alternative RNA dataset for all the candidates for unpaired immature adults and paired mature adults (study PRJEB1237): all strata together (A, light grey), stratum S0man (B, dark grey), stratum S1man (C, yellow). P-values on the plot show the significance of differences between W and Z (Wilcoxon test).

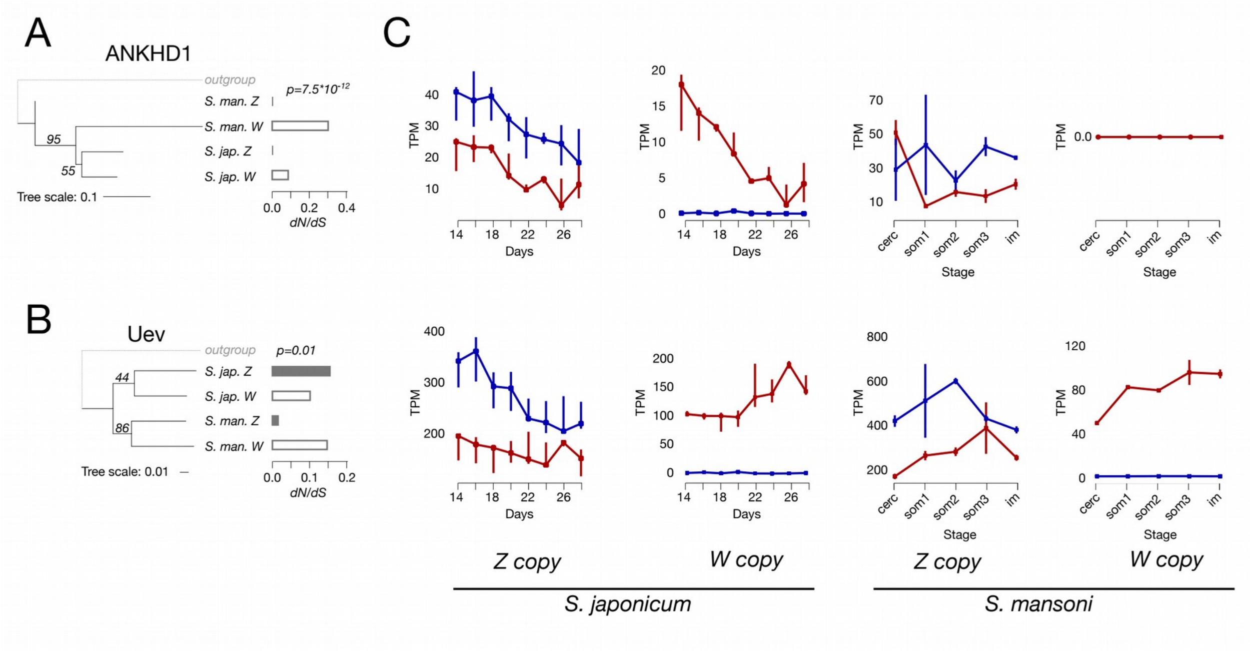

Supplementary Figure 12: Evolution and expression of the shared S0 genes ANKHD1 and Uev. Panels A and B show gene trees with bootstrap values, respectively for ANKHD1 and UeV. Terminal-branch specific dN/dS values along with the Chi-squared p-values of the deviations of observed values from the uniform assumption are shown as histograms. White bars portray dN/dS of W-specific genes, grey bars show dN/dS values of the Z-copies. Panel C shows gene expression values (TPM) of Z- and W-copies of the two S0 genes on different developmental stages of *S. japonicum* and *S. mansoni*. For *S. japonicum*, the spread between the lowest and the highest value among the three replicates is shown with error-bars, medians are shown with dots. Stages in *S. mansoni*: “cerc” means cercariae, “som1-3” are three subsequent schistosomula stages and “im” stands for immature adults. Red and blue lines show TPM values of females and males, respectively. The panels A and B and the respective genes correspond to lines of plots on the panel C.

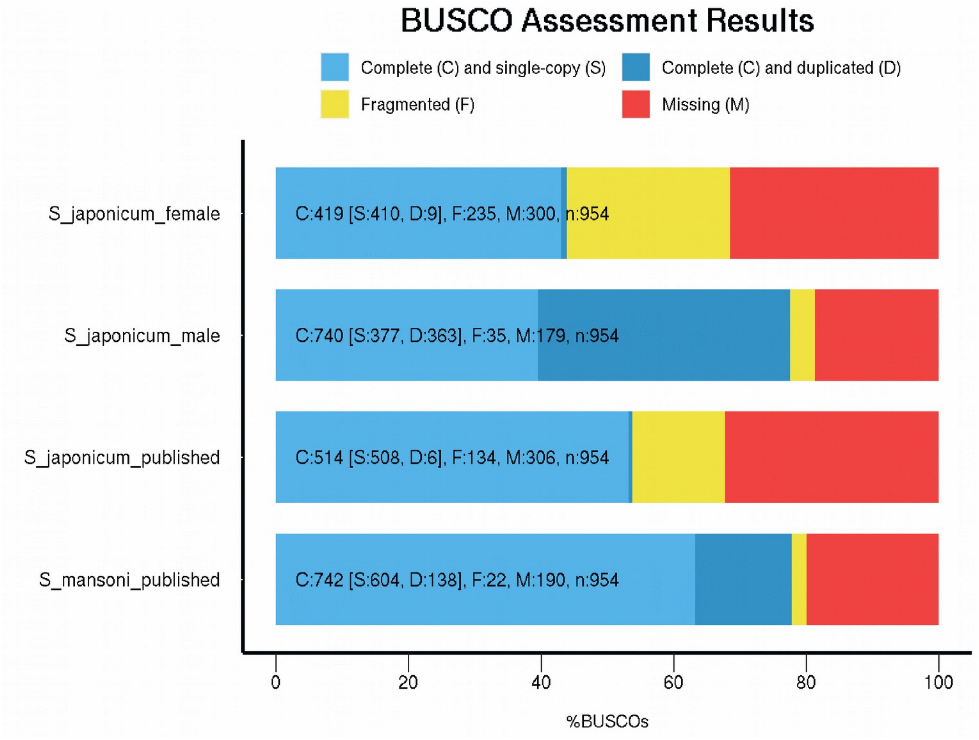

Supplementary Figure 13. Quality assessment of *S. japonicum* male and female *de novo* transcriptome assemblies used in the process of improving the assembly of *S. japonicum* candidate W transcripts. The BUSCO scores for the two assemblies are shown along with *S. japonicum* and *S. mansoni* published transcriptomes.

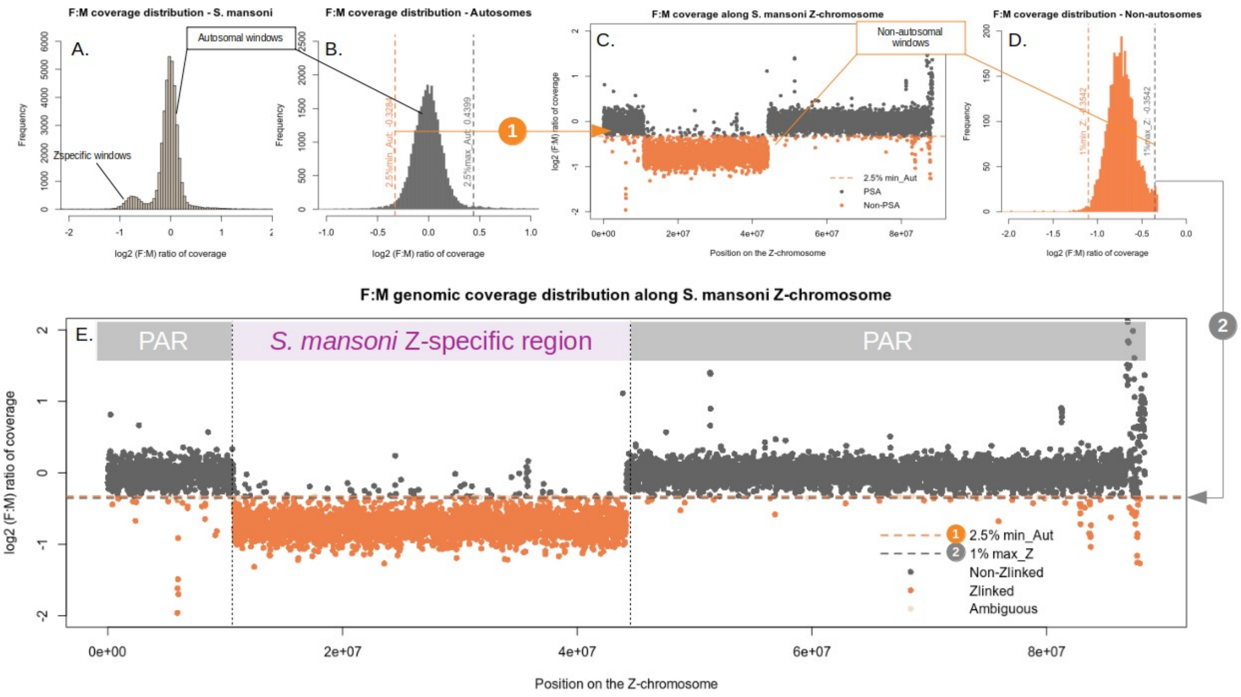

Supplementary Figure 14. Determination of *S. mansoni* Z-specific regions. The distribution of the female-to-male (F:M) coverage ratio is plotted in A (log2 scale) and displays a bimodal distribution. The highest peak, around 0, corresponds to the autosomal windows that are plotted alone in B. We determined a lower threshold of -0.3284 (orange disk#1, corresponding to the 2.5th percentile of the autosome distribution), which defines the lowest log2(F:M) value for autosomal windows (orange dashed line in B and C). We then extracted non-autosomal windows (in orange) and plotted their distribution in D. The value corresponding to the 99th percentile of this unimodal distribution was then used as the upper limit coverage of Z-specific regions in E (*i.e.* log2(F:M)=-0.3542, grey disk#2). In E, the few ambiguous windows (plotted in beige), are windows with a log2(F:M) between the defined upper limit of Z-specific regions (grey dashed line) and the lower limit of autosomal regions (orange dashed line). Purple and grey rectangles respectively represent the final *S. mansoni* Z-specific and pseudoautosomal (PAR) regions, plotted along the Z.

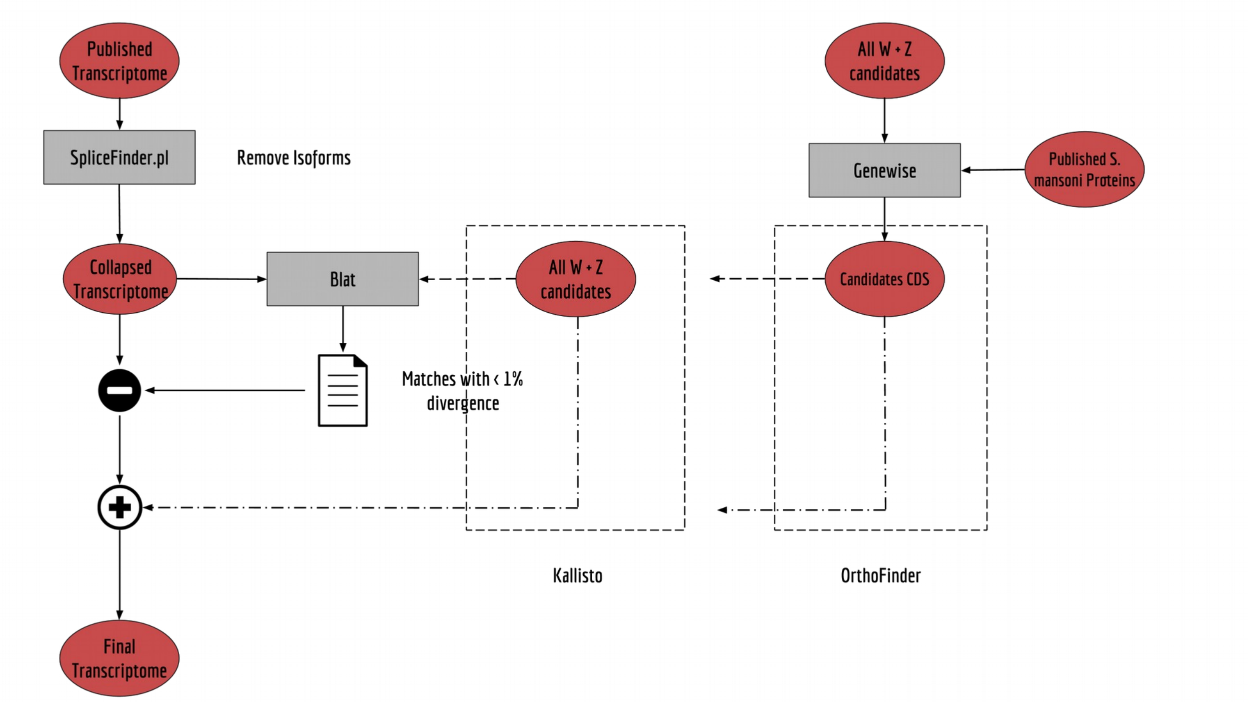

Supplementary Figure 15. Bioinformatic steps followed to produce the reference transcriptomes used for the Kallisto and OrthoFinder analyses. It has been similarly applied in both species.

3. Supplementary Tables

Supplementary Table 1. Publicly available DNA/RNA-seq libraries used in the different sections.

Section	BioProject/ SRA Study	Run	Sex	Number of individuals	Developmental stage	Strategy
**4.2 k-mer based assembly and filtering of *S. mansoni* W-linked genes**	PRJEB2320/<br>ERP000385	ERR562989<br>ERR562990	Male<br>Female	6000 ind. (uniclonal) †<br>6000 ind. (uniclonal)	cercariae<br>cercariae	WGS
	PRJNA312093/<br>SRP071285	SRR3223434<br>SRR3223435<br>SRR3223443<br>SRR3223444<br>SRR3223447<br>SRR3223448<br><br>SRR3211868<br>SRR3216389<br>SRR3223428<br>SRR3223429<br>SRR3223432*<br>SRR3223433	Female<br>Female<br>Female<br>Female<br>Female<br>Female<br><br>Male<br>Male<br>Male<br>Male<br>Male<br>Male	Thousands (multiclonal) ††<br>Thousands (multiclonal)<br>Hundreds (multiclonal)<br>Hundreds (multiclonal)<br>Dozens (multiclonal)<br>Dozens (multiclonal)<br><br>Thousands (multiclonal)<br>Thousands (multiclonal)<br>Hundreds (multiclonal)<br>Hundreds (multiclonal)<br>Dozens (multiclonal)<br>Dozens (multiclonal)	cercariae<br>cercariae<br>schistosomula2<br>schistosomula2<br>immature adult<br>immature adult<br><br>cercariae<br>cercariae<br>schistosomula2<br>schistosomula2<br>immature adult<br>immature adult	RNA-seq
	PRJEB1237/<br>ERP002073	ERR506083<br>ERR506084<br>ERR506088<br>ERR506090	Female<br>Female<br>Male<br>Male	1 individual †<br>1 ind.<br>1 ind.<br>1 ind.	mature adult<br>mature adult<br>mature adult<br>mature adult	RNA-seq
**4.2 k-mer based assembly of *S. japonicum* W-linked genes**	PRJNA432803/<br>SRP135770	SRR6841388<br>SRR6841389	Female<br>Male	33 ind. (multiclonal) †††<br>28 ind. (multiclonal)	mature adult<br>mature adult	WGS
	PRJNA343582/<br>SRP090154	SRR4267990<br>SRR4279491<br>SRR4279496<br>SRR4267991	Female<br>Female<br>Male<br>Male	800-1000 (multiclonal) †††<br>600-800 (multiclonal)<br>600-800 (multiclonal)<br>800-1000 (multiclonal)	14days<br>16days<br>16days<br>14days	RNA-seq
	PRJNA252904/<br>SRP043313	SRR1421523<br>SRR1421524	Female<br>Male	1 ind. †<br>1 ind.	mature adult<br>mature adult	RNA-seq
**4.2 Filtering the *S. japonicum* candidates**	PRJNA354903/<br>SRP093905	SRR5054524<br>SRR5054649<br>SRR5054671<br>SRR5054674<br>SRR5054672<br>SRR5054673<br>SRR5054701	Male<br>Male<br>Male<br>Male<br>Mixed<br>Mixed<br>Mixed	120 (unknown) †<br>150 (unknown)<br>160 (unknown)<br>90 (unknown)<br>60 (multiclonal)<br>100 (multiclonal)<br>150 (multiclonal)	mature adult<br>mature adult<br>mature adult<br>mature adult<br>mature adult<br>mature adult<br>mature adult	WGS
**4.2 Assembly of *S. japonicum* female transcriptome**	PRJNA343582/<br>SRP090154	SRR4292206<br>SRR4292292<br>SRR4292299<br>SRR4292301<br>SRR4292306<br>SRR4296931<br>SRR4296934<br>SRR4296936<br>SRR4296938<br>SRR4296940	Female<br>Female<br>Female<br>Female<br>Female<br>Female<br>Female<br>Female<br>Female<br>Female	150-200 (multiclonal) †††<br>100-150 (multiclonal)<br>80-100 (multiclonal)<br>80-100 (multiclonal)<br>800-1000 (multiclonal)<br>600-800 (multiclonal)<br>500-600 (multiclonal)<br>200-250 (multiclonal)<br>150-200 (multiclonal)<br>100-150 (multiclonal)	22days<br>24days<br>26days<br>28days<br>14days<br>16days<br>18days<br>20days<br>22days<br>24days	RNA-seq

		SRR4296942<br>SRR4296944	Female<br>Female	80-100 (multiclonal)<br>80-100 (multiclonal)	26days<br>28days	
**4.2 Assembly of *S. japonicum* male transcriptome**	PRJNA504625/<br>SRP168226	SRR8175618	Male	20 (multiclonal) †††	adult	RNA-seq
**4.4 Z-specific regions in *S. mansoni***	PRJEB2320/<br>ERP000385	ERR562989<br>ERR562990	Male<br>Female	6000 (uniclonal) †<br>6000 (uniclonal)	cercariae<br>cercariae	WGS
**4.5 F_{ST} analysis *S. japonicum***	PRJNA650045/<br>SRP274938	SRR12363890<br>SRR12363891<br>SRR12363892<br>SRR12363893<br>SRR12363894<br>SRR12363896<br>SRR12363898<br>SRR12363899<br>SRR12363900<br>SRR12363901<br>SRR12363902<br>SRR12363903<br>SRR12363904<br>SRR12363905<br>SRR12363907<br>SRR12363908<br>SRR12363909<br>SRR12363910<br>SRR12363911	Male<br>Male<br>Male<br>Male<br>Female<br>Male<br>Female<br>Male<br>Female<br>Female<br>Male<br>Male<br>Female<br>Male<br>Male<br>Female<br>Female<br>Female<br>Male	1 individual †<br>1 ind.<br>1 ind.<br>1 ind.<br>1 ind.<br>1 ind.<br>1 ind.<br>1 ind.<br>1 ind.<br>1 ind.<br>1 ind.<br>1 ind.<br>1 ind.<br>1 ind.<br>1 ind.<br>1 ind.<br>1 ind.<br>1 ind.<br>1 ind.	Miracidia<br>Miracidia<br>Miracidia<br>Miracidia<br>Miracidia<br>Miracidia<br>Miracidia<br>Miracidia<br>Miracidia<br>Miracidia<br>Miracidia<br>Miracidia<br>Miracidia<br>Miracidia<br>Miracidia<br>Miracidia<br>Miracidia<br>Miracidia<br>Miracidia	WGS
**4.6 CNV analysis in *S. japonicum***	PRJNA432803/<br>SRP135770	SRR6841388<br>SRR6841389	Female<br>Male	33 ind. (multiclonal) †††<br>28 ind. (multiclonal)	mature adult<br>mature adult	WGS
**4.8 Gene expression Analysis *S. mansoni***	PRJNA312093/<br>SRP071285	SRR3211868<br>SRR3216389<br>SRR3223426<br>SRR3223427<br>SRR3223428<br>SRR3223429<br>SRR3223430<br>SRR3223431<br>SRR3223433<br>SRR3223432*<br><br>SRR3223434<br>SRR3223435<br>SRR3223436<br>SRR3223439<br>SRR3223443<br>SRR3223444<br>SRR3223445<br>SRR3223446<br>SRR3223447<br>SRR3223448	Male<br>Male<br>Male<br>Male<br>Male<br>Male<br>Male<br>Male<br>Male<br>Male<br><br>Female<br>Female<br>Female<br>Female<br>Female<br>Female<br>Female<br>Female<br>Female<br>Female	Thousands (multiclonal) ††<br>Thousands (multiclonal)<br>Hundreds (multiclonal)<br>Hundreds (multiclonal)<br>Hundreds (multiclonal)<br>Hundreds (multiclonal)<br>Hundreds (multiclonal)<br>Hundreds (multiclonal)<br>Dozens (multiclonal)<br>Dozens (multiclonal)<br><br>Thousands (multiclonal)<br>Thousands (multiclonal)<br>Hundreds (multiclonal)<br>Hundreds (multiclonal)<br>Hundreds (multiclonal)<br>Hundreds (multiclonal)<br>Hundreds (multiclonal)<br>Hundreds (multiclonal)<br>Dozens (multiclonal)<br>Dozens (multiclonal)	cercariae<br>cercariae<br>schistosomula1<br>schistosomula1<br>schistosomula2<br>schistosomula2<br>schistosomula3<br>schistosomula3<br>immature adult<br>immature adult<br><br>cercariae<br>cercariae<br>schistosomula1<br>schistosomula1<br>schistosomula2<br>schistosomula2<br>schistosomula3<br>schistosomula3<br>immature adult<br>immature adult	RNA-seq
	PRJEB1237/<br>ERP002073	ERR506083<br>ERR506084	Female<br>Female	1 individual †<br>1 ind.	mature adult<br>mature adult	RNA-seq

		ERR506091<br>ERR506092<br><br>ERR506088<br>ERR506090<br>ERR506086<br>ERR506087	Female<br>Female<br><br>Male<br>Male<br>Male<br>Male	1 ind.<br>1 ind.<br><br>1 ind.<br>1 ind.<br>1 ind.<br>1 ind.	immature adult<br>immature adult<br><br>mature adult<br>mature adult<br>immature adult<br>immature adult	
**4.8Gene expression analysis *S. japonicum***	PRJNA343582/ SRP090154	SRR4267990<br>SRR4289346<br>SRR4292306<br>SRR4279491<br>SRR4289349<br>SRR4296931<br>SRR4279833<br>SRR4289351<br>SRR4296934<br>SRR4279841<br>SRR4289353<br>SRR4296936<br>SRR4292141<br>SRR4292206<br>SRR4296938<br>SRR4289339<br>SRR4292292<br>SRR4296940<br>SRR4289341<br>SRR4292299<br>SRR4296942<br>SRR4289344<br>SRR4292301<br>SRR4296944<br><br>SRR4267991<br>SRR4289348<br>SRR4292307<br>SRR4279496<br>SRR4289350<br>SRR4296932<br>SRR4279840<br>SRR4289352<br>SRR4296935<br>SRR4279842<br>SRR4289354<br>SRR4296937<br>SRR4289333<br>SRR4292231<br>SRR4296939<br>SRR4289340<br>SRR4292293<br>SRR4296941<br>SRR4289343<br>SRR4292300<br>SRR4296943<br>SRR4289345<br>SRR4292302<br>SRR4296945	Female<br><br>Male<br>Male<br>Male<br>Male<br>Male<br>Male<br>Male<br>Male<br>Male<br>Male<br>Male<br>Male<br>Male<br>Male<br>Male<br>Male<br>Male<br>Male<br>Male<br>Male<br>Male<br>Male<br>Male<br>Male	800-1000 (multiclonal) †††<br>800-1000 (multiclonal)<br>800-1000 (multiclonal)<br>600-800 (multiclonal)<br>600-800 (multiclonal)<br>600-800 (multiclonal)<br>500-600 (multiclonal)<br>500-600 (multiclonal)<br>500-600 (multiclonal)<br>200-250 (multiclonal)<br>200-250 (multiclonal)<br>200-250 (multiclonal)<br>150-200 (multiclonal)<br>150-200 (multiclonal)<br>150-200 (multiclonal)<br>100-150 (multiclonal)<br>100-150 (multiclonal)<br>100-150 (multiclonal)<br>80-100 (multiclonal)<br>80-100 (multiclonal)<br>80-100 (multiclonal)<br>80-100 (multiclonal)<br>80-100 (multiclonal)<br>80-100 (multiclonal)<br><br>800-1000 (multiclonal)<br>800-1000 (multiclonal)<br>800-1000 (multiclonal)<br>600-800 (multiclonal)<br>600-800 (multiclonal)<br>600-800 (multiclonal)<br>500-600 (multiclonal)<br>500-600 (multiclonal)<br>500-600 (multiclonal)<br>200-250 (multiclonal)<br>200-250 (multiclonal)<br>200-250 (multiclonal)<br>150-200 (multiclonal)<br>150-200 (multiclonal)<br>150-200 (multiclonal)<br>100-150 (multiclonal)<br>100-150 (multiclonal)<br>100-150 (multiclonal)<br>80-100 (multiclonal)<br>80-100 (multiclonal)<br>80-100 (multiclonal)<br>80-100 (multiclonal)<br>80-100 (multiclonal)<br>80-100 (multiclonal)	14days<br>14days<br>14days<br>16days<br>16days<br>16days<br>18days<br>18days<br>18days<br>20days<br>20days<br>20days<br>22days<br>22days<br>22days<br>24days<br>24days<br>24days<br>26days<br>26days<br>26days<br>28days<br>28days<br>28days<br><br>14days<br>14days<br>14days<br>16days<br>16days<br>16days<br>18days<br>18days<br>18days<br>20days<br>20days<br>20days<br>22days<br>22days<br>22days<br>24days<br>24days<br>24days<br>26days<br>26days<br>26days<br>28days<br>28days<br>28days	RNA-seq

**The wrong file was uploaded to the SRA repository, so we requested the original file from the authors † Source: NCBI-SRA database; †† Source: authors (Picard et al., PlosNTD, 2016); ††† Source: publication (PRJNA432803: Picard et al., elife, 2018; PRJNA343582: Wang et al. Nat. Com., 2017; PRJNA504625: Rong et al., Frontiers in microbiology, 2020)*

Supplementary Table 2. Publicly available assemblies used in the study.

Reference Assemblies	Source	Species	Release	BioProject	Downloaded On
CDS_transcripts	WormBase Parasite	*S. japonicum*	WBPS14	PRJEA34885	06-08-2020
		S. mansoni	WBPS14	PRJEA36577	31-07-2020
		C. sinensis	WBPS14	PRJNA386618	11-09-2020
Genomic		*S. japonicum*	WBPS9	PRJEA34885	29-09-2017
		S. mansoni	WBPS14	PRJEA36577	30-10-2020
Protein		*S. mansoni*	WBPS14	PRJEA36577	09-11-2020
Canonical Geneset		*S. mansoni*	WBPS14	PRJEA36577	23-07-2020

Supplementary Table 3: Number of CNVs (loss) in female *S. japonicum*, depending on the genomic location. CNV analyses were performed on windows of 1kb, 5kb and 10kb and the reported numbers correspond to all three analyses taken together. Only significant losses are shown (wilcoxon p-value < 0.05). Genomic location of *S. japonicum* genes was inferred thanks to one-to-one orthologs of *S. mansoni*.

**Genomic location**		**Total number of *S. mansoni* genes**	**Deleted copies (CNVs)**	**Proportion of deletion**
SM_V7_ZW	S0	635	44	6,93 % *
	S1jap	143	8	5,59 % *
	S1mans	299	3	1,00 %
	S2jap	720	7	0,97 %
SM_V7_1		2371	21	0,89 %
SM_V7_2		1243	12	0,97 %
SM_V7_3		1244	11	0,88 %
SM_V7_4		1176	10	0,85 %
SM_V7_5		579	14	2,42 %
SM_V7_6		758	6	0,79 %
SM_V7_7		416	5	1,20 %
n.a. (no *S. mansoni* ortholog)		-	13	-

***These proportions are not representative of the true amount of gene loss in these ancient strata, as CONTROL-FREEC controls for background (scaffold) differences in coverage, thereby losing much of the signal when the whole sequence is missing.**

Supplementary Table 4. Expression P-values (Kruskal-Wallis rank sum test) for comparisons of the strata specific expression of *S. mansoni* (using the Z-homologs) (study PRJNA343582).

Stage	P-value
Cercariae	**0.012**
Schistosomula 1	0.053
Schistosomula 2	0.053
Schistosomula 3	**0.041**
Immature adults	**0.032**

Supplementary Table 5. Expression P-values (Kruskal-Wallis rank sum test) for comparisons of the strata specific expression *S. japonicum* (using the Z-homologs) (study PRJNA312093).

Time-point	P-value
14 days	**0.038**
16 days	**0.038**
18 days	**0.039**
20 days	**0.037**
22 days	**0.047**
24 days	**0.04**
26 days	0.051
28 days	**0.032**

Supplementary Table 6. Expression P-values (Kruskal-Wallis rank sum test) for comparisons of the strata specific expression of *S. mansoni* (using the Z-homologs) using an alternative RNA dataset (study PRJEB1237).

Stage	P-value
Immature unpaired adults	0.053
Mature paired adults	**0.047**

Supplementary Table 7: *De novo* evolutionary strata coordinates. Coordinates were defined based on the *S. mansoni* reference genome v7 (GCA_000237925.3 assembly): a stratum starts at the first base of the first CDS that it contains.

**Strata/PAR on the Z**	**Start (bases)**	**Stop (bases)**
S0	21,065,055	21,940,030
	23,467,694	44,205,956
S1man	10,740,979	21,065,054
	21,940,031	23,467,693
S1jap	44,205,957	45,023,775
	46,909,733	51,501,679
S2jap	45,023,776	46,909,732
	51,501,680	78,402,349
Shared PAR	0	10,740,978
	78,402,350	end

4. Supplementary Codes

All supplementary codes are available on: https://github.com/Melkrewi/Schisto_project

Supplementary Code 1: Generic k-mer pipeline to detect and assemble W-transcripts from male and female DNA and RNA sequencing data.

*kmercountexact.sh and bbduk.sh scripts mentioned below are part of the BBMap package.

```
module load java
module load bbmap

kmercountexact.sh k=31 in1=female_dna_forward_reads.fq in2=female_dna_reverse_reads.fq
out=female_dna_mers.fa #k-mer counting paired DNA library

kmercountexact.sh k=31 in=pooled_rna_replicate_1.fq out=female_rna_mers_1.fa #k-mer counting
single-end RNA library

kmercountexact.sh k=31 in=pooled_rna_replicate_2.fq out=female_rna_mers_2.fa

kmercountexact.sh k=31 in=female_dna_mers.fa,female_rna_mers_1.fa,female_rna_mers_2.fa
out=shared_female_mers.fa mincount=3 #outputs shared k-mers between DNA and RNA

bbduk.sh k=31 in=shared_female_mers.fa out=female_specific_mers.fa
ref=male_dna_forward_reads.fq,male_dna_reverse_reads.fq #removes k-mers matching male dna

bbduk.sh k=31 in=female_specific_mers.fa out=v_female_specific_mers.fa
ref=male_rna_1.fq,male_rna_2.fq,male_rna_3.fq,... #removes k-mers matching male RNA libraries

bbduk.sh k=31 in=pooled_rna_replicate_2.fq outm=female_specific_rna_reads_1.fastq
ref=v_female_specific_mers.fa mink-merfraction=0.4

bbduk.sh k=31 in=pooled_rna_replicate_2.fq outm=female_specific_rna_reads_2.fastq
ref=v_female_specific_mers.fa mink-merfraction=0.4
```

Supplementary Code 2: *S. mansoni* k-mer based identification and assembly of candidate W-derived transcripts

2.1 Bash commands used to pool the developmental stages together

```
#csi_1.fq:
cat SRR3223434_trimmed.fq SRR3223443_trimmed.fq SRR3223447_trimmed.fq > csi_1.fq
#csi_2.fq:
cat SRR3223435_trimmed.fq SRR3223444_trimmed.fq SRR3223448_trimmed.fq > csi_2.fq

#ERR50608:
cat ERR506083_forward_paired.fq.gz ERR506084_forward_paired.fq.gz > ERR50608_1.fq.gz
cat ERR506083_reverse_paired.fq.gz ERR506084_reverse_paired.fq.gz > ERR50608_2.fq.gz

#csim_1.fq:
cat ERR506083_forward_paired.fq.gz ERR506083_reverse_paired.fq.gz > ERR506083.fq.gz
gunzip ERR506083.fq.gz
```

```
cat ERR506083.fq csi_1.fq > csim_1.fq

#csim_2.fq:
cat ERR506084_forward_paired.fq.gz ERR506084_reverse_paired.fq.gz > ERR506084.fq.gz
gunzip ERR506084.fq.gz
cat ERR506084.fq csi_2.fq > csim_2.fq
```

2.2 Running the k-mer pipeline on the pooled data

```
module load java
module load bbmap

kmer=31

echo "### Step 0: Kmer pipeline starting"
kmercountexact.sh k=$kmer in1=ERR562990_forward_paired.fq.gz
in2=ERR562990_reverse_paired.fq.gz out=female_dna_mer_1.fa

kmercountexact.sh k=$kmer in=csim_1.fq out=sfemale_rna_mer_1.fa

kmercountexact.sh k=$kmer in=csim_2.fq out=sfemale_rna_mer_2.fa

kmercountexact.sh k=$kmer in=female_dna_mer_1.fa,sfemale_rna_mer_1.fa,sfemale_rna_mer_2.fa
out=shared_female_mer.fa mincount=3

bbduk.sh k=$kmer in=shared_female_mer.fa out=female_specific_mers.fasta
ref=ERR562989_forward_paired.fq.gz,ERR562989_reverse_paired.fq.gz -Xmx350g

bbduk.sh k=$kmer in=female_specific_mers.fasta out=v_female_specific_mers.fasta
ref=ERR506088_forward_paired.fq.gz,ERR506088_reverse_paired.fq.gz,ERR506090_forward_paired.f
q.gz,ERR506090_reverse_paired.fq.gz

bbduk.sh k=$kmer in=v_female_specific_mers.fasta out=v_v_female_specific_mers.fasta
ref=SRR3211868_trimmed.fq,SRR3216389_trimmed.fq,SRR3223428_trimmed.fq,SRR3223429_trimm
ed.fq,SRR3223433_trimmed.fq,E1bis_E2bis_CTTGTA_L003_R1_concat_trimmed.fq

bbduk.sh k=$kmer in=csi_1.fq outm=female_specific_rna_reads_1.fastq
ref=v_v_female_specific_mers.fasta minkmerfraction=0.4

bbduk.sh k=$kmer in=csi_2.fq outm=female_specific_rna_reads_2.fastq
ref=v_v_female_specific_mers.fasta minkmerfraction=0.4

bbduk.sh k=$kmer in1=ERR50608_1.fq.gz in2=ERR50608_2.fq.gz
outm1=mature_adult_specific_rna_reads_1.fastq outm2=mature_adult_specific_rna_reads_2.fastq
ref=v_v_female_specific_mers.fasta minkmerfraction=0.4
```

2.3 Concatenation of the two female_specific_rna_reads files before the assembly step

```
cat female_specific_rna_reads_1.fastq female_specific_rna_reads_2.fastq >
female_specific_rna_reads.fastq
```

2.4 SOAPdenovo-trans config file used to assemble the output of the k-mer pipeline

```
#maximal read length

max_rd_len=101

[LIB]

#maximal read length in this lib

rd_len_cutof=101

#average insert size

avg_ins=100

#if sequence needs to be reversed

reverse_seq=0

#in which part(s) the reads are used

asm_flags=3

#minimum aligned length to contigs for a reliable read location (at least 32 for short insert size)

map_len=32

#fastq file for read 1

q1=mature_adult_specific_rna_reads_1.fastq

q2=mature_adult_specific_rna_reads_2.fastq

q=female_specific_rna_reads.fastq
```

2.5 Assembly using SOAPdenovo

```
echo "### Step 1: Assembly of Candidates"
module load SOAPdenovoTrans

srun SOAPdenovo-Trans-31mer all -s soapconfig.txt -K 15 -o TranscriptAssembly -p 16 1>
TranscriptAssembly.stdout 2> TranscriptAssembly.stderr
```

2.6 *faFilter* to remove transcripts < 200bp

```
echo "### Step 2: Removing candidates shorter than 200 bp"
module load fafilter
srun faFilter -minSize=200  TranscriptAssembly.scafSeq mansoni_200.fasta
```

2.7 *Bowtie2* to map the male and female genomic reads to the W candidates and count perfect matches

```
echo "### Step 3: Filtering Stage 1"
module load java
```

```
module load bowtie2/2.3.4.1

srun bowtie2-build mansoni_200.fasta transcripts_w

srun bowtie2 -p 8 --no-unal --no-hd --no-sq -x transcripts_w -U ERR562990_1.fastq -S female_1.sam

srun bowtie2 -p 8 --no-unal --no-hd --no-sq -x transcripts_w -U ERR562989_1.fastq -S male_1.sam

awk '($6=="100M")' female_1.sam | grep 'NM:i:0' > female_1_perfectmatch.sam

awk '($6=="100M")' male_1.sam | grep 'NM:i:0' > male_1_perfectmatch.sam

grep '>' mansoni_200.fasta | perl -pi -e 's/>//gi' | perl -pi -e 's/ .*//gi' > transcripts.list

cat female_1_perfectmatch.sam | cut -f 3 | cat /dev/stdin transcripts.list | sort | uniq -c | awk '{print
$2, $1-1}' > female_1_perfectmatch.counts

cat male_1_perfectmatch.sam | cut -f 3 | cat /dev/stdin transcripts.list | sort | uniq -c | awk '{print $2,
$1-1}' > male_1_perfectmatch.counts

module load R

Rscript merge_results.R
module load python

python contigs_list.py

perl -ne 'if(/^>(\S+)/){$c=$i{$1}}$c?print:chomp;$i{$_}=1 if @ARGV' contigs_list.txt
mansoni_200.fasta > mansoni_transcripts_filtered_by_coverage.fasta

rm contigs_list.txt

rm *.counts

module load blat
perl SpliceFinder_2.pl mansoni_transcripts_filtered_by_coverage.fasta

echo "Add SM_W_ to the names of the transcripts"
perl -p -e "s/^>/>SM_W_/g" mansoni_transcripts_filtered_by_coverage.fasta.long >
Final_Mansoni_W_transcripts_renamed.fasta
```

merge_results.R:

```
male_1 <-read.table("male_1_perfectmatch.counts", head=F, sep="")
female_1 <-read.table("female_1_perfectmatch.counts", head=F, sep="")
male_vs_female <- merge(male_1,female_1,by.x="V1", by.y="V1")
write.csv(male_vs_female, file = "transcripts_counts.csv")
```

contigs_list.py:

```
import pandas as pd
import numpy as np
df1 = pd.read_csv("transcripts_counts.csv")
df1=df1.drop(['Unnamed: 0'], axis='columns')
df1 = df1.rename(columns={'V1': 'Contig','V2.x': 'Male_1','V2.y': 'Female_1'})
df1['Ratio']=df1['Male_1']/(df1['Male_1']+df1['Female_1'])
df2=df1[(df1['Ratio']<=0.1)]
df2=df2[(df2['Female_1']>=20)]
```

```
text_file = open("contigs_list.txt", "w")
for item in df2['Contig']:
   text_file.write(('%s' % item +'\n'))
text_file.close()
```

2.8 Final list of W-candidates All transcripts that had less than 20 perfectly matching female reads and a ratio of (male/(male+female)) perfect matches of more than 0.1 were removed. In order to have a more comprehensive set of candidates, the annotated W transcripts (32) which were either recovered partially or not recovered at all were added to our set of candidates.

Adding annotated W transcripts:

```
module load blat
perl -ne 'if(/^>(\S+)/){$c=$i{$1}}$c?print:chomp;$i{$_}=1 if @ARGV' w_genes_list.txt
schistosoma_mansoni.PRJEA36577.WBPS14.CDS_transcripts.fa > annotated_w_genes_CDS.fasta

cat Final_Mansoni_W_transcripts_renamed.fasta annotated_w_genes_CDS.fasta >
Final_combined_mansoni_set.fasta

perl SpliceFinder_2.pl Final_combined_mansoni_set.fasta
```

w_genes_list includes all the isoforms of the annotated W transcripts in the list below that are in the reference CDS:

```
Smp_301330
Smp_318640
Smp_318650
Smp_020920
Smp_318660
Smp_331650
Smp_318670
Smp_318680
Smp_318690
Smp_318710
Smp_323380
Smp_310950
Smp_312650
Smp_320660
Smp_320670
Smp_320680
Smp_320940
Smp_335650
Smp_324690
Smp_324700
Smp_324710
Smp_336560
Smp_325490
Smp_327030
Smp_308070
Smp_344700
Smp_317860
Smp_317870
Smp_336600
Smp_146490
Smp_045040
Smp_303540
```

Supplementary Code 3: *S. japonicum* k-mer based identification and assembly of candidate W-derived transcripts

3.1 k-mer pipeline

```
module load java
module load bbmap

kmer=31

echo "### Step 0: Kmer pipeline starting"
kmercountexact.sh k=$kmer in1=40641_GTCCGC_1_paired.fq.gz
in2=40641_GTCCGC_2_paired.fq.gz out=female_dna_mer_1.fa

kmercountexact.sh k=$kmer in=SRR4267990_trimmed.fq.gz
out=sfemale_rna_schistosomula_mer_1.fa

kmercountexact.sh k=$kmer in=SRR4279491_trimmed.fq.gz
out=sfemale_rna_schistosomula_mer_2.fa

kmercountexact.sh k=$kmer in1=SRR1421523_1_paired.fastq in2=SRR1421523_2_paired.fastq
out=sfemale_rna_mature_adult_mer_1.fa

cat sfemale_rna_schistosomula_mer_1.fa sfemale_rna_schistosomula_mer_2.fa
sfemale_rna_mature_adult_mer_1.fa > sfemale_rna_mer_1.fa

kmercountexact.sh k=$kmer in=female_dna_mer_1.fa,sfemale_rna_mer_1.fa
out=shared_female_mer.fa mincount=2

bbduk.sh k=$kmer in=shared_female_mer.fa out=female_specific_mers.fasta
ref=40640_ACTGAT_1_paired.fq.gz,40640_ACTGAT_2_paired.fq.gz -Xmx350g

bbduk.sh k=$kmer in=female_specific_mers.fasta out=v_female_specific_mers.fasta
ref=SRR4267991_trimmed.fq.gz,SRR4279496_trimmed.fq.gz

bbduk.sh k=$kmer in=v_female_specific_mers.fasta out=v_v_female_specific_mers.fasta
ref=SRR1421524_1_paired.fastq,SRR1421524_2_paired.fastq

bbduk.sh k=$kmer in=SRR4267990_trimmed.fq.gz outm=schistosomula_specific_rna_reads_1.fastq
ref=v_v_female_specific_mers.fasta minkmerfraction=0.4

bbduk.sh k=$kmer in=SRR4279491_trimmed.fq.gz outm=schistosomula_specific_rna_reads_2.fastq
ref=v_v_female_specific_mers.fasta minkmerfraction=0.4

bbduk.sh k=$kmer in1=SRR1421523_1_paired.fastq in2=SRR1421523_2_paired.fastq
outm1=mature_adult_specific_rna_reads_1_1.fastq
outm2=mature_adult_specific_rna_reads_1_2.fastq ref=v_v_female_specific_mers.fasta
minkmerfraction=0.4
```

3.2 Concatenation before assembly

```
cat schistosomula_specific_rna_reads_1.fastq schistosomula_specific_rna_reads_2.fastq >
female_specific_rna_reads.fastq
```

3.3 *SOAPdenovo-trans* config file used for the assembly

```
#maximal read length

max_rd_len=101

[LIB]

#maximal read length in this lib

rd_len_cutof=101

#average insert size

avg_ins=50

#if sequence needs to be reversed

reverse_seq=0

#in which part(s) the reads are used

asm_flags=3

#minimum aligned length to contigs for a reliable read location (at least 32 for short insert size)

map_len=32

#fastq file for read 1

q1=mature_adult_specific_rna_reads_1_1.fastq

q2=mature_adult_specific_rna_reads_1_2.fastq

q=female_specific_rna_reads.fastq
```

3.4 *SOAPdenovo-trans* assembly

```
echo "### Step 1: Assembly of Candidates"
module load SOAPdenovoTrans

srun SOAPdenovo-Trans-31mer all -s soapconfig.txt -K 15 -o TranscriptAssembly -p 16 1>
TranscriptAssembly.stdout 2> TranscriptAssembly.stderr
```

3.5 *faFilter* to remove transcripts shorter than 200bp

```
echo "### Step 2: Removing candidates shorter than 200 bp"
module load fafilter
srun faFilter -minSize=200  TranscriptAssembly.scafSeq japonicum_200.fasta
```

3.6 *Bowtie2* to map the male and female genomic reads to the W candidates and count perfect matches

```
echo "### Step 3: Filtering Stage 1"
module load bowtie2/2.3.4.1

srun bowtie2-build japonicum_200.fasta transcripts_w
```

```
srun bowtie2 -p 8 --no-unal --no-hd --no-sq -x transcripts_w -U SRR5054672_1.fastq -S mixed_1.sam

srun bowtie2 -p 8 --no-unal --no-hd --no-sq -x transcripts_w -U SRR5054673_1.fastq -S mixed_2.sam

srun bowtie2 -p 8 --no-unal --no-hd --no-sq -x transcripts_w -U SRR5054701_1.fastq -S mixed_3.sam

srun bowtie2 -p 8 --no-unal --no-hd --no-sq -x transcripts_w -U SRR5054524_1.fastq -S male_1.sam

srun bowtie2 -p 8 --no-unal --no-hd --no-sq -x transcripts_w -U SRR5054649_1.fastq -S male_2.sam

srun bowtie2 -p 8 --no-unal --no-hd --no-sq -x transcripts_w -U SRR5054671_1.fastq -S male_3.sam

srun bowtie2 -p 8 --no-unal --no-hd --no-sq -x transcripts_w -U SRR5054674_1.fastq -S male_4.sam


awk '($6=="100M")' mixed_1.sam | grep 'NM:i:0' > mixed_1_perfectmatch.sam

awk '($6=="100M")' mixed_2.sam | grep 'NM:i:0' > mixed_2_perfectmatch.sam

awk '($6=="100M")' mixed_3.sam | grep 'NM:i:0' > mixed_3_perfectmatch.sam

awk '($6=="100M")' male_1.sam | grep 'NM:i:0' > male_1_perfectmatch.sam

awk '($6=="100M")' male_2.sam | grep 'NM:i:0' > male_2_perfectmatch.sam

awk '($6=="100M")' male_3.sam | grep 'NM:i:0' > male_3_perfectmatch.sam

awk '($6=="100M")' male_4.sam | grep 'NM:i:0' > male_4_perfectmatch.sam

grep '>' japonicum_200.fasta | perl -pi -e 's/>//gi' | perl -pi -e 's/ .*//gi' > transcripts.list

cat mixed_1_perfectmatch.sam | cut -f 3 | cat /dev/stdin transcripts.list | sort | uniq -c | awk '{print
$2, $1-1}' > mixed_1_perfectmatch.counts

cat mixed_2_perfectmatch.sam | cut -f 3 | cat /dev/stdin transcripts.list | sort | uniq -c | awk '{print
$2, $1-1}' > mixed_2_perfectmatch.counts

cat mixed_3_perfectmatch.sam | cut -f 3 | cat /dev/stdin transcripts.list | sort | uniq -c | awk '{print
$2, $1-1}' > mixed_3_perfectmatch.counts

cat male_1_perfectmatch.sam | cut -f 3 | cat /dev/stdin transcripts.list | sort | uniq -c | awk '{print $2,
$1-1}' > male_1_perfectmatch.counts

cat male_2_perfectmatch.sam | cut -f 3 | cat /dev/stdin transcripts.list | sort | uniq -c | awk '{print $2,
$1-1}' > male_2_perfectmatch.counts

cat male_3_perfectmatch.sam | cut -f 3 | cat /dev/stdin transcripts.list | sort | uniq -c | awk '{print $2,
$1-1}' > male_3_perfectmatch.counts

cat male_4_perfectmatch.sam | cut -f 3 | cat /dev/stdin transcripts.list | sort | uniq -c | awk '{print $2,
$1-1}' > male_4_perfectmatch.counts

module load R

Rscript merge_results.R

module load python

python contigs_list.py
```

```
perl -ne 'if(/^>(\S+)/){$c=$i{$1}}$c?print:chomp;$i{$_}=1 if @ARGV' contigs_list.txt
japonicum_200.fasta > Japonicum_transcripts_filtered_by_coverage.fasta
```

merge_results.R:

```
male_1 <-read.table("male_1_perfectmatch.counts", head=F, sep="")
male_2 <-read.table("male_2_perfectmatch.counts", head=F, sep="")
male_3 <-read.table("male_3_perfectmatch.counts", head=F, sep="")
male_4 <-read.table("male_4_perfectmatch.counts", head=F, sep="")
mixed_1 <-read.table("mixed_1_perfectmatch.counts", head=F, sep="")
mixed_2 <-read.table("mixed_2_perfectmatch.counts", head=F, sep="")
mixed_3 <-read.table("mixed_3_perfectmatch.counts", head=F, sep="")
male_vs_mixed <- merge(male_1,male_2,by.x="V1", by.y="V1")
male_vs_mixed <- merge(male_vs_mixed,male_3,by.x="V1", by.y="V1")
male_vs_mixed <- merge(male_vs_mixed,male_4,by.x="V1", by.y="V1")
male_vs_mixed <- merge(male_vs_mixed,mixed_1,by.x="V1", by.y="V1")
male_vs_mixed <- merge(male_vs_mixed,mixed_2,by.x="V1", by.y="V1")
male_vs_mixed <- merge(male_vs_mixed,mixed_3,by.x="V1", by.y="V1")
write.csv(male_vs_mixed, file = "transcripts_counts.csv")
```

contigs_list.py:

```
import pandas as pd
import numpy as np
df1 = pd.read_csv("transcripts_counts.csv")
df1=df1.drop(['Unnamed: 0'], axis='columns')
df1 = df1.rename(columns={'V1': 'Contig','V2.x': 'Male_1','V2.y': 'Male_2','V2.x.1': 'Male_3','V2.y.1':
'Male_4','V2.x.2': 'Mixed_1','V2.y.2': 'Mixed_2','V2':'Mixed_3'})
df1['Mixed_sum']=df1['Mixed_1']+df1['Mixed_2']+df1['Mixed_3']
df1['Male_sum']=df1['Male_1']+df1['Male_2']+df1['Male_3']+df1['Male_4']
df1['Ratio']=df1['Male_sum']/(df1['Male_sum']+df1['Mixed_sum'])
df2=df1[(df1['Ratio']<=0.1)]
df2=df2[(df2['Mixed_sum']>=15)]
text_file = open("contigs_list.txt", "w")
for item in df2['Contig']:
   text_file.write(('%s' % item +'\n'))
text_file.close()
```

3.7 The long k-mer female assembly used to improve the *S. japonicum* candidates

3.7.1. The config file for the assembly: soapconfig_trans.txt

```
#maximal read length
max_rd_len=150
[LIB]
#maximal read length in this lib
rd_len_cutof=150
#average insert size
avg_ins=0
#if sequence needs to be reversed
reverse_seq=0
#in which part(s) the reads are used
asm_flags=3
```

```
#minimum aligned length to contigs for a reliable read location (at least 32 for short insert size)
map_len=52
#fastq file for read 1
q=SRR4296944.fastq

[LIB]
#maximal read length in this lib
rd_len_cutof=150
#average insert size
avg_ins=0
#if sequence needs to be reversed
reverse_seq=0
#in which part(s) the reads are used
asm_flags=3
#minimum aligned length to contigs for a reliable read location (at least 32 for short insert size)
map_len=52
#fastq file for read 1
q=SRR4296942.fastq

[LIB]
#maximal read length in this lib
rd_len_cutof=150
#average insert size
avg_ins=0
#if sequence needs to be reversed
reverse_seq=0
#in which part(s) the reads are used
asm_flags=3
#minimum aligned length to contigs for a reliable read location (at least 32 for short insert size)
map_len=52
#fastq file for read 1
q=SRR4296940.fastq

[LIB]
#maximal read length in this lib
rd_len_cutof=150
#average insert size
avg_ins=0
#if sequence needs to be reversed
reverse_seq=0
#in which part(s) the reads are used
asm_flags=3
#minimum aligned length to contigs for a reliable read location (at least 32 for short insert size)
map_len=52
#fastq file for read 1
q=SRR4296938.fastq

[LIB]
#maximal read length in this lib
rd_len_cutof=150
#average insert size
avg_ins=0
#if sequence needs to be reversed
reverse_seq=0
#in which part(s) the reads are used
asm_flags=3
#minimum aligned length to contigs for a reliable read location (at least 32 for short insert size)
map_len=52
#fastq file for read 1
q=SRR4296936.fastq
```

```
[LIB]
#maximal read length in this lib
rd_len_cutof=150
#average insert size
avg_ins=0
#if sequence needs to be reversed
reverse_seq=0
#in which part(s) the reads are used
asm_flags=3
#minimum aligned length to contigs for a reliable read location (at least 32 for short insert size)
map_len=52
#fastq file for read 1
q=SRR4296934.fastq

[LIB]
#maximal read length in this lib
rd_len_cutof=150
#average insert size
avg_ins=0
#if sequence needs to be reversed
reverse_seq=0
#in which part(s) the reads are used
asm_flags=3
#minimum aligned length to contigs for a reliable read location (at least 32 for short insert size)
map_len=52
#fastq file for read 1
q=SRR4296931.fastq

[LIB]
#maximal read length in this lib
rd_len_cutof=150
#average insert size
avg_ins=0
#if sequence needs to be reversed
reverse_seq=0
#in which part(s) the reads are used
asm_flags=3
#minimum aligned length to contigs for a reliable read location (at least 32 for short insert size)
map_len=52
#fastq file for read 1
q=SRR4292306.fastq

[LIB]
#maximal read length in this lib
rd_len_cutof=150
#average insert size
avg_ins=0
#if sequence needs to be reversed
reverse_seq=0
#in which part(s) the reads are used
asm_flags=3
#minimum aligned length to contigs for a reliable read location (at least 32 for short insert size)
map_len=52
#fastq file for read 1
q=SRR4292301.fastq

[LIB]
#maximal read length in this lib
rd_len_cutof=150
```

```
#average insert size
avg_ins=0
#if sequence needs to be reversed
reverse_seq=0
#in which part(s) the reads are used
asm_flags=3
#minimum aligned length to contigs for a reliable read location (at least 32 for short insert size)
map_len=52
#fastq file for read 1
q=SRR4292299.fastq

[LIB]
#maximal read length in this lib
rd_len_cutof=150
#average insert size
avg_ins=0
#if sequence needs to be reversed
reverse_seq=0
#in which part(s) the reads are used
asm_flags=3
#minimum aligned length to contigs for a reliable read location (at least 32 for short insert size)
map_len=52
#fastq file for read 1
q=SRR4292292.fastq

[LIB]
#maximal read length in this lib
rd_len_cutof=150
#average insert size
avg_ins=0
#if sequence needs to be reversed
reverse_seq=0
#in which part(s) the reads are used
asm_flags=3
#minimum aligned length to contigs for a reliable read location (at least 32 for short insert size)
map_len=52
#fastq file for read 1
q=SRR4292206.fastq
```

3.7.2 *De novo* 65-mer female assembly with *SOAPdenovoTrans*

```
module load SOAPdenovoTrans

SOAPdenovo-Trans-127mer all -s soapconfig_trans.txt -K 65 -o TranscriptAssemblyK65 -p 16 1>
TranscriptAssemblyK65.stdout 2> TranscriptAssemblyK65.stderr
```

3.7.3 Improvement of the assembly of the W-Candidates using the *de novo* 65-mer female assembly

```
echo "### Step 4: Reassembly of Candidates"
module load blat

module load  cap3/02-10-15

blat -minScore=50 TranscriptAssemblyK65.scafSeq
Japonicum_transcripts_filtered_by_coverage.fasta /dev/stdout | awk '(($2/($1+
$2+0.00000001))<0.01)' | cut -f 14 | sort | uniq | ~/seqtk/seqtk subseq
```

```
TranscriptAssemblyK65.scafSeq /dev/stdin > K65_maptoyourcandidate.fa

cap3 K65_maptoyourcandidate.fa -h 80 -o 40

#merge cap3 output with the filtered transcripts
cat Japonicum_transcripts_filtered_by_coverage.fasta K65_maptoyourcandidate.fa.cap.contigs >
K65_plus_transcripts.fasta

#remove duplicates

perl SpliceFinder_2.pl K65_plus_transcripts.fasta

#Add SJ_W_ to the names of the transcripts
perl -p -e "s/^>/>SJ_W_/g" K65_plus_transcripts.fasta.long >
Final_Japonicum_W_transcripts_renamed.fasta
```

3.7.4 Using coverage analysis to filter the improved set of candidates

```
echo "### Step 5: Filtering Stage 2"
srun bowtie2-build Final_Japonicum_W_transcripts_renamed.fasta transcripts_w

srun bowtie2 -p 8 --no-unal --no-hd --no-sq -x transcripts_w -U SRR5054672_1.fastq -S mixed_1.sam

srun bowtie2 -p 8 --no-unal --no-hd --no-sq -x transcripts_w -U SRR5054673_1.fastq -S mixed_2.sam

srun bowtie2 -p 8 --no-unal --no-hd --no-sq -x transcripts_w -U SRR5054701_1.fastq -S mixed_3.sam

srun bowtie2 -p 8 --no-unal --no-hd --no-sq -x transcripts_w -U SRR5054524_1.fastq -S male_1.sam

srun bowtie2 -p 8 --no-unal --no-hd --no-sq -x transcripts_w -U SRR5054649_1.fastq -S male_2.sam

srun bowtie2 -p 8 --no-unal --no-hd --no-sq -x transcripts_w -U SRR5054671_1.fastq -S male_3.sam

srun bowtie2 -p 8 --no-unal --no-hd --no-sq -x transcripts_w -U SRR5054674_1.fastq -S male_4.sam

awk '($6=="100M")' mixed_1.sam | grep 'NM:i:0' > mixed_1_perfectmatch.sam

awk '($6=="100M")' mixed_2.sam | grep 'NM:i:0' > mixed_2_perfectmatch.sam

awk '($6=="100M")' mixed_3.sam | grep 'NM:i:0' > mixed_3_perfectmatch.sam

awk '($6=="100M")' male_1.sam | grep 'NM:i:0' > male_1_perfectmatch.sam

awk '($6=="100M")' male_2.sam | grep 'NM:i:0' > male_2_perfectmatch.sam

awk '($6=="100M")' male_3.sam | grep 'NM:i:0' > male_3_perfectmatch.sam

awk '($6=="100M")' male_4.sam | grep 'NM:i:0' > male_4_perfectmatch.sam

grep '>' Final_Japonicum_W_transcripts_renamed.fasta | perl -pi -e 's/>//gi' | perl -pi -e 's/ .*//gi' >
transcripts.list

cat mixed_1_perfectmatch.sam | cut -f 3 | cat /dev/stdin transcripts.list | sort | uniq -c | awk '{print
$2, $1-1}' > mixed_1_perfectmatch.counts

cat mixed_2_perfectmatch.sam | cut -f 3 | cat /dev/stdin transcripts.list | sort | uniq -c | awk '{print
```

```
$2, $1-1}' > mixed_2_perfectmatch.counts

cat mixed_3_perfectmatch.sam | cut -f 3 | cat /dev/stdin transcripts.list | sort | uniq -c | awk '{print
$2, $1-1}' > mixed_3_perfectmatch.counts

cat male_1_perfectmatch.sam | cut -f 3 | cat /dev/stdin transcripts.list | sort | uniq -c | awk '{print $2,
$1-1}' > male_1_perfectmatch.counts

cat male_2_perfectmatch.sam | cut -f 3 | cat /dev/stdin transcripts.list | sort | uniq -c | awk '{print $2,
$1-1}' > male_2_perfectmatch.counts

cat male_3_perfectmatch.sam | cut -f 3 | cat /dev/stdin transcripts.list | sort | uniq -c | awk '{print $2,
$1-1}' > male_3_perfectmatch.counts

cat male_4_perfectmatch.sam | cut -f 3 | cat /dev/stdin transcripts.list | sort | uniq -c | awk '{print $2,
$1-1}' > male_4_perfectmatch.counts

Rscript merge_results.R

python contigs_list.py

perl -ne 'if(/^>(\S+)/){$c=$i{$1}}$c?print:chomp;$i{$_}=1 if @ARGV' contigs_list.txt
Final_Japonicum_W_transcripts_renamed.fasta > final_w_japonicum.fasta
```

3.7.5 Generate a male transcriptome assembly and recover the "best hits" from it to avoid hybrid assemblies of ZW

```
#Trimming the reads:
module load java
srun java -jar ~/Trimmomatic-0.36/trimmomatic-0.36.jar PE -phred33 SRR8175618_1.fastq
SRR8175618_2.fastq  SRR8175618_1_paired.fastq SRR8175618_1_unpaired.fastq
SRR8175618_2_paired.fastq SRR8175618_2_unpaired.fastq
ILLUMINACLIP:~/Trimmomatic-0.36/adapters/TruSeq3-PE.fa:2:30:10 LEADING:3 TRAILING:3
SLIDINGWINDOW:4:15 MINLEN:140

#Assembly using Trinity:

module load java
module load samtools/1.10
module load jellyfish/2.3.0
module load bowtie2/2.3.4.1
module load python/3.8.5
module load salmon/0.13.1


~/trinityrnaseq-v2.11.0/Trinity --seqType fa --left SRR8175618_1_paired_renamed.fasta --right
SRR8175618_2_paired_renamed.fasta --CPU XX --max_memory XXG

#Cap3

module load  cap3/02-10-15

cap3 Trinity.fasta -h 80 -o 40

cat Trinity.fasta.cap.contigs Trinity.fasta.cap.singlets > Trinity_cap3.fasta
```

3.7.6 Identify the Z-homologs of our candidates

Extract the "best hits" from *Blat* alignment and using them as our putative Z:

```
echo "### Step 7: Finding the Z candidates"

module load blat
blat -minScore=50 Trinity_cap3.fasta final_w_japonicum.fasta W_vs_trinity.blat -t=dnax -q=dnax

sort -k 10 W_vs_trinity.blat > W_vs_trinity.blat.sorted

perl 2-besthitblat.pl W_vs_trinity.blat.sorted

cat W_vs_trinity.blat.sorted.besthit | awk '($1>100)' > filtered_z.txt

cat filtered_z.txt | cut -f 14 > names_z_candidates.txt

perl -ne 'if(/^>(\S+)/){$c=$i{$1}}$c?print:chomp;$i{$_}=1 if @ARGV' names_z_candidates.txt
Trinity_cap3.fasta > Final_Set_Z_Candidates_japonicum.fasta
```

Supplementary Code 4: Strata Identification

4.1 *De novo* determination of Z-specific regions in *S. mansoni*

1.bowtie2_mansoni.sh

Male (ERR562989) and female (ERR562990) DNA reads were aligned to the latest version of the *S. mansoni* genome (V7, release WBPS14, obtained from the WormBase Parasite database on the 30th of October 2020)

```
#! /bin/bash

# OPTIONS

#SBATCH --job-name="bowtie2"
#SBATCH --output=bowtie2_mansoni.out
#SBATCH --cpus-per-task=3
#SBATCH --mem=25G
#SBATCH --time=48:00:00

# MORE INFOS

#-# last edit: 30-10-2020
#-# usage: qsub 1.bowtie2_mansoni.sh
#-# prog required: bowtie (v2.3.4.1)

### START

inf="~/path-to-the-reads"
genome="~/path-to-the-genome/genome.fa"
ouf="~/path-to-output"

mkdir $ouf
```

```
module load bowtie2/2.3.4.1

## building index

bowtie2-build -f $genome mansoni_index
mv mansoni_index* $ouf

## aligning

bowtie2 -x $ouf/mansoni_index -1 $inf/ERR562989_forward_paired.fq.gz -2
$inf/ERR562989_reverse_paired.fq.gz -p 3 -S $ouf/mansoni_males.sam

bowtie2 -x $ouf/mansoni_index -1 $inf/ERR562990_forward_paired.fq.gz -2
$inf/ERR562990_reverse_paired.fq.gz -p 3 -S $ouf/mansoni_females.sam

### END
```

2.soapcov_mansoni.sh

Bowtie2 outputs were filtered, and uniquely mapped reads were used as input for *soapcoverage* (analysis of genomic coverage)

```
#! /bin/bash

# OPTIONS

#SBATCH --job-name="soapcov"
#SBATCH --output=soapcov.out
#SBATCH --mem=50G
#SBATCH --time=24:00:00

# MORE INFOS

#-# last edit: 13-11-2020
#-# usage: qsub 2.soapcov_mansoni.sh
#-# prog required: soap coverage

### START

#load module

module load soap/coverage

#define variable
inf="~/path-to-bowtie2-output"
genome="~/path-to-the-genome/genome.fa"
ouf="~/path-to-output/" # do not forget the final “/”
win=10000

mkdir $ouf

#Select only uniquely mapped reads

cd $inf

for i in `ls | grep .sam`
do
grep -vw 'XS:i' ${i} > ${i}_unique.sam
```

```
done

#Obtain average genomic coverage for each scaffold

cd $inf

for l in `ls | grep _unique.sam`
do
soap.coverage -sam -cvg -i ${l} -onlyuniq -refsingle $genome -o $ouf/${l}.soapcov -window $ouf/$
{l}.win10.soapcov 10000
done

### END
```

3.Zregions_Smansoni.R

Male and female genomic coverages were compared in order to define F:M threshold values (Pipeline illustrated in Sup. Figure 13)

```
### PART I ### DEFINING F:M COVERAGE LIMITS OF Z-SPECIFIC REGIONS IN S. MANSONI

### STEP1: Loci assignment based on 2.5% coverage of autosomes

## Set-up parameters
output="~/path-to-output" ## Here indicate your outputfile
spe="Smans" ##Here indicate the abbreviation of the studied species

## Read data
x=read.table("~/path-to-input/InputFile1_Smansoni_SoapCov_By10kb_ForR_NewG.csv",h=T) ##Here
indicate your input table (5 columns modified SoapCoverage output)
head(x) #5columns: $Scaffold_ID $Window_start $Window_stop $ERR562990_Depth (i.e. female
coverage) $ERR562989_Depth (i.e. male coverage)
tail(x) #5columns
nrow(x) #40958rows==number of analysed windows out of SoapCoverage analysis

## Calculate Log2(F:Mcov)
x$log2_FMcoverage <- log2(x$ERR562990_Depth/x$ERR562989_Depth)
head(x) #6columns: 6th column=="log2_FMcoverage"

## Histogram - Overall genomic coverage distribution
hist(x$log2_FMcoverage, breaks=400, xlim=c(-2,2), ylim=c(0,6000), col="antiquewhite3",
main="F:M coverage distribution - S. mansoni", xlab="log2 (F:M) ratio of coverage")

## Subset depending on the location
x_Autosomes  <- subset(x, x$Scaffold_ID=='SM_V7_1' | x$Scaffold_ID=='SM_V7_2' |
x$Scaffold_ID=='SM_V7_3' | x$Scaffold_ID=='SM_V7_4' |x$Scaffold_ID=='SM_V7_5' |
x$Scaffold_ID=='SM_V7_6' |x$Scaffold_ID=='SM_V7_7')
head(x_Autosomes) #6columns
tail(x_Autosomes) #6columns
nrow(x_Autosomes) #30429rows
x_ZW  <- subset(x, x$Scaffold_ID=='SM_V7_ZW') ## Just to insure that the sum of nrow are correct
nrow(x_ZW) #8839
x_UP <- subset(x, x$Scaffold_ID!='SM_V7_1' & x$Scaffold_ID!='SM_V7_2' & x$Scaffold_ID!
='SM_V7_3' & x$Scaffold_ID!='SM_V7_4' & x$Scaffold_ID!='SM_V7_5' & x$Scaffold_ID!='SM_V7_6' &
x$Scaffold_ID!='SM_V7_7' & x$Scaffold_ID!='SM_V7_8')
nrow(x_UP) #10529

## Determining minCov_Aut & maxCov_Aut
maxCov_Aut <- quantile(x_Autosomes$log2_FMcoverage,0.975,na.rm=T)
```

```
maxCov_Aut
maxCov_Aut_Graph <- round(maxCov_Aut,digits = 4)
minCov_Aut  <- quantile(x_Autosomes$log2_FMcoverage,0.025,na.rm=T) #### Further used limit
minCov_Aut
minCov_Aut_Graph <- round(minCov_Aut,digits = 4)

## Histogram - Autosomes
hist(x_Autosomes$log2_FMcoverage, breaks=600, xlim=c(-1,1), ylim=c(0,2500),
col="gray37",border="gray50", main="F:M coverage distribution - Autosomes", xlab="log2 (F:M)
ratio of coverage")
abline(v=maxCov_Aut,col="gray37",lwd=2,lty=2)
abline(v=minCov_Aut,col="sienna2",lwd=2,lty=2)
text((minCov_Aut_Graph-0.05),1000,paste("2.5%min_Aut: ",
minCov_Aut_Graph),srt=90,col="sienna2")
text((maxCov_Aut_Graph-0.05),1000,paste("2.5%max_Aut: ",
maxCov_Aut_Graph),srt=90,col="gray37")

## Visualizing first step filter

# Subset Chr_ZW
x_ZW  <- subset(x, x$Scaffold_ID=='SM_V7_ZW')

# Define color
colFactor <- x_ZW$log2_FMcoverage
colF <- rep("antiquewhite2",length(colFactor)) ; colF[which(colFactor>(minCov_Aut))] <- "gray37" ;
colF[which(colFactor<(minCov_Aut))] <- "sienna2"
colF

# Plot coverage ratio F:M
plot(as.numeric(paste(x_ZW$Window_start)),
log2(as.numeric(paste(x_ZW$ERR562990_Depth))/as.numeric(paste(x_ZW$ERR562989_Depth))),
col=colF, ylim=c(-2,2), pch=16, xlab='Position on the Z-chromosome', ylab='log2 (F:M) ratio of
coverage', main="F:M coverage along S. mansoni Z-chromosome")
legend(x="bottomright",paste(c("2.5% min_Aut","PAR","Non-
PAR")),pch=c(NA,16,16),lty=c(2,NA,NA),lwd=c(2,NA,NA),col=c("sienna2","gray37","sienna2"),bty="
n")
abline(h=minCov_Aut, col="sienna2", lty=2, lw=2)

## Generating first step table

# Define flags
Z <- which((x_ZW$log2_FMcoverage) < minCov_Aut)
PAR <- which((x_ZW$log2_FMcoverage) > minCov_Aut)
flag <- rep("Ambiguous",nrow(x_ZW)) ; flag[Z] <- "Z" ; flag[PAR] <- "PAR"
x_ZW <- cbind(x_ZW,flag)
head(x_ZW)
nrow(x_ZW) #8839
nrow(subset(x_ZW,x_ZW$flag=="PAR")) #5431
nrow(subset(x_ZW,x_ZW$flag=="Z")) #3337
nrow(subset(x_ZW,x_ZW$flag=="Ambiguous")) #71

#Write filtered final table with flags
coln=NULL ; for(j in 1:length(colnames(x_ZW))){coln <- c(coln,paste(colnames(x_ZW)
[j],spe,sep="_"))}
write.table(x_ZW,paste(output,"InputFile2_Zonly_10kb",".txt",sep=""),col.names=coln,row.names=F
)


### STEP2: Loci assignment based on 1% coverage of Z-candidates
```

```
## Read data
x_Z=read.table(paste(output,"InputFile2_Zonly_10kb",".txt",sep=""),h=T,fill=TRUE)
head(x_Z) #7columns: 7th column=="flag_Smans"
x_Z  <- subset(x_Z, x_Z$flag_Smans=="Z")
head(x_Z) #7columns: 7th column=="flag_Smans" should be only Z
tail(x_Z) #7columns
nrow(x_Z) #3337

## Determining minCov_Aut & maxCov_Aut
maxCov_Z <- quantile(x_Z$log2_FMcoverage_Smans,0.99,na.rm=T)
maxCov_Z #-0.3541866
maxCov_Z_Graph <- round(maxCov_Z,digits = 4)
minCov_Z  <- quantile(x_Z$log2_FMcoverage_Smans,0.01,na.rm=T) #### What's matter
minCov_Z #-1.101588
minCov_Z_Graph <- round(maxCov_Z,digits = 4)

## Histogram - Z chromosome
hist(x_Z$log2_FMcoverage_Smans, breaks=100, xlim=c(-2,0), ylim=c(0,200), col="sienna2",
border="sienna1", main="F:M coverage distribution - Non-autosomes", xlab="log2 (F:M) ratio of
coverage")
abline(v=maxCov_Z,col="gray37",lwd=2,lty=2)
abline(v=minCov_Z,col="sienna2",lwd=2,lty=2)
text(-1.15,100,paste("1%min_Z: ", minCov_Z_Graph),srt=90,col="sienna2")
text(-0.43,100,paste("1%max_Z: ", maxCov_Z_Graph),srt=90,col="gray37")

## Visualizing second step filter
x=read.table("~/path-to-input/InputFile1_Smansoni_SoapCov_By10kb_ForR_NewG.csv",h=T) ##Here
indicate your input table (5 columns modified SoapCoverage output)
x$log2_FMcoverage <- log2(x$ERR562990_Depth/x$ERR562989_Depth)
x_ZW  <- subset(x, x$Scaffold_ID=='SM_V7_ZW')

# Plot coverage ratio F:M with ambiguous regions based on coverage only
colFactor <- x_ZW$log2_FMcoverage
colF <- rep("antiquewhite2",length(colFactor)) ; colF[which(colFactor>(minCov_Aut))] <- "gray37" ;
colF[which(colFactor<(maxCov_Z))] <- "sienna2"
plot(as.numeric(paste(x_ZW$Window_start)),
log2(as.numeric(paste(x_ZW$ERR562990_Depth))/as.numeric(paste(x_ZW$ERR562989_Depth))),
col=colF,pch=16, ylim=c(-2,2), xlab='Position on the Z-chromosome', ylab='log2 (F:M) ratio of
coverage', main="F:M genomic coverage distribution along S. mansoni Z-chromosome")
abline(h=minCov_Aut, col="sienna2", lty=2, lwd=2)
abline(h=maxCov_Z, col="gray37", lty=2, lwd=2)
legend(x="bottomright",paste(c("2.5% min_Aut","1% max_Z","Non-
Zlinked","Zlinked","Ambiguous")),pch=c(NA,NA,16,16,16),lty=c(2,2,NA,NA,NA),lwd=c(2,2,NA,NA,NA)
,col=c("sienna2","gray37","gray37","sienna2","antiquewhite2"),bty="n")
```

4.GeneSelector.pl

We applied the above determined threshold to the entire ZW linkage group and the final classification of Z-specific and pseudoautosomal regions is reported in Sup. Dataset 5 (sheet #1). In this table, we corrected the Z-specific content by systematically excluding windows for which the coverage value is not consistent with the adjacent windows : we added them to the category named « ambiguous ». Finally, annotated CDS were individually assigned to the Z or the PAR region, based on their coordinates. When a gene overlapped with an ambiguous window, it was tagged « ambiguous » as well thanks to the 4.GeneSelector.pl script below.

```
#!/usr/bin/perl

print "input are: a list of coordinates to exclude ($start $space $end), and a CDS file ($genename
$chrom $start $end)";
print "usage: perl 4.GeneSelector.pl InputFile3_windows-to-exclude.csv InputFile4_mansoni-gene-
coordinates.cnv\n";

my $input = $ARGV[0];
my $ensembl = $ARGV[1];
open (INPUT, "$input");
open (GENES, "$ensembl");
open (RESULTS, ">>$ensembl.Windows.txt");

while ($line = <INPUT>) {
	push (@windows, $line);
	}

while ($gline = <GENES>)
	 {
	chomp $gline;
	chomp $gline;
	$gline =~ s/\n//g;
	($gene, $chrom, $start, $end)=split(/\t/, $gline);
	if ($gene ne "Gene_ID")
		{
		foreach $window (@windows)
			{
			($winchrom, $winstart, $winend)=split(/\t/, $window);
			if ($chrom eq $winchrom)
				{
				if (($start>$winstart && $start<$winend) || ($end>$winstart &&
$end<$winend) || ($start<$winstart && $end>$winend))
					{
					print RESULTS "$gline\n";
					}
				else
					{

					}
				}
			}
		}
	else {}
	}
```

4.2 Inference of the location of *S. japonicum* scaffolds along the *S. mansoni* Z-chromosome

5.blat.sh

We mapped *S. mansoni* CDS to *S. japonicum* scaffolds (obtained from the WormBase Parasite database on the 30th of October 2020), using *blat* with a translated query and database.

```
#! /bin/bash
#SBATCH --job-name="blat"
#SBATCH --mem=50G
#SBATCH --time=48:00:00
```

```
#SBATCH --output=1.blat.out

#-# last edit: 30-10-2020
#-# usage: qsub 5.blat.sh
#-# prog required: BLAT_v36x1
#-# description: Align with BLAT

## define variables:
CDS="~/path-to-mansoni-CDS/schistosoma_mansoni.PRJEA36577.WBPS14.CDS_transcripts.fa"
GENOME="~/path-to-japonicum-genome/schistosoma_japonicum.PRJEA34885.WBPS14.genomic.fa"

### start

module load blat/20170224

blat -q=dnax -t=dnax -minScore=50 ${GENOME} ${CDS} GENOMEjap_CDSmans.blat

sort –k 10 GENOMEjap_CDSmans.blat > GENOMEjap_CDSmans.blat.sorted

### end
```

6.besthitblat.pl

The BLAT alignment was then filtered to keep only the mapping hit with the highest score for each *S. mansoni* CDS.

```
#!/usr/bin/perl
print "make sure your input file is sorted by scaffoldname! sort -k 10";
my $input = $ARGV[0];

open (INPUT, "$input");
open (RESULTS, ">>$input.besthit");

print RESULTS "1match\tmismatch\trep\tNs\tQgapcount\tQgapbases\tTgapcount\tTgapbases\tstrand\
tQname\tQsize\tQstart\tQend\tTname\tTsize\tTstart\tTend\tblockcount\tblockSizes\tqStarts\ttStarts\
n";

$name0="";
$score0=0;
$line0="";

while ($line = <INPUT>) {
($match, $mismatch, $rep, $Ns, $Qgapcount, $Qgapbases, $Tgapcount, $Tgapbases, $strand,
$Qname, $Qsize, $Qstart, $Qend, $Tname, $Tsize, $Tstart, $Tend, $blockcount, $blockSizes,
$qStarts, $tStarts)=split(/\t/, $line);

        if ($Qname eq $name0)
                {
                if ($match > $score0)
                        {
                        $name0=$Qname;
                        $score0=$match;
                        $line0=$line;
                        }
                else
                        {
                        }

                }
```

```
        else
                {
                print RESULTS $line0;
                $name0=$Qname;
            $score0=$match;
            $line0=$line;

                }



        }

print RESULTS $line0;

system "perl -pi -e 's/^[^0-9].*//gi' $input.besthit";
system "perl -pi -e 's/1match/match/gi' $input.besthit";
system "perl -pi -e 's/^\n//gi' $input.besthit";
```

7.blatreverse.pl

When several *S. mansoni* genes overlapped on the *S. japonicum* genome by more than 20 bp, we kept only the highest mapping score.

```
#!/usr/bin/perl

#this program is meant to remove overlapping contigs from blat results
#by reciprocal best blatx hit
#when contigs overlap on target, the one with the highest score is kept (unless the overlap <20bps)

#the input file should be a best hit blat file:
#match       mismatch       rep     Ns      Qgapcount       Qgapbases       Tgapcount
        Tgapbases       strand  Qname   Qsize   Qstart  Qend    Tname   Tsize   Tstart  Tend
        blockcount      blockSizes      qStarts tStarts
#and sorted by Tname!!! sort -k 14!!!

#usage: perl 7.blatreverse.pl besthit-output.sorted

print "make sure your input file is sorted by Tname -k 14!";
my $input = $ARGV[0];

open (INPUT, "$input");
open (RESULTS, ">>$input.nonredundant");

$name0="lalala";
system "echo 'lalala' >> lalala.temp";

while ($line = <INPUT>)
        {
        ($blatscore, $mismatch, $rep, $Ns, $Qgapcount, $Qgapbases, $Tgapcount, $Tgapbases,
$strand, $Qname, $Qsize, $Qstart, $Qend, $Tname, $Tsize, $Tstart, $Tend, $blockcount,
$blockSizes, $qStarts, tStarts)=split(/\t/, $line);

        if ($Tname eq $name0)
                {
                open (TEMP, ">>$Tname.temp");
                print TEMP $line;
                close(TEMP);
                }
        else
```

```
		{

		#first let's deal with gene name0

		#let's find non-overlapping contigs for genes name0
		$sortname="\Q$name0\E";
		system "sort -nr -k 1 $sortname.temp >> $sortname.temp.sorted";
		open(TEMPREAD, "$name0.temp.sorted") or die "could not open tempread";
		print "just opened $name0.temp.sorted\n";
		@start="";
		@end="";
		@counts="";
		$counter=0;


		while ($tempread = <TEMPREAD>)
			{
			print "counter $counter\n";
			$verifier=0;
			($blatscoret, $mismatcht, $rept, $Nst, $Qgapcountt, $Qgapbasest,
$Tgapcountt, $Tgapbasest, $strandt, $Qnamet, $Qsizet, $Qstartt, $Qendt, $Tnamet, $Tsizet,
$Tstartt, $Tendt, $blockcountt, $blockSizest, $qStartst, $tStartst)=split(/\t/, $tempread);

			if ($counter==0)
				{
				print RESULTS $tempread;
				push (@start, $Tstartt);
				push (@end, $Tendt);
				$counter=$counter+1;
				push(@counts, $counter);
				}
			elsif ($counter>0)
				{
				print "starts: @start\n";
				print "counts: @counts\n";

				#let's test if the contig overlaps contigs with higher scores
				foreach $count(@counts)

					{
					$Ne_start="";
					$Ne_end="";

					#allow for 20bp overlap
					#But first make sure there is something in $count, it seems
to run the cycle on empty count first
					if ($count ne "")
						 {
						$Ne_start=$start[$count]+20;
						$Ne_end=$end[$count]-20;
						}
					else
						{
						$verifier=0;

						}


					print "count $count start $Tstartt end $Tendt teststart
```

```
$Ne_start testend $Ne_end verifier $verifier\n";
                                        if ($Tstartt >= $Ne_start && $Tstartt <= $Ne_end)
                                                {
                                                $verifier=$verifier+1;
                                                }
                                        elsif ($Tendt >= $Ne_start && $Tendt <= $Ne_end)
                                                {
                                                $verifier=$verifier+1;
                                                }
                                        elsif ($Tstartt <= $Ne_start && $Tendt >= $Ne_end)
                                                {
                                                $verifier=$verifier+1;
                                                }
                                        else
                                                {
                                                $verifier=$verifier+0;
                                                }
                                        }


                                if ($verifier==0)
                                        {
                                        print RESULTS $tempread;
                                        push (@start, $Tstartt);
                                        push (@end, $Tendt);

                                        $counter=$counter+1;
                                        push(@counts, $counter);
                                        }
                                else
                                        {
                                        #push (@start, $Tstartt);
                                        #push (@end, $Tendt);
                                        #$counter=$counter+1;
                                        #push(@counts, $counter);
                                        }
                                }




                        }




                close(TEMPREAD);
                system "rm \Q$name0\E.temp";
                system "rm \Q$name0\E.temp.sorted";

                $name0=$Tname;

                #finally let's start filling the new file
                open (TEMP, ">>$Tname.temp");
                print TEMP $line;
                close(TEMP);
                }

        }
```

Supplementary Code 5: A description of the process we followed to perform the F_{ST} analysis, from identifying the sex of the Miracidia samples to calculating F_{ST}

5.1 Read Counts for the W candidates

We added three autosomal sequences Sjp_0046990, Sjp_0099780, Sjp_0101650 (chosen randomly) to the W candidates in *S. japonicum*:

```
module load bowtie2/2.3.4.1
module load soap/coverage

srun bowtie2-build Final_Set_W_Candidates_japonicum_plus_autosomes.fasta japonicum_W
srun bowtie2 -p 20 --no-unal --no-hd --no-sq -x japonicum_W -U SRR12363890_1.fastq -S s_1.sam
srun bowtie2 -p 20 --no-unal --no-hd --no-sq -x japonicum_W -U SRR12363891_1.fastq -S s_2.sam
srun bowtie2 -p 20 --no-unal --no-hd --no-sq -x japonicum_W -U SRR12363892_1.fastq -S s_3.sam
srun bowtie2 -p 20 --no-unal --no-hd --no-sq -x japonicum_W -U SRR12363893_1.fastq -S s_4.sam
srun bowtie2 -p 20 --no-unal --no-hd --no-sq -x japonicum_W -U SRR12363894_1.fastq -S s_5.sam
srun bowtie2 -p 20 --no-unal --no-hd --no-sq -x japonicum_W -U SRR12363895_1.fastq -S s_6.sam
srun bowtie2 -p 20 --no-unal --no-hd --no-sq -x japonicum_W -U SRR12363896_1.fastq -S s_7.sam
srun bowtie2 -p 20 --no-unal --no-hd --no-sq -x japonicum_W -U SRR12363897_1.fastq -S s_8.sam
srun bowtie2 -p 20 --no-unal --no-hd --no-sq -x japonicum_W -U SRR12363898_1.fastq -S s_9.sam
srun bowtie2 -p 20 --no-unal --no-hd --no-sq -x japonicum_W -U SRR12363899_1.fastq -S s_10.sam
srun bowtie2 -p 20 --no-unal --no-hd --no-sq -x japonicum_W -U SRR12363900_1.fastq -S s_11.sam
srun bowtie2 -p 20 --no-unal --no-hd --no-sq -x japonicum_W -U SRR12363901_1.fastq -S s_12.sam
srun bowtie2 -p 20 --no-unal --no-hd --no-sq -x japonicum_W -U SRR12363902_1.fastq -S s_13.sam
srun bowtie2 -p 20 --no-unal --no-hd --no-sq -x japonicum_W -U SRR12363903_1.fastq -S s_14.sam
srun bowtie2 -p 20 --no-unal --no-hd --no-sq -x japonicum_W -U SRR12363904_1.fastq -S s_15.sam
srun bowtie2 -p 20 --no-unal --no-hd --no-sq -x japonicum_W -U SRR12363905_1.fastq -S s_16.sam
srun bowtie2 -p 20 --no-unal --no-hd --no-sq -x japonicum_W -U SRR12363906_1.fastq -S s_17.sam
srun bowtie2 -p 20 --no-unal --no-hd --no-sq -x japonicum_W -U SRR12363907_1.fastq -S s_18.sam
srun bowtie2 -p 20 --no-unal --no-hd --no-sq -x japonicum_W -U SRR12363908_1.fastq -S s_19.sam
srun bowtie2 -p 20 --no-unal --no-hd --no-sq -x japonicum_W -U SRR12363909_1.fastq -S s_20.sam
srun bowtie2 -p 20 --no-unal --no-hd --no-sq -x japonicum_W -U SRR12363910_1.fastq -S s_21.sam
```

```
srun bowtie2 -p 20 --no-unal --no-hd --no-sq -x japonicum_W -U SRR12363911_1.fastq -S s_22.sam


for ((i=1; i<=22;i++))
do
awk '($6=="151M")' s_"$i".sam | grep 'NM:i:0' > s_"$i"_perfectmatch.sam

done

grep '>' Final_Set_W_Candidates_japonicum_plus_autosomes.fasta | perl -pi -e 's/>//gi' | perl -pi -e 's/
.*//gi' > japonicum_W.list

for ((i=1;i<=22;i++))
do

cat s_"$i"_perfectmatch.sam | cut -f 3 | cat /dev/stdin japonicum_W.list | sort | uniq -c | awk '{print
$2, $1-1}' > s_"$i"_perfectmatch.counts

cat s_"$i".sam | cut -f 3 | cat /dev/stdin japonicum_W.list | sort | uniq -c | awk '{print $2, $1-1}' >
s_"$i".counts

done
```

5.2 Genomic Coverage

```
module load java

module load bowtie2/2.3.4.1
module load soap/coverage


srun bowtie2-build schistosoma_japonicum.PRJEA34885.WBPS9.genomic.fa japonicum_genome

bowtie2 -x japonicum_genome -1 SRR12363890_1.fastq -2 SRR12363890_2.fastq --end-to-end --
sensitive -p 8 -S 1.sam

bowtie2 -x japonicum_genome -1 SRR12363891_1.fastq -2 SRR12363891_2.fastq --end-to-end --
sensitive -p 8 -S 2.sam

bowtie2 -x japonicum_genome -1 SRR12363892_1.fastq -2 SRR12363892_2.fastq --end-to-end --
sensitive -p 8 -S 3.sam

bowtie2 -x japonicum_genome -1 SRR12363893_1.fastq -2 SRR12363893_2.fastq --end-to-end --
sensitive -p 8 -S 4.sam

bowtie2 -x japonicum_genome -1 SRR12363894_1.fastq -2 SRR12363894_2.fastq --end-to-end --
sensitive -p 8 -S 5.sam

bowtie2 -x japonicum_genome -1 SRR12363895_1.fastq -2 SRR12363895_2.fastq --end-to-end --
sensitive -p 8 -S 6.sam

bowtie2 -x japonicum_genome -1 SRR12363896_1.fastq -2 SRR12363896_2.fastq --end-to-end --
sensitive -p 8 -S 7.sam

bowtie2 -x japonicum_genome -1 SRR12363897_1.fastq -2 SRR12363897_2.fastq --end-to-end --
sensitive -p 8 -S 8.sam
```

```
bowtie2 -x japonicum_genome -1 SRR12363898_1.fastq -2 SRR12363898_2.fastq --end-to-end --
sensitive -p 30 -S 9.sam

bowtie2 -x japonicum_genome -1 SRR12363899_1.fastq -2 SRR12363899_2.fastq --end-to-end --
sensitive -p 30 -S 10.sam

bowtie2 -x japonicum_genome -1 SRR12363900_1.fastq -2 SRR12363900_2.fastq --end-to-end --
sensitive -p 30 -S 11.sam

bowtie2 -x japonicum_genome -1 SRR12363901_1.fastq -2 SRR12363901_2.fastq --end-to-end --
sensitive -p 30 -S 12.sam

bowtie2 -x japonicum_genome -1 SRR12363902_1.fastq -2 SRR12363902_2.fastq --end-to-end --
sensitive -p 30 -S 13.sam

bowtie2 -x japonicum_genome -1 SRR12363903_1.fastq -2 SRR12363903_2.fastq --end-to-end --
sensitive -p 30 -S 14.sam

bowtie2 -x japonicum_genome -1 SRR12363904_1.fastq -2 SRR12363904_2.fastq --end-to-end --
sensitive -p 30 -S 15.sam

bowtie2 -x japonicum_genome -1 SRR12363905_1.fastq -2 SRR12363905_2.fastq --end-to-end --
sensitive -p 30 -S 16.sam

bowtie2 -x japonicum_genome -1 SRR12363906_1.fastq -2 SRR12363906_2.fastq --end-to-end --
sensitive -p 30 -S 17.sam

bowtie2 -x japonicum_genome -1 SRR12363907_1.fastq -2 SRR12363907_2.fastq --end-to-end --
sensitive -p 30 -S 18.sam

bowtie2 -x japonicum_genome -1 SRR12363908_1.fastq -2 SRR12363908_2.fastq --end-to-end --
sensitive -p 30 -S 19.sam

bowtie2 -x japonicum_genome -1 SRR12363909_1.fastq -2 SRR12363909_2.fastq --end-to-end --
sensitive -p 30 -S 20.sam

bowtie2 -x japonicum_genome -1 SRR12363910_1.fastq -2 SRR12363910_2.fastq --end-to-end --
sensitive -p 30 -S 21.sam

bowtie2 -x japonicum_genome -1 SRR12363911_1.fastq -2 SRR12363911_2.fastq --end-to-end --
sensitive -p 30 -S 22.sam

for ((i=1; i<=22;i++))
do
grep -vw "XS:i" "$i".sam > "$i"_unique.sam
soap.coverage -sam -cvg -i "$i"_unique.sam -onlyuniq -p 8 -refsingle
schistosoma_japonicum.PRJEA34885.WBPS9.genomic.fa -o "$i"_unique.soapcov
done
```

5.3 Converting SAM files to sorted bam

```
module load java
module load bwa
module load samtools
for ((i=19; i<=22;i++))
do
srun samtools view -bS "$i".sam | samtools sort /dev/stdin -o "$i".sorted.bam
done
```

5.4 SNP calling

```
module load java
module load samtools
module load bcftools
module load vcftools

#first index the transcriptome
srun samtools faidx schistosoma_japonicum.PRJEA34885.WBPS9.genomic.fa


#Call SNPs from the BAM alignments
srun bcftools mpileup -a AD,DP,SP -Ou -f schistosoma_japonicum.PRJEA34885.WBPS9.genomic.fa
1.sorted.bam 2.sorted.bam 3.sorted.bam 4.sorted.bam 5.sorted.bam 7.sorted.bam 9.sorted.bam
10.sorted.bam 11.sorted.bam 12.sorted.bam 13.sorted.bam 14.sorted.bam 15.sorted.bam
16.sorted.bam 18.sorted.bam 19.sorted.bam 20.sorted.bam 21.sorted.bam 22.sorted.bam | bcftools
call -v -f GQ,GP -mO z -o head.vcf.gz --threads 30
#filter for quality and coverage
srun vcftools --gzvcf head.vcf.gz --remove-indels --maf 0.1 --max-missing 0.9 --minQ 30 --min-
meanDP 10 --max-meanDP 100 --minDP 10 --maxDP 100 --recode --stdout > head_filtered.vcf
#Filter 2: remove multiallelic
bcftools view --max-alleles 2 --exclude-types indels head_filtered.vcf > head_filtered2.vcf
srun vcftools --vcf head_filtered2.vcf --weir-fst-pop population_1.txt --weir-fst-pop population_2.txt --
out pop1_vs_pop2
```

5.5 Population_1.txt (males)

```
1.sorted.bam
2.sorted.bam
3.sorted.bam
4.sorted.bam
7.sorted.bam
10.sorted.bam
13.sorted.bam
14.sorted.bam
16.sorted.bam
18.sorted.bam
22.sorted.bam
```

5.6 population_2.txt (females)

```
5.sorted.bam
9.sorted.bam
11.sorted.bam
12.sorted.bam
15.sorted.bam
19.sorted.bam
20.sorted.bam
21.sorted.bam
```

5.7 mean F_{ST} per scaffold in R

```
fst<-read.table("pop1_vs_pop2.weir.fst", head=T, sep="\t")
fst_scaf<-aggregate(fst$WEIR_AND_COCKERHAM_FST, by=list(scaffold=fst$CHROM), mean)
colnames(fst_scaf)<-c("scaffold", "mean_fst")
write.table(fst_scaf, file="pop1_vs_pop2_MEANfst.txt", quote=F, row.names = F)
```

Supplementary Code 6: CNV analysis with control-FREEC

6.1 Read mapping to *S. japonicum* genome

Bowtie2 (v2.2.9) was used to map two genomic libraries (SRR6841388 for females and SRR6841389 for males) against *S. japonicum* reference genome (schistosoma_japonicum.PRJEA34885.WBPS9.genomic.fa, downloaded from wormbase parasite in Sept. 2017), as described in Picard *et al.*, Elife, 2018 (doi: 10.7554/eLife.35684).

6.2 CNV prediction with control-FREEC

Only mapped reads were extracted from the Bowtie2 output and resulting sam files were sorted before to be used as input for the CNV prediction. The script named *2.controlFREEC.sh* is the same for the three analyses, but the config file changes depending on the used window size (1kb, 5kb or 10kb). A file with the *S. japonicum* length (below named *jap_scaff_length*) is also needed as input.

2.controlFREEC.sh

```
#! /bin/bash

#SBATCH --job-name=controlFREEC
#SBATCH -c 20
#SBATCH --time=72:00:00
#SBATCH --mem=500G

#-# last edit: 12-4-21

##MODULE(S)

module load samtools/1.11

##VARIABLE(S)

config="3.controlFREE.config"

###START

srun FREEC-11.6/src/freec -conf ${config}

###END
```

3.controlFREEC.config

```
[general]

maxThreads=20
chrLenFile=~/path-to-input-file/jap_scaff_length
ploidy=1,2
window=1000 #here change for 5000 or 10000 if needed
```

```
[sample]

mateFile=~/path-to-bowtie2output/japonicum_females.sam
inputFormat=SAM
mateOrientation=FR

[control]

mateFile=~/path-to-bowtie2output/japonicum_males.sam
inputFormat=SAM
mateOrientation=FR

[BAF]

[target]
```

6.3 Significance assessment

After the CNV prediction test, a significance test was run on the predicted CNVs thanks to a R script provided with control-FREEC and copied below.

4.assess_significance.R [script provided with the controlFREEC-11.6 package]

```
#!/usr/bin/env Rscript

library(rtracklayer)

args <- commandArgs()

dataTable <-read.table(args[5], header=TRUE);
ratio<-data.frame(dataTable)

dataTable <- read.table(args[4], header=FALSE)
cnvs<- data.frame(dataTable)

ratio$Ratio[which(ratio$Ratio==-1)]=NA

cnvs.bed=GRanges(cnvs[,1],IRanges(cnvs[,2],cnvs[,3]))
ratio.bed=GRanges(ratio$Chromosome,IRanges(ratio$Start,ratio$Start),score=ratio$Ratio)

overlaps <- subsetByOverlaps(ratio.bed,cnvs.bed)
normals <- setdiff(ratio.bed,cnvs.bed)
normals <- subsetByOverlaps(ratio.bed,normals)

#mu <- mean(score(normals),na.rm=TRUE)
#sigma<- sd(score(normals),na.rm=TRUE)

#hist(score(normals),n=500,xlim=c(0,2))
#hist(log(score(normals)),n=500,xlim=c(-1,1))

#shapiro.test(score(normals)[which(!is.na(score(normals)))][5001:10000])
#qqnorm (score(normals)[which(!is.na(score(normals)))],ylim=(c(0,10)))
#qqline(score(normals)[which(!is.na(score(normals)))], col = 2)

#shapiro.test(log(score(normals))[which(!is.na(score(normals)))][5001:10000])
#qqnorm (log(score(normals))[which(!is.na(score(normals)))],ylim=(c(-6,10)))
#qqline(log(score(normals))[which(!is.na(score(normals)))], col = 2)
```

```
numberOfCol=length(cnvs)

for (i in c(1:length(cnvs[,1]))) {
  values <- score(subsetByOverlaps(ratio.bed,cnvs.bed[i]))
  #wilcox.test(values,mu=mu)
  W <- function(values,normals){resultw <- try(wilcox.test(values,score(normals)), silent = TRUE)
    if(class(resultw)=="try-error")
return(list("statistic"=NA,"parameter"=NA,"p.value"=NA,"null.value"=NA,"alternative"=NA,"method"
=NA,"data.name"=NA)) else resultw}
  KS <- function(values,normals){resultks <- try(ks.test(values,score(normals)), silent = TRUE)
    if(class(resultks)=="try-error")
return(list("statistic"=NA,"p.value"=NA,"alternative"=NA,"method"=NA,"data.name"=NA)) else
resultks}
  #resultks <- try(KS <- ks.test(values,score(normals)), silent = TRUE)
  #    if(class(resultks)=="try-error") NA) else resultks
  cnvs[i,numberOfCol+1]=W(values,normals)$p.value
  cnvs[i,numberOfCol+2]=KS(values,normals)$p.value
  }

if (numberOfCol==5) {
  names(cnvs)=c("chr","start","end","copy
number","status","WilcoxonRankSumTestPvalue","KolmogorovSmirnovPvalue")
}
if (numberOfCol==7) {
  names(cnvs)=c("chr","start","end","copy
number","status","genotype","uncertainty","WilcoxonRankSumTestPvalue","KolmogorovSmirnovPval
ue")
}
if (numberOfCol==9) {
  names(cnvs)=c("chr","start","end","copy
number","status","genotype","uncertainty","somatic/germline","precentageOfGermline","WilcoxonRa
nkSumTestPvalue","KolmogorovSmirnovPvalue")
}
write.table(cnvs, file=paste(args[4],".p.value.txt",sep=""),sep="\t",quote=F,row.names=F)
```

6.4 Identification of genes overlapping deletions

Only the predicted CNVs with a wilcoxon p-value < 0.05 and a “loss” tag were further analyzed: we identified the genes overlapping the deleted windows thanks to the perl script *5.gene-selector.pl*. This step was done three times, for each window size: the input file named below *“windows-with-loss.csv”* corresponds to the three first columns of the control-FREEC outputs “CNVtable_onlysign_loss_controlFREECoutput.csv” (One output for each window size) provided as Sup. Dataset 16. The input named “japonicum-gene-coordinates.csv” corresponds to a 4 column file (1:Gene name, 2:Scaffold name, 3:Gene start, 4:Gene end) and was generated from schistosoma_japonicum.PRJEA34885.WBPS9.canonical_geneset.gtf

5.gene-selector.pl

```
#!/usr/bin/perl
print "finds all genes in a list of window coordinates\n";
print "input are: a list of coordinates ($start $space $end), and a simplified CDS file ($genename
$chrom $start $end)";
print "usage: perl 5.gene-selector.pl windows-with-loss.csv japonicum-gene-coordinates.csv\n";

my $input = $ARGV[0];
```

```
my $ensembl = $ARGV[1];

open (INPUT, "$input");
open (GENES, "$ensembl");
open (RESULTS, ">>$ensembl.Windows.txt");

while ($line = <INPUT>) {
   push (@windows, $line);
   }


while ($gline = <GENES>)
    {
   chomp $gline;
   chomp $gline;
   $gline =~ s/\n//g;
   ($gene, $chrom, $start, $end)=split(/\t/, $gline);
   if ($gene ne "Gene_ID")
          {
          foreach $window (@windows)
                    {
                    ($winchrom, $winstart, $winend)=split(/\t/, $window);
                    if ($chrom eq $winchrom)
                              {
                              if (($start>$winstart && $start<$winend) || ($end>$winstart &&
$end<$winend) || ($start<$winstart && $end>$winend))
                                        {
                                        print RESULTS "$gline\n";
                                        }
                              else
                                        {

                                        }
                              }
                    }
          }
   else {}

   }
```

6.5 Identification of one-to-one orthologs between *S. japonicum* and *S. mansoni*

The previous step provided us three distinct lists of genes: 70 for 1kb windows, 29 for 5kb windows and 70 for 10kb windows. As those lists overlapped to a small extent, we considered the list of 154 unique genes for subsequent analyses. In order to determine the genomic location of those genes, we established a one-to-one orthology between *S. japonicum* and *S. mansoni* CDS, by running successively *6.blat_1to1.sh* and the perl script *7.bestreciprocalhit.pl* with CDSjap_CDSmans.blat.sorted as argument:

6.blat_1to1.sh

```
#! /bin/bash
#SBATCH --job-name="blat"
#SBATCH --mem=50G
#SBATCH --time=48:00:00
```

```
#SBATCH --output=2.blat_one-to-one.out

#-# last edit: 30-10-2020
#-# usage: qsub 2.blat_one-to-one.sh
#-# prog required: BLAT_v36x1
#-# description: Align with BLAT

## define variables:
CDS_mans="~/path-to-mansoni-genome/
schistosoma_mansoni.PRJEA36577.WBPS14.CDS_transcripts.fa"
CDS_jap="~/path-to-japonicum-genome/schistosoma_japonicum.PRJEA34885.WBPS9.genomic.fa"

### start

module load blat/20170224

blat -q=dnax -t=dnax -minScore=50 ${CDS_jap} ${CDS_mans} CDSjap_CDSmans.blat

sort -k 10 CDSjap_CDSmans.blat > CDSjap_CDSmans.blat.sorted

### end
```

7.bestreciprocalhit.pl

```
#!/usr/bin/perl

my $input = $ARGV[0];

system "sort -k 10 $input > $input.sorted";
open (INPUT, "$input.sorted");

open (RESULTS1, ">$input.besthit");

#print RESULTS1 "Qname\tTname\tsimilarity\talignL\tmismatch\tgaps\tQstart\tQend\tTstart\tTend\
tmatch\tbitscore\n";

$name0="";
$score0=0;
$line0="";

while ($line = <INPUT>) {
($match, $mis, $rep, $N, $Qgapc, $Qgapb, $Tgapc, $Tgapb, $strand, $Qname, $Qsize, $Qstart,
$Qend, $Tname, $Tsize, $Tstart, $Tend, $blockc, $blocksize, $qstarts, $tstarts)=split(/\t/, $line);

    if ($Qname eq $name0)
            {
            if ($match > $score0)
                    {
                    $name0=$Qname;
                    $score0=$match;
                    $line0=$line;
                    }
            else
                    {
                    }

            }
    else
            {
```

```
            print RESULTS1 $line0;
            $name0=$Qname;
           $score0=$match;
           $line0=$line;

            }


    }

print RESULTS1 $line0;

close (RESULTS1);
close (INPUT);



#system "perl -pi -e 's/^[^0-9].*//gi' $input.besthit";
#system "perl -pi -e 's/1match/match/gi' $input.besthit";
system "perl -pi -e 's/^\n//gi' $input.besthit";


system "sort -k 14 $input.besthit > $input.besthit.sorted2";
open (INPUT2, "$input.besthit.sorted2");

open (RESULTS2, ">$input.bestreciprocal");

#print RESULTS2 "Qname\tTname\tsimilarity\talignL\tmismatch\tgaps\tQstart\tQend\tTstart\tTend\
tmatch\tbitscore\n";

$name0="";
$score0=0;
$line0="";

while ($line = <INPUT2>) {
    ($match, $mis, $rep, $N, $Qgapc, $Qgapb, $Tgapc, $Tgapb, $strand, $Qname, $Qsize, $Qstart,
$Qend, $Tname, $Tsize, $Tstart, $Tend, $blockc, $blocksize, $qstarts, $tstarts)=split(/\t/, $line);

    if ($Tname eq $name0)
            {
            if ($match > $score0)
                        {
                        $name0=$Tname;
                        $score0=$match;
                        $line0=$line;
                        }
            else
                        {
                        }

            }
    else
            {
            print RESULTS2 $line0;
            $name0=$Tname;
           $score0=$match;
           $line0=$line;

            }
```

```
   }
print RESULTS2 $line0;

system "rm $input.sorted $input.besthit.sorted2 $input.besthit";
system "perl -pi -e 's/^[^0-9].*//gi' $input.bestreciprocal";
system "perl -pi -e 's/1match/match/gi' $input.bestreciprocal";
system "perl -pi -e 's/^\n//gi' $input.bestreciprocal";


close (RESULTS2);
close (INPUT2);
```

The final table summarizing the gene loss in females is named GeneLoss_FinalTable.xlsx and corresponds to the Supplementary Dataset 18. The genomic location is based on the one-to-one orthology and the mansoni genome annotation *schistosoma_mansoni.PRJEA36577.WBPS14*. When no ortholog was found, the genomic location was based on the "best location" of the *S. japonicum* scaffold holding this given gene (Picard *et al.*, elife, 2018).

Supplementary Code 7: Estimating the Rates of Evolution of ZW homologs in *S. mansoni*

7.1 Getting ORFs

The final *S. mansoni set* had 42 coding candidates, 30 of which were annotated. We used *genewise* to get the open reading frames for the 22 candidates that were not annotated.

```
module load java

module load wise/2.4.1

module load blat

for (( i = 1; i <= 20; i++ ))

do

sed -n "$i"p  names_for_genewise.txt | cut -f 1 | sort | uniq | ~/seqtk/seqtk subseq
Final_Set_W_candidates_mansoni.fasta /dev/stdin > "$i"_w.fa

sed -n "$i"p  names_for_genewise.txt | cut -f 2 | sort | uniq | /~/seqtk/seqtk subseq
schistosoma_mansoni.PRJEA36577.WBPS14.protein.fa /dev/stdin > "$i"_prot.fa

sed -n "$i"p  names_for_genewise.txt | cut -f 3 | sort | uniq | ~/seqtk/seqtk subseq
schistosoma_mansoni.PRJEA36577.WBPS14.CDS_transcripts.fa /dev/stdin > "$i"_z.fa

genewise -cdna "$i"_prot.fa "$i"_w.fa -both > "$i"_w.CDS
```

```
genewise -cdna "$i"_prot.fa "$i"_z.fa -both > "$i"_z.CDS

cat "$i"_w.CDS "$i"_z.fa > "$i"_final_cds.fasta
done

#formatting the annotated candidates
for (( i = 21; i <= 40; i++ ))

do

sed -n "$i"p  names_for_genewise.txt | cut -f 1 | sort | uniq | ~/seqtk/seqtk subseq
schistosoma_mansoni.PRJEA36577.WBPS14.CDS_transcripts.fa /dev/stdin > "$i"_w.fa

sed -n "$i"p  names_for_genewise.txt | cut -f 3 | sort | uniq | ~/seqtk/seqtk subseq
schistosoma_mansoni.PRJEA36577.WBPS14.CDS_transcripts.fa /dev/stdin > "$i"_z.fa

cat "$i"_w.fa "$i"_z.fa > "$i"_final_cds.fasta
done
```

names_for_genewise.txt

(this is from a *Blat* alignment of the candidates against the mansoni CDS)

```
SM_W_C2035	Smp_165180.1	Smp_165180.1
SM_W_C2123	Smp_302910.1	Smp_302910.1
SM_W_C2223	Smp_200230.1	Smp_200230.1
SM_W_C2253	Smp_082240.1	Smp_082240.1
SM_W_C2311	Smp_190740.1	Smp_190740.1
SM_W_C2357	Smp_006920.1	Smp_006920.1
SM_W_C2377	Smp_020920.2	Smp_336770.1
SM_W_C2383	Smp_318670.1	Smp_006920.1
SM_W_C2387	Smp_019690.1	Smp_019690.1
SM_W_C2389	Smp_320940.2	Smp_158740.1
SM_W_C2397	Smp_320670.1	Smp_169140.1
SM_W_scaffold12	Smp_310950.1	Smp_120320.1
SM_W_scaffold15	Smp_166600.1	Smp_166600.1
SM_W_scaffold16	Smp_317870.1	Smp_022330.1
SM_W_scaffold18	Smp_323380.2	Smp_337410.2
SM_W_scaffold19	Smp_079220.1	Smp_079220.1
SM_W_scaffold3	Smp_324710.1	Smp_092880.1
SM_W_scaffold5	Smp_093100.2	Smp_093100.2
SM_W_scaffold7	Smp_301330.1	Smp_118750.1
SM_W_scaffold8	Smp_007630.1	Smp_007630.1
Smp_045040.1	Smp_045040.1	Smp_316440.1
Smp_146490.1	Smp_146490.1	Smp_319050.1
Smp_303540.2	Smp_303540.2	Smp_031100.1
Smp_308070.1	Smp_308070.1	Smp_324320.1
Smp_312650.1	Smp_312650.1	Smp_093790.1
Smp_318640.1	Smp_318640.1	Smp_139010.1
Smp_318650.1	Smp_318650.1	Smp_340400.1
Smp_318660.1	Smp_318660.1	Smp_174300.1
Smp_318680.1	Smp_318680.1	Smp_125960.1
Smp_318690.1	Smp_318690.1	Smp_031000.1
Smp_318710.1	Smp_318710.1	Smp_158310.1
Smp_320660.1	Smp_320660.1	Smp_332870.1
Smp_320680.1	Smp_320680.1	Smp_169150.1
Smp_324690.1	Smp_324690.1	Smp_022320.1
```

```
Smp_324700.1 Smp_324700.1 Smp_340380.1
Smp_325490.1 Smp_325490.1 Smp_153170.1
Smp_327030.2 Smp_327030.1 Smp_164880.1
Smp_331650.2 Smp_331650.2 Smp_332860.1
Smp_335650.1 Smp_335650.1 Smp_067520.1
Smp_336600.1 Smp_047190.1 Smp_047190.1
```

7.2 TranslatorX

The coding sequences were aligned with the TranslatorX package with the "gblocks" option used to obtain alignment blocks.

```
module load muscle

module load gblocks/0.91b

for file in ~/*_final_cds.fasta

do

perl ~/translatorx_vLocal.pl -i ${file} -o ${file}.fa.tx -t T -g "-s"

done
```

7.3 KaKs calculator

The dN and dS values were obtained with KaKs_calculator2.0 as follows:

```
for file in ~/*nt_cleanali.fasta #TranslatorX output

do

perl parseFastaIntoAXT.pl ${file}
~/KaKs_Calculator2.0/src/KaKs_Calculator -i ${file}.axt -o ${file}.kaks -m NG -m YN

done
```

Supplementary Code 8: Estimating the Rates of Evolution of ZW homologs in *S. japonicum*

8.1 Genewise

We used genewise to get the open reading frames for our candidates based on the *S. mansoni* protein sequences.

```
module load java
module load wise/2.4.1
module load blat
```

```
for (( i = 1; i <= 48; i++ ))
do

sed -n "$i"p  names_for_genewise.txt | cut -f 1 | sort | uniq | ~/seqtk/seqtk subseq
Final_Set_W_Candidates_japonicum.fasta /dev/stdin > "$i"_w.fa

sed -n "$i"p  names_for_genewise.txt | cut -f 2 | sort | uniq | ~/seqtk/seqtk subseq
schistosoma_mansoni.PRJEA36577.WBPS14.protein.fa /dev/stdin > "$i"_prot.fa

sed -n "$i"p  names_for_genewise.txt | cut -f 3 | sort | uniq | ~/seqtk/seqtk subseq
Final_Set_Z_Candidates_japonicum_07_11_2020.fasta /dev/stdin > "$i"_z.fa

genewise -cdna "$i"_prot.fa "$i"_w.fa -both > "$i"_w.CDS

genewise -cdna "$i"_prot.fa "$i"_z.fa -both > "$i"_z.CDS

cat "$i"_w.CDS "$i"_z.CDS > "$i"_final_cds.fasta
done
```

names_for_genewise.txt

(from blat of *S. japonicum* W-candidates against *S. mansoni* CDS)

```
SJ_W_C10082    Smp_335120.2  TRINITY_DN139669_c0_g1_i3
SJ_W_C10114    Smp_025340.1  Contig12941
SJ_W_C10144    Smp_025360.1  Contig280
SJ_W_C10288    Smp_025510.1  Contig17963
SJ_W_C10290    Smp_025570.1  TRINITY_DN4507_c0_g1_i1
SJ_W_C10320    Smp_076530.1  TRINITY_DN2878_c0_g1_i1
SJ_W_C10324    Smp_135870.1  Contig14966
SJ_W_C10348    Smp_341020.1  TRINITY_DN1083_c0_g1_i1
SJ_W_C10370    Smp_305490.2  Contig3659
SJ_W_C10372    Smp_157720.1  Contig2829
SJ_W_C10410    Smp_166600.1  TRINITY_DN1876_c0_g1_i3
SJ_W_C10422    Smp_157720.1  Contig2829
SJ_W_C10426    Smp_135870.1  Contig14966
SJ_W_C10454    Smp_157750.1  TRINITY_DN6547_c0_g1_i4
SJ_W_C10456    Smp_163380.1  TRINITY_DN126437_c0_g1_i1
SJ_W_C10484    Smp_018220.1  TRINITY_DN4507_c0_g1_i1
SJ_W_C9788     Smp_305490.2  Contig12097
SJ_W_C9802     Smp_135870.1  Contig14966
SJ_W_C9944     Smp_305490.2  TRINITY_DN4100_c0_g1_i5
SJ_W_Contig13  Smp_061920.1  Contig5787
SJ_W_Contig14  Smp_065840.1  TRINITY_DN3602_c0_g1_i13
SJ_W_Contig15  Smp_064800.1  Contig18734
SJ_W_Contig17  Smp_157670.1  TRINITY_DN3218_c0_g1_i1
SJ_W_Contig18  Smp_058650.1  TRINITY_DN233_c0_g1_i11
SJ_W_Contig2   Smp_025560.1  TRINITY_DN2827_c0_g1_i45
SJ_W_Contig23  Smp_157720.1  Contig2829
SJ_W_Contig32  Smp_058650.1  TRINITY_DN233_c0_g1_i11
SJ_W_Contig34  Smp_210500.1  Contig1613
SJ_W_Contig35  Smp_157660.1  TRINITY_DN121342_c0_g1_i1
SJ_W_Contig36  Smp_136070.1  Contig17032
SJ_W_Contig38  Smp_064750.2  TRINITY_DN22475_c0_g1_i1
SJ_W_Contig39  Smp_341050.1  TRINITY_DN852_c0_g1_i15
SJ_W_Contig43  Smp_305490.1  Contig3659
SJ_W_Contig44  Smp_065770.1  Contig12202
SJ_W_Contig49  Smp_019690.1  Contig13148
SJ_W_Contig50  Smp_132020.1  TRINITY_DN7611_c0_g1_i5
```

```
SJ_W_Contig51  Smp_157720.1  TRINITY_DN120212_c0_g1_i1
SJ_W_Contig52  Smp_076560.1  Contig1625
SJ_W_Contig55  Smp_176340.1  Contig6308
SJ_W_Contig56  Smp_337390.2  TRINITY_DN47543_c0_g1_i1
SJ_W_Contig58  Smp_018240.1  TRINITY_DN2542_c0_g1_i1
SJ_W_Contig60  Smp_345630.1  Contig15286
SJ_W_Contig63  Smp_340290.1  Contig16146
SJ_W_Contig64  Smp_335120.1  TRINITY_DN139669_c0_g1_i4
SJ_W_Contig68  Smp_158310.1  TRINITY_DN2031_c1_g1_i2
SJ_W_Contig9   Smp_340500.2  TRINITY_DN71110_c0_g1_i1
SJ_W_scaffold14          Smp_157140.1  TRINITY_DN41888_c0_g1_i1
SJ_W_scaffold21          Smp_157720.1  Contig2829
```

8.2 TranslatorX

We aligned the coding sequences using the TranslatorX package with the "gblocks" option used to obtain alignment blocks.

```
module load muscle
module load gblocks/0.91b
for file in ~/*_final_cds.fasta
do
perl ~/translatorx_vLocal.pl -i ${file} -o ${file}.fa.tx -t T -g "-s"
done
```

8.3 KaKs calculator

The dN and dS values were obtained with KaKs_calculator2.0 as follows:

```
for file in ~/*nt_cleanali.fasta #TranslatorX output

do

perl parseFastaIntoAXT.pl ${file}
~/KaKs_Calculator2.0/src/KaKs_Calculator -i ${file}.axt -o ${file}.kaks -m NG -m YN

done
```

Supplementary Code 9: *S. mansoni* Transcriptome Curation for OrthoFinder

The steps used to curate the *S. mansoni* transcriptome for Orthofinder can be found below:

```
#merge the single CDS files from genewise:
for file in ~/final_cds/*_final_cds.fasta

do

cat $file >> mansoni_ZW_CDS.fasta

done

#merge the single CDS files from genewise:
```

```
#collapse the transcriptome using SpliceFinder.pl

perl SpliceFinder_2.pl schistosoma_mansoni.PRJEA36577.WBPS14.CDS_transcripts.fa

#use blat to find the copies of the W-candidates in the transcriptome that need to be removed
module load blat

blat -minScore=31 schistosoma_mansoni.PRJEA36577.WBPS14.CDS_transcripts.fa.long
mansoni_ZW_CDS.fasta ZW_vs_trans.blat -t=dnax -q=dnax

#filter for matches > 100 and & mismatch/(match+mismatch)<'0.05'
cat ZW_vs_trans.blat | awk '($1>100 && ($2/($1+$2+0.00000001))<0.05)' > filtered_all.txt
#remove all the transcripts in filtered_all from the collapsed transcriptome by listing all the
transcripts in remove.txt and using the command below
perl -ne 'if(/^>(\S+)/){$c=!$i{$1}}$c?print:chomp;$i{$_}=1 if @ARGV' remove.txt
schistosoma_mansoni.PRJEA36577.WBPS14.CDS_transcripts.fa.long >
schistosoma_mansoni_clean_CDS.fasta

#add the w-candidates to the clean collapsed transcriptome
cat schistosoma_mansoni_clean_CDS.fasta mansoni_ZW_CDS.fasta >
schistosoma_mansoni_plus_ZW_orthofinder.fasta
```

Supplementary Code 10: *S. japonicum* Transcriptome Curation for OrthoFinder

The steps used to curate the *S. japonicum* transcriptome for Orthofinder can be found below:

```
#merge the the single CDS to have a file with only coding Z and W to use for Orthofinder

for file in ~/final_cds/*_final_cds.fasta

do

cat $file >> japonicum_ZW_CDS.fasta

done

#collapse schistosoma_japonicum.PRJEA34885.WBPS14.CDS_transcripts.fa using SpliceFinder_2.pl

perl SpliceFinder_2.pl schistosoma_japonicum.PRJEA34885.WBPS14.CDS_transcripts.fa

module load blat
#blat japonicum_ZW_CDS.fasta against the collapsed transcriptome
blat -minScore=31 schistosoma_japonicum.PRJEA34885.WBPS14.CDS_transcripts.fa.long
japonicum_ZW_CDS_12_04_2021.fasta ZW_vs_trans.blat -t=dnax -q=dnax

#filter for matches > 100 and & mismatch/(match+mismatch)<'0.05'
cat ZW_vs_trans.blat | awk '($1>100 && ($2/($1+$2+0.00000001))<0.05)' > filtered_all.txt

#remove all the transcripts in filtered_all from the collapsed transcriptome by listing all the
transcripts in remove.txt and using the command below

perl -ne 'if(/^>(\S+)/){$c=!$i{$1}}$c?print:chomp;$i{$_}=1 if @ARGV' remove.txt
schistosoma_japonicum.PRJEA34885.WBPS14.CDS_transcripts.fa.long >
schistosoma_japonicum_clean_CDS.fasta

#add japonicum_ZW_CDS.fasta to the clean collapsed transcriptome
```

```
cat schistosoma_japonicum_clean_CDS.fasta japonicum_ZW_CDS.fasta >
schistosoma_japonicum_plus_ZW_orthofinder.fasta
```

Supplementary Code 11: Running OrthoFinder

```
#translate the transcriptomes
perl GetLongestAA_v1_July2020.pl schistosoma_japonicum_plus_ZW_orthofinder.fasta

perl GetLongestAA_v1_July2020.pl schistosoma_mansoni_plus_ZW_orthofinder.fasta

perl GetLongestAA_v1_July2020.pl  clonorchis_sinensis.PRJNA386618.WBPS14.CDS_transcripts.fa

#place all of the transcriptomes in one file and run OrthoFinder
~/orthofinder/OrthoFinder/orthofinder -f ~/schistosoma
```

Supplementary Code 12: *S. mansoni* Transcriptome Curation for Kallisto

Steps used to curate the *S. mansoni* transcriptome for the expression analysis using Kallisto:

```
#concatenate the final z and w candidates.
cat Final_Set_W_candidates_mansoni.fasta Final_Set_Z_candidates_mansoni.fasta >
Final_ZW_candidates_mansoni.fasta

#use blat to find the copies in the transcriptomes that need to be removed
module load java
module load blat
blat -minScore=31 schistosoma_mansoni.PRJEA36577.WBPS14.CDS_transcripts.fa.long
Final_ZW_candidates_mansoni.fasta ZW_vs_trans.blat -t=dnax -q=dnax

#filter for matches > 100 and & mismatch/(match+mismatch)<'0.05'
cat ZW_vs_trans.blat | awk '($1>100 && ($2/($1+$2+0.00000001))<0.05)' > filtered_all.txt

#remove all the transcripts in filtered_all from the collapsed transcriptome by listing all the
transcripts in remove.txt and using the command below

perl -ne 'if(/^>(\S+)/){$c=!$i{$1}}$c?print:chomp;$i{$_}=1 if @ARGV' remove.txt
schistosoma_mansoni.PRJEA36577.WBPS14.CDS_transcripts.fa.long >
schistosoma_mansoni_clean_CDS.fasta

#add the Final_ZW_candidates_mansoni.fasta to the clean collapsed transcriptome
cat schistosoma_mansoni_clean_CDS.fasta Final_ZW_candidates_mansoni.fasta >
schistosoma_mansoni_CDS_Kallisto.fasta
```

Supplementary Code 13: *S. japonicum* Transcriptome Curation for Kallisto

Steps used to curate the *S. japonicum* transcriptome for the expression analysis:

```
#concatenate the final z and w candidates.
cat Final_Set_W_Candidates_japonicum.fasta Final_Set_Z_Candidates_japonicum.fasta >
Final_Set_ZW_Candidates_japonicum.fasta

#use blat to find the copies in the transcriptomes that need to be removed
module load java
module load blat
blat -minScore=31 schistosoma_japonicum.PRJEA34885.WBPS14.CDS_transcripts.fa.long
Final_Set_ZW_Candidates_japonicum.fasta ZW_vs_clean_trans.blat -t=dnax -q=dnax

#filter for matches > 100 and & mismatch/(match+mismatch)<'0.05'
cat ZW_vs_clean_trans.blat | awk '($1>100 && ($2/($1+$2+0.00000001))<0.05)' > filtered_all.txt

#remove all the transcripts in filtered_all from the collapsed transcriptome by listing all the
transcripts in remove.txt and using the command below

perl -ne 'if(/^>(\S+)/){$c=!$i{$1}}$c?print:chomp;$i{$_}=1 if @ARGV' remove.txt
schistosoma_japonicum.PRJEA34885.WBPS14.CDS_transcripts.fa.long >
schistosoma_japonicum_clean_CDS.fasta

#add the Final_Set_ZW_Candidates_japonicum.fasta to the clean collapsed transcriptome
cat schistosoma_japonicum_clean_CDS.fasta Final_Set_ZW_Candidates_japonicum.fasta >
schistosoma_japonicum_CDS_Kallisto.fasta
```

Supplementary Code 14: Expression analysis with Kallisto

14.1 Expression analysis in *S. mansoni*

1.kallisto_index_mans.sh

The curated *S. mansoni* transcriptome was indexed for subsequent Kallisto analysis.

```
#! /bin/bash

# OPTIONS

#SBATCH --job-name="kallisto_index_mans"
#SBATCH --output=kallisto_index_mans.out
#SBATCH --mem=8G
#SBATCH --time=24:00:00

# MORE INFOS

#-# last edit:  10-05-2021
#-# usage: qsub 1.kallisto_index_mans.sh
#-# prog required:  kallisto/0.43.1
#-# description: kallisto is a program for quantifying abundances of transcripts from bulk and single-
cell RNA-Seq data, or more generally of target sequences using high-throughput sequencing reads.
It is based on the novel idea of pseudoalignment for rapidly determining the compatibility of reads
with targets, without the need for alignment.
#-# more info here: https://pachterlab.github.io/kallisto/about.html

### START
```

```
TRANSCRIPTOME="~/path-to-mansoni-curated-transcriptome/
schistosoma_mansoni_CDS_Kallisto.fasta"
OUF="~/path-to-mansoni-kallisto-output"

mkdir $OUF
cd $OUF

module load kallisto/0.43.1

# Create index
kallisto index -i $OUF/transcripts.idx $TRANSCRIPTOME

### END
```

2.slurm-script-for-siliconer.sh

Bash script for launching Siliconer perl script, and allowing to feed the table containing the list of libraries. It is shown below for *3.Siliconer_mans_SRR.pl*, but was similarly applied for *4.Siliconer_mans_ERR.pl* and *6.Siliconer_jap.pl*

```
#! /bin/bash

#SBATCH --job-name="kallisto_mans_SRR"
#SBATCH --output=kallisto_mans_SRR.out
#SBATCH --mem=50G
#SBATCH --time=72:00:00

#Input with the library list: RUNlist.txt is a text file with a library number per line

mkdir ~/path-to-mansoni-kallisto-output/KalFiles #creating output folder
module load SRA-Toolkit/2.8.1-3
module load kallisto/0.43.1

#job
srun perl 3.Siliconer_mans_SRR.pl RUNlist.txt
```

3.Siliconer_mans_SRR.pl

Script allowing the automatized download and Kallisto analysis for a given list of libraries. If single-end reads, the fragment size should be adapted on the Kallisto command line! For mansoni SRR libraries used here: [-l 400 -s 40]

```
#!/usr/bin/perl

my $list = $ARGV[0];
open (INPUT, "$list") or die "can't find $input";
while ($run = <INPUT>)
          {
          chomp $run;
          print "$run\n";

### 1. Download fastq files
          #first check if the Kallisto folder already exists
          $filename = "KalFiles/$run";
          if (-e $filename)
                    {
```

```
                    print "$filename exists\n";
                    }
          else
                    {
                    #download only the first 30 million reads of each file so that we don't wait forever
when there are huge ones
                    system "fastq-dump --split-files --skip-technical $run";

### 2. Run Kallisto
                    #check if XXX_2 exists
                    $filename2="$run\_2.fastq";
                    if (-e $filename2)
                              {

                              #if yes
                              system "kallisto quant -t 16 -i
~/path-to-mansoni-kallisto-output/transcripts.idx -o KalFiles/$run -b 100 $run\_1.fastq $run\_2.fastq";
                              }
                    else
                              {

                              #if no
                              print "$run is single-end\n";
                              system "kallisto quant -t 16 -i
~/path-to-mansoni-kallisto-output/transcripts.idx -o KalFiles/$run -b 100 --single -l 400 -s 40 $run\
_1.fastq";
                              }

### 3. remove file
                    system "rm $run\_1.fastq $run\_2.fastq";
                    }
          }
```

4.Siliconer_mans_ERR.pl

Script allowing the automatized download and Kallisto analysis for a given list of libraries

If single-end reads, the fragment size should be adapted on the Kallisto command line! For mansoni ERR libraries used here: [-l 180 -s 20]

```
#!/usr/bin/perl

my $list = $ARGV[0];
open (INPUT, "$list") or die "can't find $input";

while ($run = <INPUT>)
          {
          chomp $run;
          print "$run\n";
### 1. Download fastq files
          #first check if the Kallisto folder already exists
          $filename = "KalFiles/$run";
          if (-e $filename)
                    {
                    print "$filename exists\n";
                    }
          else
                    {
                    #download only the first 30 million reads of each file so that we don't wait forever
```

```
when there are huge ones
                    system "fastq-dump --split-files --skip-technical $run";

### 2. Run Kallisto
                    #check if XXX_2 exists
                    $filename2="$run\_2.fastq";
                    if (-e $filename2)
                              {
                              #if yes
                              system "kallisto quant -t 16 -i
~/path-to-mansoni-kallisto-output/transcripts.id -o KalFiles/$run -b 100 $run\_1.fastq $run\_2.fastq";
                              }
                    else
                              {
                              #if no
                              print "$run is single-end\n";
                              system "kallisto quant -t 16 -i
~/path-to-mansoni-kallisto-output/transcripts.id -o KalFiles/$run -b 100 --single -l 180 -s 20 $run\
_1.fastq";
                              }

### 3. remove file
                    system "rm $run\_1.fastq $run\_2.fastq";
                    }
          }
```

14.2 Expression analysis in *S. japonicum*

5.kallisto_index_jap.sh

The curated *S. japonicum* transcriptome was indexed for subsequent Kallisto analysis.

```
#! /bin/bash

# OPTIONS

#SBATCH --job-name="kallisto_index_jap"
#SBATCH --output=kallisto_index_jap.out
#SBATCH --mem=8G
#SBATCH --time=24:00:00

# MORE INFOS

#-# last edit: 10-05-2021
#-# usage: qsub 5.kallisto_index_jap.sh
#-# prog required: kallisto/0.43.1

### START

TRANSCRIPTOME="~/path-to-japonicum-curated-transcriptome/
schistosoma_japonicum_CDS_Kallisto.fasta"
OUF="~/path-to-japonicum-kallisto-output"

mkdir $OUF
cd $OUF

module load  kallisto/0.43.1
```

```
# Create index

kallisto index -i $OUF/transcripts.idx $TRANSCRIPTOME

### END
```

6.Siliconer_jap.pl

Script allowing the automatized download and Kallisto analysis for a given list of libraries

```
#!/usr/bin/perl

my $list = $ARGV[0];
open (INPUT, "$list") or die "can't find $input";

while ($run = <INPUT>)
	{
	chomp $run;
	print "$run\n";
### 1. Download fastq files
	#first check if the Kallisto folder already exists
	$filename = "KalFiles/$run";
	if (-e $filename)
		{
		print "$filename exists\n";
		}
	else
		{
		#download only the first 30 million reads of each file so that we don't wait forever
when there are huge ones
		system "fastq-dump --split-files --skip-technical $run";

### 2. Run Kallisto

		#check if XXX_2 exists
		$filename2="$run\_2.fastq";
		if (-e $filename2)
			{

			#if yes
			system "kallisto quant -t 16 -i
~/path-to-japonicum-kallisto-output/transcripts.idx -o KalFiles/$run -b 100 $run\_1.fastq $run\
_2.fastq";
			}
		else
			{

			#if no
			print "$run is single-end\n";
			system "kallisto quant -t 16 -i
~/path-to-japonicum-kallisto-output/transcripts.idx -o KalFiles/$run -b 100 --single -l 180 -s 20 $run\
_1.fastq";
			}

### 3. remove file
		system "rm $run\_1.fastq $run\_2.fastq";
		}
}
```
